# Antitumor Activity of Tetrahydro-β-carboline Derivatives via Inhibition of Kinesin Spindle Protein: Validation by Molecular Docking, Molecular Dynamics, and In Vitro Assays

**DOI:** 10.3390/ijms26115396

**Published:** 2025-06-04

**Authors:** Saizhen Guo, Ming Zhang, Xingyuan Zhang, Wenjuan Yuan, Chengting Zi, Zemin Xiang, Yongkai Xi

**Affiliations:** 1 College of Science, Yunnan Agricultural University, Kunming 650201, China; 15608808921@163.com (S.G.); 18445683960@163.com (M.Z.); zhangxingyuan@163.com (X.Z.); yuanwj0805@126.com (W.Y.); 2 College of Food Science and Technology, Yunnan Agricultural University, Kunming 650201, China; zichengting@126.com

**Keywords:** tetrahydro-β-carboline heterocyclic derivatives, antitumor, human lung adenocarcinoma(A549) cells, Eg5 protein

## Abstract

The tetrahydro-β-carboline heterocycle is a privileged scaffold found in numerous natural products and bioactive drugs, demonstrating significant potential for cancer therapy. In this study, we designed and synthesized **33** novel tetrahydro-β-carboline derivatives (**2**–**34**) based on this core structure and evaluated their anticancer activity against human lung cancer (A549). Among them, compounds **8** and **16** exhibited potent cytotoxicity against A549 cells, effectively suppressing cell migration and colony formation. Mechanistic studies revealed that these compounds promoted apoptosis by upregulating pro-apoptotic Bax, downregulating anti-apoptotic Bcl-2, and activating caspase proteins. Molecular docking and dynamics simulations demonstrated that compounds **8** and **16** form stable complexes with the Eg5 protein through multiple hydrogen bonds, which was further validated by thermal shift assays. Collectively, these findings indicate that compounds **8** and **16** induce apoptosis in A549 cells by selectively targeting and stabilizing Eg5, highlighting their potential as lead candidates for lung cancer therapy.

## 1. Introduction

Cancer, as a serious disease, is one of the leading causes of human mortality. Although chemotherapy drugs are effective against cancer, many of them not only target tumor cells but also harm normal cells, leading to adverse effects in patients [1,2,3,4]. To improve the survival rate of cancer patients, a number of pharmacochemists are working on the design and synthesis of new chemical structures to screen for antitumor drugs. Since the early 20th century, significant progress has been made in the development of molecularly targeted anti-tumor drugs. However, today, the development of antitumor drugs still faces many challenges, such as low inhibitory activity, high toxicity, poor selectivity, and drug resistance [5,6,7,8,9]. Therefore, we expect to develop rational, highly efficient, and low-toxic antitumor drugs with good targeting properties.

**Figure 1:**
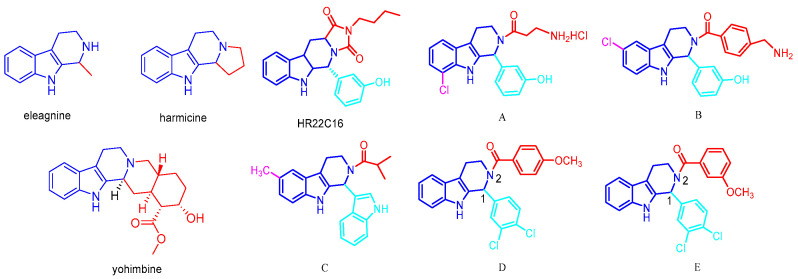
Representative reported tetrahydro-β-carboline alkaloids, Eg5 inhibitors, and ABCG2 inhibitors.

Tetrahydro-β-carboline analogs are indole alkaloids widely found in nature, mainly in plants and marine organisms. The tetrahydro-β-carboline heterocyclic structure is the structural unit of many natural products and important drugs. Natural products and drugs with a tetrahydro-β-carboline skeleton as the core structure include eleagnine [10], harmicine [11], yohimbine [12], and a wide variety of others. A number of natural products and synthetic molecules with a tetrahydro-β-carboline skeleton as their core structure possess a wide range of biological activities and pharmacological effects, such as antitumor [13], antiviral [14], antibacterial [15], and antiplatelet coagulation activities [16]. The representative tetrahydro-β-carboline analogs that have been discovered for their anti-tumor mechanism are as follows: inhibitors of mitotic kinesin Eg5 and inhibitors of breast cancer resistant ATP-binding cassette sub-family G member 2(ABCG2). HR22C16 is a small-molecule compound that was identified as a potent inhibitor of Eg5 (Kinesin Spindle Protein) by high-throughput screening from a library of 16,000 small-molecule compounds [17,18]. Fei Liu et al. [19] designed a series of Eg5 inhibitors, demonstrating that C6/C8-substituted derivatives (compounds A-C) significantly suppressed the ATPase activity of kinesin spindle protein (KSP/Eg5) in tumor cells, with notable enhancement of antiproliferative potency. Parallel work by Anna Spindler et al. [20] identified two tetrahydro-β-carboline derivatives 1-(3,4-Dichlorophenyl)-2,3,4,9-tetrahydro-1H-pyrido [3,4-b]indole and 1-(3,4-Dichlorophe nyl)-6-methoxy-2,3,4,9-tetrahydro-1H-pyrido [3,4-b]indole (D and E) from **37** synthesized analogs that exhibited ABCG2 inhibitory activity comparable to Ko143 (a well-established inhibitor of ABCG2, commonly employed as a positive control in studies examining ABCG2 function and in the screening of novel inhibitors). Their study revealed that N2-acyl substitution and C1-modification were critical for both selectivity and potency against ABCG2. The structural formula is shown in Figure 1.

Meanwhile, the mitotic kinesin Eg5 (also known as kinesin spindle protein, KSP) plays a crucial role in spindle assembly and chromosome segregation during mitosis and has thus become a promising molecular target in anticancer drug development. Mechanistically, the inhibition of Eg5 induces mitotic arrest by disrupting bipolar spindle formation, which ultimately triggers apoptosis in proliferating cells. Eg5 was chosen as a target because of its specific overexpression in tumor cells and its critical role in cell division. Eg5 is overexpressed in a variety of malignant tumors and is barely detectable in normal tissues, making it an ideal target for anticancer drugs that can specifically inhibit tumor cells while minimizing the impact on normal cells [21,22,23,24].

Importantly, Eg5 was found in a variety of malignant tumors—including cervical cancer (HeLa), hepatocellular carcinoma (HepG2), non-small cell lung cancer (A549), breast cancer (MDA-MB-231), and colorectal cancer (HCT116)—that were are overexpressed in, and barely detectable in, most normal tissues, highlighting their therapeutic potential. There were significant differences in the expression levels of Eg5 between these cell lines. For example, the A549 cell line showed higher sensitivity to Eg5 inhibitors, which may be related to its higher Eg5 expression level. In contrast, HCT116 and MDA-MB-231 cell lines were less sensitive to Eg5 inhibitors, which may be due to their relatively low Eg5 expression levels or their stronger backup mechanisms to compensate for Eg5 loss of function. In addition, although HeLa and HepG2 cell lines also overexpress Eg5, their response to Eg5 inhibitors may be influenced by the degree of activation of the cell cycle regulatory network and apoptotic pathways. Although the clinical development of Eg5 inhibitors (e.g., ispinesib, filanesib, and litronesib) has faced challenges, including dose-limiting neutropenia, inconsistent efficacy across cancer subtypes, and acquired resistance mechanisms, these limitations highlight the need for novel stents with better selectivity and pharmacokinetic profiles. In the present study, we retained an important scaffold, the tetrahydro-β-carboline backbone, which may be an important scaffold for molecularly targeted antitumor drugs for further modification and study [25,26].

Tetrahydro-β-carbolines are advantageous antitumor skeletons due to their rigid skeleton, multiple modification sites, and good drug-like properties. Constitutive relationship studies have shown that substitution at C6/C8 positions enhances antiproliferative activity, while acylation at the N2 position improves selectivity and pharmacokinetic properties. Notably, the lead compounds exhibited specific inhibition of the Eg5 protein in HeLa cells, which selectively induced apoptosis in tumor cells without interfering with the normal cell cycle.

In this study, we employed five well-characterized human cancer cell lines representing major malignancies: A549 (non-small cell lung adenocarcinoma), HepG2 (hepatocellular carcinoma), HCT116 (colorectal carcinoma), HeLa (cervical adenocarci-noma), and MDA-MB-231 (triple-negative breast cancer). Using these model systems, we first described the detailed synthetic routes of novel tetrahydro-β-carboline derivatives, then systematically evaluated their antiproliferative activities via a 3-(4,5-dimethylthiazol-2-yl)-2,5-diphenyltetrazolium bromide (MTT) assay. In addition, we analyzed the molecular docking and molecular dynamics simulation of the target compounds using autodock-vina 1.1.2 software and Gromacs software to explore the interrelationship between the structure of tetrahydro-β-carboline derivatives and the activity of target proteins. We also verified the pro-apoptotic effects of tetrahydro-β-carboline derivatives by in vitro molecular experiments, which provided a theoretical basis for further research on the development and utilization of tetrahydro-β-carboline.

## 2. Results and Discussion

### 2.1. Chemistry

Pictet–Spengler cyclization was one of the most powerful methods to synthesize the tetrahydro-β-carboline ring system. Utilizing diverse 3-indolylethylamines and ketone or aldehyde as substrates, the core scaffold of tetrahydro-β-carbolines was constructed favorably.

**Figure 2:**
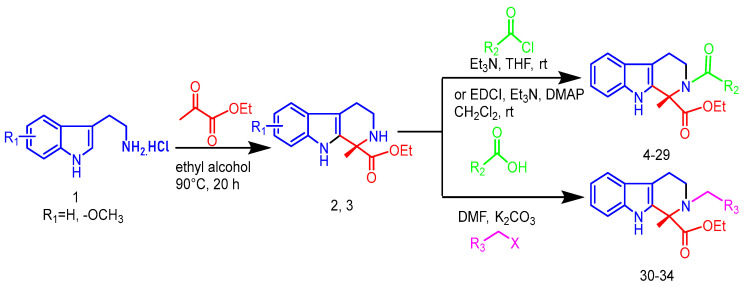
Synthetic route and conditions for the synthesis of tetrahydro-β-carboline analogs.

The synthetic route used to prepare tetrahydro-β-carboline derivatives is shown in Figure 2. Commercially available tryptamine hydrochloride was subjected to Pictet-Spengler cyclization with ethyl pyruvate to give two tetrahydro-β-carboline intermediates, **2** and **3**. Subsequently, intermediates **2** and **3** were subjected to NH site-selective acyl substitution reactions with high-activity organochlorines and low-activity organic acids, respectively, under alkaline conditions. In the specific operation, the regioselective acylation of NH sites was efficiently realized with 1-Ethyl-3-(3-dimethylaminopropyl) carbodiimide (EDCI) as the condenser and 4-Dimethylaminopyridine (DMAP) as the catalyst. A total of **26** structurally defined derivatives were obtained by silica gel column chromatographic separation (**4**–**29**). The above products were further alkylated with halogenated hydrocarbons under K_2_CO_3_ alkaline conditions using N,N-Dimethylformamide (DMF) as solvent. After systematic screening, the targeted synthesis was carried out for five advantageous structures, and finally, five high-purity target derivatives were successfully isolated by silica gel column chromatography (**30**–**34**).

**Figure 3:**
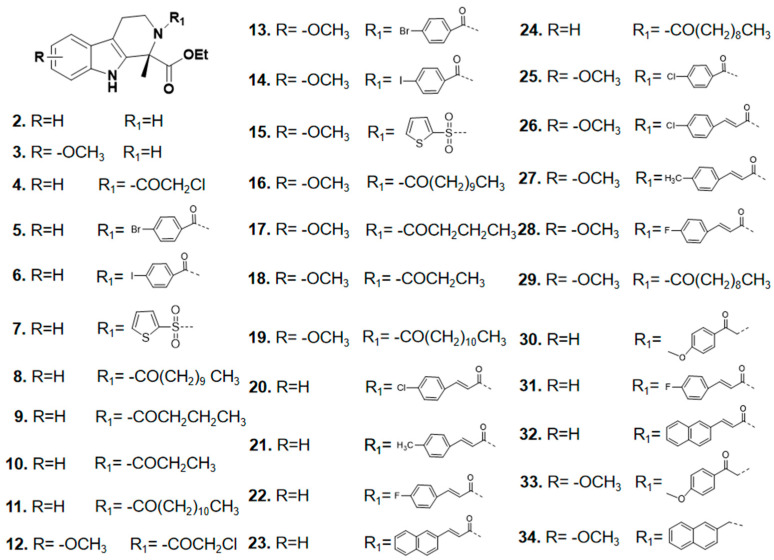
Skeletal structure of tetrahydro-β-carboline analogs.

Ultimately, **34** target compounds were successfully prepared by the above synthetic route and their structural data are listed in Figure 3.

### 2.2. Biological Evaluation

#### 2.2.1. Antiproliferative Activity Screening

The in vitro antiproliferative activity of the novel hybrid compounds **2**–**34** against five human tumor cell lines (cervical cancer HeLa, liver cancer HepG2, lung cancer A549, breast cancer MDA-MB-231, and colon cancer HCT116) was assayed by a Cell Counting Kit-8 (CCK-8) assay (Sigma Aldrich, Burlington, MA, USA) and the kinesin spindle inhibitor ARRY-520 was used as a positive control [27,28,29]. As shown in Table 1, the IC_50_ values varied significantly across cell lines. Notably, compounds **8**, **16**, **19**, and **32** demonstrated potent inhibitory effects against A549 lung cancer cells, with 8 and 16 exhibiting the highest efficacy (IC_50_ = 4.58–5.43 μM). While compounds **20** and **21** showed weaker activity against HepG2 cells and **8**/**16** had limited effects on HCT116 and MDA-MB-231 cells, their exceptional selectivity and potency toward A549 cells, combined with their broad-spectrum potential, highlight them as the most promising candidates for further development as targeted therapies for lung adenocarcinoma.

#### 2.2.2. Compound **8** and Compound **16** Inhibit Colony Formation in A549 Cells

**Figure 4:**
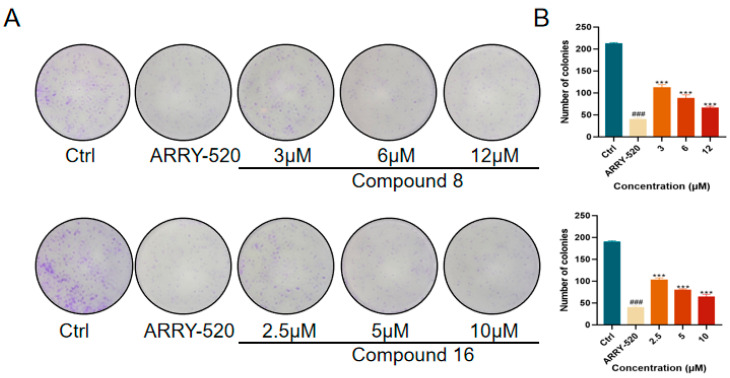
(**A**) Compound **8** and compound **16** inhibit the proliferation of A549 cells. (**B**) Quantification of the number of A549 cells by compounds 8 and 16. Mean ± SEM (n = 4), *** *p* < 0.001 vs. ARRY-520 group, ^###^ *p* < 0.001 vs. Ctrl group.

The effect of compounds **8** and **16** on the proliferative ability of human lung adenocarcinoma cell line A549 cells was examined in this study using the plate cloning crystal violet assay to detect the effect of compounds **8** and **16** on the cloning and proliferative ability of A549 cells. The results showed that, as shown in Figure 4, ARRY-520-positive drugs significantly inhibited the clonogenic and proliferative abilities of A549 cells compared with the control group. Compound **8** and compound **16** inhibited the proliferation of A549 cells in a dose-dependent manner compared with the control group. The number of clones significantly decreased with increasing drug concentration.

#### 2.2.3. Compound **8** and Compound **16** Inhibited the Migration of A549 Cells

**Figure 5:**
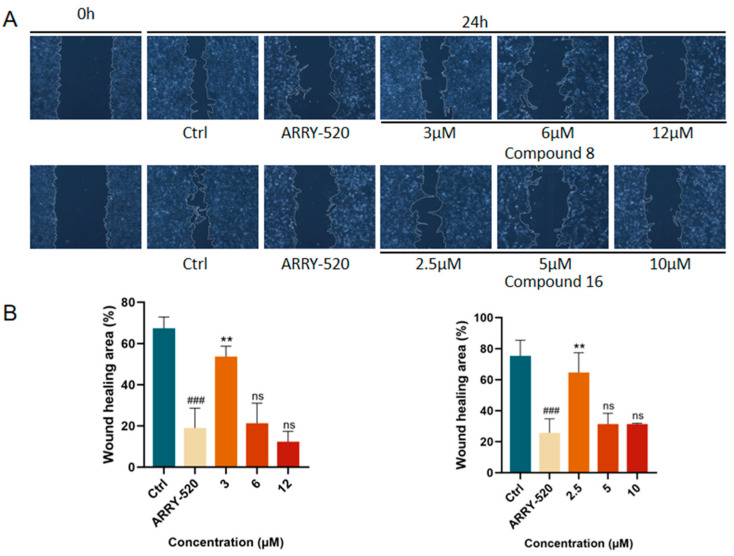
(**A**) Compound **8** and compound **16** effectively inhibited the migration of A549 cells in the wound-healing assay.(**B**) Quantification of the migration area of A549 cells by compounds 8 and 16. Mean ± SEM (n = 4), ** *p* < 0.001 vs. ARRY-520 group, ^###^ *p* < 0.001 vs. Ctrl group.

The occurrence of the invasion and metastasis of malignant tumors is one of the recurrent situations that hinder the treatment of cancer. Migration serves as a necessary condition for invasion and metastasis, and inhibition of tumor cell migration can reduce the risk of malignant tumor recurrence and spread. We used the cell scratch assay to verify the effects of compound **8** and compound **16** on the migration ability of A549 cells. The results showed that, as shown in Figure 5, ARRY-520-positive drugs significantly inhibited the migration of A549 lung cancer cells compared with the control group. The inhibition of A549 cell migration by compound **8** and compound **16** showed a dose-dependent effect compared with the control group. The degree of scratch healing significantly decreased with increasing drug concentration.

#### 2.2.4. Compound **8** and Compound **16** Promote Apoptosis in A549 Cells

**Figure 6:**
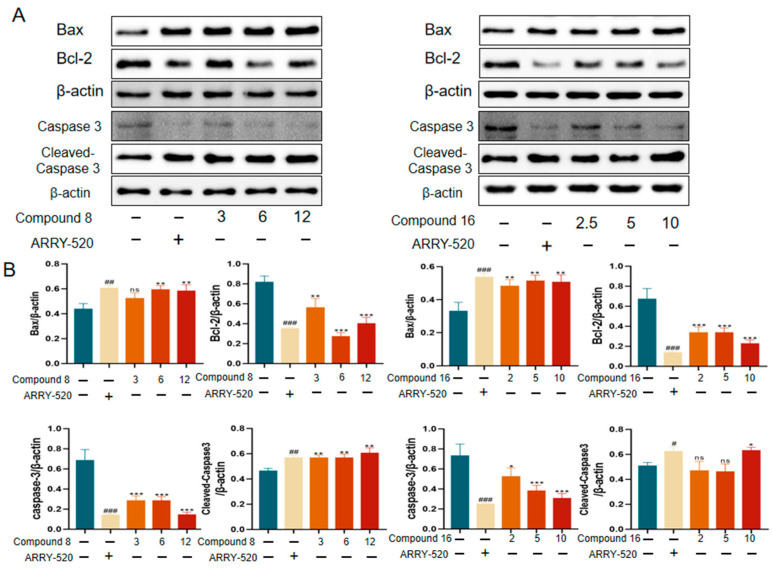
Effect of compound **8** and compound **16** on apoptosis in the A549 cell line. (**A**) Bax, Bcl-2, caspase 3, cleaved-caspase 3 Western grayscale blot. (**B**) Bax, Bcl-2, caspase 3, and cleaved-caspase 3 Western protein expression levels. Mean ± SEM (n = 4), * *p* < 0.05, ** *p* < 0.01, *** *p* < 0.001 vs. ARRY-520 group, ns, ^#^ *p* < 0.05, ^##^ *p* < 0.01, ^###^ *p* < 0.001 vs. Ctrl group.

The induction of apoptosis or autophagy in tumor cells is an important strategy for antitumor therapy. Among them, the mitochondrial apoptotic pathway can be assessed by detecting the expression levels of key regulators: changes in the ratio of the pro-apoptotic protein Bax to the anti-apoptotic protein Bcl-2 reflect the apoptotic status. This pathway ultimately executes the apoptotic program by activating the protein hydrolysis activity of the downstream caspase family (especially caspase-3). We used Western Blot (WB) experiments to validate compound **8** and compound **16** for the detection of apoptotic protein expression levels in A549 cells. The results are shown in Figure 6, where ARRY-520-positive drugs significantly promoted Bax protein expression and inhibited Bcl-2 expression compared with the control group. Compound **8** and Compound **16** promoted Bax protein and inhibited Bcl-2 expression compared to the control. Simultaneous activation of caspase proteins executes the apoptotic program. 

#### 2.2.5. Molecular Docking Studies

**Figure 7:**
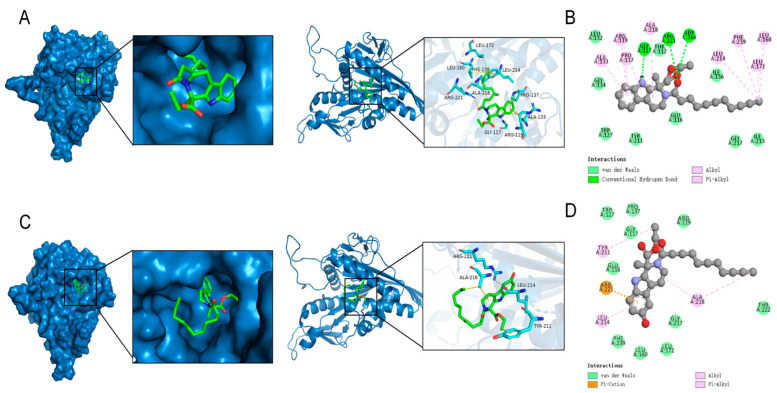
Modeling of the docking of compound **8** and compound **16** with Eg5. (**A**) The 3D model of the interaction of compound **8** with Eg5; (**B**) The 2D model of the interaction of compound **8** with Eg5. (**C**) The 3D model of compound **16** interaction with Eg5. (**D**) The 2D model of compound **16** interacting with Eg5.

Molecular docking is a computational simulation-based method for the study of ligand-receptor interactions, which can be categorized into two strategies: specific docking (based on a known active site) and blind docking (whole protein scanning) [25]. In this study, the specific docking method was used to analyze the docking of the variable binding pocket of the Eg5 protein (PDB ID: 3K5E). AutoDock Vina-1.5.6 software was used and the grid box parameters were set as follows: center coordinates (x = 34.5, y = 8.2, z = 55.3), dimensions (20 × 20 × 20 Å^3^), and coverage of the binding region of the known variant inhibitor. Fifty independent docking calculations were performed for each compound using the Lamarckian genetic algorithm. The docking results showed that the binding energy of compound **8** to Eg5 protein was −7.355 kcal/mol (Figure 7A,B) and that compound **8** hydrogen-bonded to residues GIY 117,ARG 221, and ADP 366 in Eg5 and hydrogen-bonded to residues ALA 133,ARG 119,PRO 137,ALA 218,LEU 214,PHE 239,LEU 160, and LEU 172 residues generate intermolecular forces.

The binding energy of compound **16** to Eg5 protein was −7.396 kcal/mol (Figure 7C,D). Compound **16** produces a Pi-Alkyl interaction with residue ARG 221 and an intermolecular force with residue TYP 221,LEU 214, and ALA 218 in Eg5. Structural analysis suggests that both compounds may effectively inhibit Eg5 protein activity by forming multiple interactions with hydrogen-bonded receptor residues in the binding pocket.

#### 2.2.6. Molecular Dynamics

**Figure 8:**
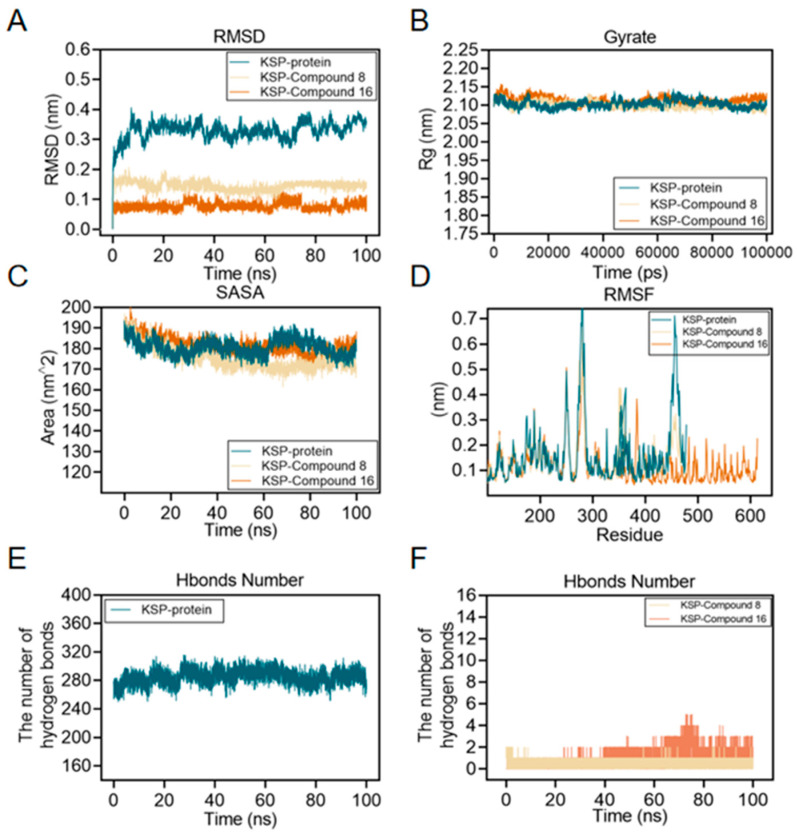
Molecular dynamics simulations of compound **8** and compound **16** with Eg5 were performed using the GROMACS 2002.3 software package and the GAFF force field with a simulation time of 100 ns and a simulation temperature of 300 K. (**A**) RMSD plots of the carbon alpha diagrams of Eg5, compound **8**-Eg5, and compound **16**-Eg5 complexes. (**B**) Rg plots of Eg5, compound **8**-Eg5, and compound **16**-Eg5 complexes. (**C**) RMSF plots of Eg5, compound **8**-Eg5, and compound **16**-Eg5 complexes. (**D**) SASA plot of Eg5, compound **8**-Eg5, and compound **16**-Eg5 complex. (**E**) Hydrogen Bond Number (HBNUM) in the Molecular Dynamics Simulation (MDS) of the Eg5 complex. (**F**) Number of hydrogen bonds formed between Eg5, compound **8**-Eg5, and compound **16**-Eg5 complex.

To verify the stability of the binding of compounds **8** and **16** to Eg5 protein, we performed molecular dynamics simulation analysis. The results show the following. The root mean square deviation (RMSD) values of compounds **8**-Eg5 and **16**-Eg5 complexes were maintained at a low level after 20 ns simulation (Figure 8A), indicating that both of them could significantly improve the structural stability of Eg5 protein; The RMSD and radius of gyration (Rg) of the whole Eg5 system remained relatively stable (Figure 8B); The solvent accessible surface area (SASA) index changed flatly during the 0–100 ns simulation (Figure 8C), further confirming the enhanced overall structural stability of Eg5; Root-mean-square rise and fall (RMSF) analysis revealed that the local conformational dynamics of Eg5 changed less (Figure 8D); Hydrogen bonding analysis revealed (Figure 8E,F) that there were 1–2 stable hydrogen bonding interaction sites between compounds **8/16** and Eg5, while Eg5 itself maintained 1–5 intramolecular hydrogen bonds.

Together, these results suggest that compounds **8** and **16** stabilize the Eg5 protein structure through a specific hydrogen bonding network, providing a structural basis for its inhibitory activity.

#### 2.2.7. Effect of Compound **8** and Compound **16** on Eg5 Stability

**Figure 9:**
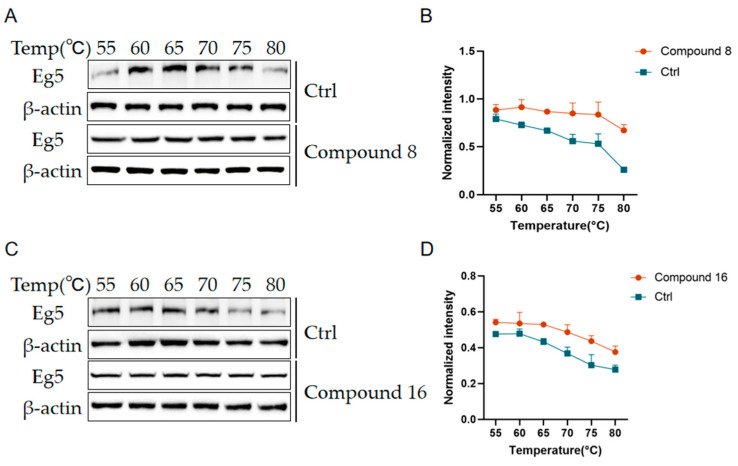
Compound **8** and compound **16** enhance Eg5 stability. (**A**,**C**) Gray scale plots of the Eg5 protein at different temperatures. (**B**,**D**) Expression level analysis of Eg5 protein at different temperatures. Mean ± SEM (n = 3).

To evaluate the binding ability of compounds **8** and **16** to Eg5 protein, we analyzed them using a thermal stability assay(CETSA). After treating A549 cells with 6 μmol/L compound **8** and 5 μmol/L compound **16**, the cells were subjected to thermal denaturation at different temperatures (55–80 °C), and the stability of Eg5 protein was detected by Western Blot. The results are shown in Figure 9 that the Eg5 protein in the compound-treated group exhibited significantly enhanced thermal stability under high temperature conditions compared with the untreated group, indicating that compounds **8** and **16** could effectively bind to the Eg5 protein and improve its structural stability. This result confirms the strong binding ability of compounds **8** and **16** with the Eg5 protein.

## 3. Materials and Methods

### 3.1. Chemistry

All compounds were separated and purified by 200–300 mesh silica gel column chromatography produced by Qingdao Ocean Chemical Group Co. The chemical reagents and solvents used in the experiments were of analytical purity, including tryptamine hydrochloride (Shanghai Haohong Biomedical Technology Co., Ltd., Shanghai, China, purity ≥ 98%), ethyl pyruvate (Beijing Inokai Science and Technology Co., Ltd., Beijing, China, purity ≥ 99%), sodium bicarbonate (Tianjin Daimao Chemical Reagent Factory, Tianjin, China, purity ≥ 99.5%), triethylamine (Tianjin Daimao Chemical Reagent Factory, purity ≥ 99%), chlorine acetyl chloride (Aladdin Chemical Reagent Co., Ltd., Pico Rivera, CA, USA, purity ≥ 98%), n-butyryl chloride (Aladdin Chemical Reagent Co., Ltd.). Acetyl chloride (Aladdin Chemical Reagent Co., Ltd., purity ≥ 98%), n-butyryl chloride (Aladdin Chemical Reagent Co., Ltd., purity ≥ 97%), propionyl chloride (Aladdin Chemical Reagent Co., Ltd., purity ≥ 98%), and deuterated chloroform (Anegi Chemical Reagent Co., Ltd., Shijiazhuang, China, 99.8 atom% D). All reagents were used directly without further purification.

1H NMR: 20 mg of sample was dissolved in CDCl_3_ or DMSO and detected at 400/600 MHz resonance frequency (excitation power 24.5 W), with a relaxation delay of 1 s, sampling time of 4 s, and 16 scans set; 13C NMR: 20 mg of sample was dissolved in CDCl_3_ or DMSO and detected at 100/150 MHz resonance frequency (excitation power 95 W), with a relaxation delay of 2 s, a sampling time of 2 s, and a number of scans of 512. All NMR data were resolved and spectral peaks attributed using MestReNova 14.2 software.

### 3.2. Preparation Tetrahydro-β-carboline Derivatives

Pamine hydrochloride was dissolved in absolute ethanol with stirring, followed by the addition of ethyl pyruvate. The reaction mixture was heated to 90 °C in an oil bath and maintained under reflux for 20 h with TLC monitoring. After completion, the ethanol was removed by rotary evaporation. The residue was treated with NaHCO_3_ solution and extracted with ethyl acetate (3 × 50 mL). The combined organic phases were concentrated under reduced pressure, and the crude product was purified by column chromatography to afford compound **2**–**3** as a solid.

Ethyl(S)-1-methyl-2,3,4,9-tetrahydro-1H-pyrido[3,4-b]indole-1-carboxyla (Compound 2): Light yellow solid; yield, 95%; ^1^H NMR (600 MHz, CDCl_3_) δ 8.32 (s, 1H), 7.53 (d, J = 7.7 Hz, 1H), 7.36 (d, J = 8.0 Hz, 1H), 7.20 (t, J = 7.5 Hz, 1H), 7.12 (t, J = 7.3 Hz, 1H), 4.36–4.17 (m, 2H), 3.22 (ddd, J = 18.2, 12.1, 6.8 Hz, 2H), 2.89–2.68 (m, 2H), 2.55 (s, 1H), 1.73 (s, 3H), 1.32 (t, J = 7.0 Hz, 3H). ^13^C NMR (151 MHz, CDCl_3_) δ 174.33, 136.12, 133.24, 126.97, 122.22, 119.53, 118.58, 111.06, 110.36, 61.98, 58.96, 41.01, 27.31, 22.35, 14.33. ESI-MS: Calcd for C_15_H_19_N_2_O_2_ [M + H]^+^ 259.1441, found 259.1434.

Ethyl(S)-6-methoxy-1-methyl-2,3,4,9-tetrahydro-1H-pyrido[3,4-b]indole-1-carboxylate (Compound 3): Light yellow solid; yield, 94%; ^1^H NMR (400 MHz, CDCl_3_) δ 8.28 (s, 1H), 7.23 (d, J = 8.8 Hz, 1H), 6.96 (d, J = 2.0 Hz, 1H), 6.84 (dd, J = 8.7, 2.3 Hz, 1H), 4.34–4.16 (m, 2H), 3.86 (s, 3H), 3.33–3.13 (m, 2H), 2.82–2.64 (m, 2H), 2.62 (d, J = 23.3 Hz, 1H), 1.71 (s, 3H), 1.31 (t, J = 7.1 Hz, 3H). ^13^C NMR (101 MHz, CDCl_3_) δ 174.29, 154.01, 134.02, 131.15, 127.21, 112.15, 111.79, 110.06, 100.50, 61.97, 58.96, 55.97, 40.98, 27.25, 22.35, 14.30. ESI-MS: Calcd for C_16_H_21_N_2_O_3_ [M + H]^+^ 289.1547, found 289.1540.

In a 25 mL round-bottom flask equipped with a magnetic stir bar, substrate 2 (258 mg, 1.0 mmol) was dissolved in anhydrous tetrahydrofuran (5 mL). To this solution, acyl chloride (1.2 mmol) and triethylamine (0.3 mL, 2.0 mmol) were added sequentially. The reaction mixture was stirred at room temperature and monitored by TLC until completion. The reaction was quenched with saturated sodium bicarbonate solution (10 mL), and the aqueous layer was extracted with ethyl acetate (3 × 15 mL). The combined organic phases were concentrated under reduced pressure, and the crude product was purified by column chromatography to afford eight target compounds (**4**–**11**).

Ethyl(S)-2-(2-chloroacetyl)-1-methyl-2,3,4,9-tetrahydro-1H-pyrido[3,4-b]indole-1-carboxylate (Compound 4): White solid; yield, 78%; ^1^H NMR (600 MHz, CDCl_3_) δ 8.25 (s, 1H), 7.52 (d, J = 7.9 Hz, 1H), 7.35 (d, J = 8.1 Hz, 1H), 7.20 (t, J = 7.6 Hz, 1H), 7.13 (t, J = 7.4 Hz, 1H), 4.26–4.21 (m, 1H), 4.20–4.14 (m, 2H), 3.97 (dp, J = 10.7, 7.2 Hz, 1H), 3.72 (q, J = 7.0 Hz, 1H), 3.61–3.50 (m, 1H), 3.11–3.00 (m, 1H), 3.00–2.88 (m, 1H), 1.89 (s, 3H), 1.12 (t, J = 7.1 Hz, 3H).^13^C NMR (151 MHz, CDCl_3_) δ 171.30, 166.31, 136.37, 132.09, 126.29, 122.81, 120.06, 118.60, 111.50, 109.06, 62.07, 60.53, 43.74, 42.20, 21.55, 21.18, 14.33. ESI-MS: Calcd for C_17_H_19_ClN_2_O_3_ [M + Na]^+^ 357.0976, found 357.0975.

Ethyl(S)-2-(4-bromobenzoyl)-1-methyl-2,3,4,9-tetrahydro-1H-pyrido[3,4-b]indole-1-carboxylate (Compound 5): White solid; yield, 78%; ^1^H NMR (600 MHz, DMSO) δ 11.05 (s, 1H), 7.86 (d, J = 8.5 Hz, 1H), 7.72 (t, J = 8.6 Hz, 3H), 7.44 (t, J = 8.4 Hz, 3H), 7.33 (d, J = 8.1 Hz, 1H), 7.09 (t, J = 7.6 Hz, 1H), 7.00 (t, J = 7.4 Hz, 1H), 4.11–3.86 (m, 2H), 3.40 (dd, J = 17.9, 6.9 Hz, 3H), 2.93–2.72 (m, 2H), 1.91 (s, 3H), 1.02 (t, J = 7.1 Hz, 3H). ^13^C NMR (151 MHz, DMSO) δ 170.02, 168.86, 135.92, 135.04, 132.17, 131.22, 131.14, 130.74, 128.28, 125.23, 122.81, 121.00, 118.30, 117.52, 111.07, 107.40, 60.99, 60.23, 43.84, 39.40, 39.26, 39.12, 38.98, 38.84, 38.70, 38.56, 20.57, 19.99, 13.34. ESI-MS: Calcd for C_22_H_22_BrN_2_O_3_ [M + H]^+^ 441.0808, found 441.0801.

Ethyl(S)-2-(4-iodobenzoyl)-1-methyl-2,3,4,9-tetrahydro-1H-pyrido[3,4-b]indole-1-carboxylate (Compound 6): White solid; yield, 73%; ^1^H NMR (600 MHz, DMSO) δ 11.04 (s, 1H), 7.89 (d, J = 8.0 Hz, 2H), 7.44 (d, J = 7.8 Hz, 1H), 7.33 (d, J = 8.1 Hz, 1H), 7.26 (d, J = 8.0 Hz, 2H), 7.08 (t, J = 7.5 Hz, 1H), 6.99 (t, J = 7.4 Hz, 1H), 4.09–3.86 (m, 2H), 2.95–2.67 (m, 3H), 2.50 (s, 2H), 1.90 (s, 3H), 1.01 (t, J = 7.0 Hz, 3H). ^13^C NMR (151 MHz, DMSO) δ 170.55, 169.60, 137.54, 136.44, 135.85, 132.71, 128.65, 125.76, 121.51, 118.83, 118.04, 111.59, 107.92, 96.85, 61.49, 60.74, 44.35, 21.11, 20.52, 13.86. ESI-MS: Calcd for C_22_H_21_IN_2_O_3_ [M + Na]^+^ 511.0489, found 511.0475.

Ethyl(S)-1-methyl-2-(thiophen-2-ylsulfonyl)-2,3,4,9-tetrahydro-1H-pyrido[3,4-b]indole-1-carboxylate (Compound 7): Light yellow solid; yield, 75%; ^1^H NMR (600 MHz, CDCl_3_) δ 8.48 (s, 1H), 7.86–7.78 (m, 1H), 7.69–7.62 (m, 1H), 7.49 (d, J = 7.8 Hz, 1H), 7.37 (d, J = 8.1 Hz, 1H), 7.24–7.20 (m, 1H), 7.16–7.07 (m, 2H), 4.33 (ddd, J = 14.3, 10.8, 7.1 Hz, 1H), 4.26–4.11 (m, 2H), 3.90–3.80 (m, 1H), 3.50 (ddd, J = 13.1, 9.6, 3.9 Hz, 1H), 2.97–2.78 (m, 3H), 2.11 (s, 3H), 1.26 (dd, J = 14.3, 7.2 Hz, 3H). ^13^C NMR (151 MHz, CDCl_3_) δ 172.51, 141.54, 136.38, 133.62, 132.72, 131.80, 127.14, 126.23, 122.89, 120.00, 118.59, 111.49, 108.67, 63.75, 62.70, 43.04, 24.20, 21.21, 14.06. ESI-MS: Calcd for C_19_H_21_N_2_O_4_S_2_ [M + H]^+^ 405.0937, found 405.0935.

Ethyl(S)-1-methyl-2-undecanoyl-2,3,4,9-tetrahydro-1H-pyrido[3,4-b]indole-1-carboxylate (Compound 8): Light yellow solid; yield, 89%; ^1^H NMR (400 MHz, CDCl_3_) δ 9.33 (s, 1H), 7.55 (d, J = 7.7 Hz, 1H), 7.45 (d, J = 8.0 Hz, 1H), 7.17 (dt, J = 14.7, 7.2 Hz, 2H), 4.20 (tt, J = 13.4, 5.0 Hz, 2H), 3.98 (dq, J = 10.7, 7.1 Hz, 1H), 3.54–3.41 (m, 1H), 3.17–2.85 (m, 2H), 2.66–2.36 (m, 2H), 1.93 (s, 3H), 1.72 (dq, J = 15.1, 7.4 Hz, 2H), 1.31 (s, 14H), 1.12 (t, J = 7.1 Hz, 3H), 0.92 (t, J = 6.7 Hz, 3H). ^13^C NMR (101 MHz, CDCl_3_) δ 172.52, 172.40, 136.35, 132.64, 125.96, 122.07, 119.32, 118.01, 111.56, 108.38, 61.51, 61.28, 42.43, 34.67, 31.74, 29.44, 29.39, 29.32, 29.21, 29.17, 25.12, 22.53, 21.61, 21.07, 13.97, 13.73. ESI-MS: Calcd for C_26_H_39_N_2_O^3^ [M + H]^+^ 427.2955, found 427.2952.

Ethyl(S)-2-butyryl-1-methyl-2,3,4,9-tetrahydro-1H-pyrido[3,4-b]indole-1-carboxylate (Compound 9): White powder; yield, 90%; ^1^H NMR (400 MHz, DMSO) δ 10.93 (s, 1H), 7.44 (d, J = 7.7 Hz, 1H), 7.31 (d, J = 8.0 Hz, 1H), 7.02 (dt, J = 32.6, 7.3 Hz, 2H), 4.16 (d, J = 12.8 Hz, 1H), 3.99 (tt, J = 14.2, 7.0 Hz, 1H), 3.94–3.76 (m, 1H), 3.37 (s, 1H), 3.34–3.23 (m, 1H), 2.79 (s, 2H), 2.44–2.28 (m, 1H), 1.73 (s, 3H), 1.67–1.46 (m, 2H), 0.99 (t, J = 7.0 Hz, 3H), 0.93 (t, J = 7.3 Hz, 3H). ^13^C NMR (101 MHz, DMSO) δ 170.72, 170.17, 135.63, 132.50, 125.00, 120.56, 117.92, 117.25, 110.75, 107.11, 60.07, 59.57, 41.09, 34.87, 20.62, 19.68, 17.44, 13.05, 12.85. ESI-MS: Calcd for C_19_H_24_N_2_O_3_ [M + Na]^+^ 351.1679, found 351.1676.

Ethyl(S)-1-methyl-2-propionyl-2,3,4,9-tetrahydro-1H-pyrido[3,4-b]indole-1-carboxylate (Compound 10): White powder; yield, 88%; ^1^H NMR (400 MHz, CDCl_3_) δ 8.78 (s, 1H), 7.53 (d, J = 7.8 Hz, 1H), 7.41 (d, J = 8.1 Hz, 1H), 7.20 (t, J = 7.5 Hz, 1H), 7.13 (t, J = 7.4 Hz, 1H), 4.27–4.08 (m, 2H), 3.96 (dq, J = 10.7, 7.1 Hz, 1H), 3.53–3.39 (m, 1H), 3.07–2.86 (m, 2H), 2.67–2.41 (m, 2H), 1.90 (s, 3H), 1.22 (t, J = 7.4 Hz, 3H), 1.11 (t, J = 7.1 Hz, 3H). ^13^C NMR (101 MHz, CDCl_3_) δ 173.27, 172.38, 136.43, 132.93, 126.29, 122.44, 119.73, 118.38, 111.65, 108.82, 61.70, 61.44, 42.45, 28.18, 21.78, 21.55, 14.05, 9.47. ESI-MS: Calcd for C_18_H_22_N_2_O_3_ [M + Na]^+^ 337.1523, found 337.1516.

Ethyl(S)-2-dodecanoyl-1-methyl-2,3,4,9-tetrahydro-1H-pyrido[3,4-b]indole-1-carboxylate (Compound 11): White solid; yield, 91%; ^1^H NMR (400 MHz, CDCl_3_) δ 9.42 (s, 1H), 7.56 (d, J = 7.7 Hz, 1H), 7.47 (d, J = 8.0 Hz, 1H), 7.24–7.10 (m, 2H), 4.30–4.15 (m, 2H), 4.00 (dq, J = 10.8, 7.1 Hz, 1H), 3.54–3.40 (m, 1H), 3.12–2.89 (m, 2H), 2.68–2.44 (m, 2H), 1.95 (s, 3H), 1.81–1.70 (m, 2H), 1.32 (s, 16H), 1.14 (t, J = 7.1 Hz, 3H), 0.94 (t, J = 6.8 Hz, 3H). ^13^C NMR (101 MHz, CDCl_3_) δ 172.73, 172.66, 136.69, 133.02, 126.29, 122.37,119.61, 118.31, 111.92, 108.68, 61.78, 61.60, 42.73, 35.01, 32.08, 29.80, 29.79, 29.71, 29.65, 29.54, 29.51, 25.44, 22.86, 21.95, 21.38, 14.30, 14.07. ESI-MS: Calcd for C_27_H_40_N_2_O_3_ [M + Na]^+^ 463.2931, found 463.2924.

In a 25 mL round-bottom flask equipped with a magnetic stir bar, substrate 3 (288 mg, 1.0 mmol) was dissolved in anhydrous tetrahydrofuran (5 mL). To this solution, the corresponding acyl chloride (1.2 mmol) and triethylamine (0.3 mL, 2.0 mmol) were added sequentially. The reaction mixture was stirred at room temperature with TLC monitoring. Upon completion, the reaction was quenched with saturated sodium bicarbonate solution (10 mL) and extracted with ethyl acetate (3 × 15 mL). The combined organic layers were concentrated under reduced pressure, and the crude product was purified by column chromatography to afford eight target compounds (**12**–**19**) in purified form.

Ethyl(S)-2-(2-chloroacetyl)-6-methoxy-1-methyl-2,3,4,9-tetrahydro-1H-pyrido[3,4-b]indole-1-carboxylate (Compound 12): Light yellow solid; yield, 77%; ^1^H NMR (400 MHz, CDCl_3_) δ 8.21 (s, 1H), 7.25 (d, J = 8.6 Hz, 1H), 6.96 (d, J = 2.1 Hz, 1H), 6.87 (dd, J = 8.8, 2.3 Hz, 1H), 4.22 (dd, J = 15.1, 10.7 Hz, 2H), 4.18–4.07 (m, 2H), 3.96 (dq, J = 10.7, 7.1 Hz, 1H), 3.86 (s, 3H), 3.61–3.50 (m, 1H), 3.03 (ddd, J = 15.0, 10.5, 4.5 Hz, 1H), 2.89 (dt, J = 15.1, 3.3 Hz, 1H), 1.88 (s, 3H), 1.12 (t, J = 7.1 Hz, 3H). ^13^C NMR (101 MHz, CDCl_3_) δ 171.52, 166.27, 154.47, 132.76, 131.42, 126.61, 112.77, 112.27, 108.79, 100.61, 62.09, 62.04, 56.09, 43.74, 42.21, 21.61, 21.45, 14.03. ESI-MS: Calcd for C_18_H_22_ClN_2_O_4_ [M + H]^+^ 365.1263, found 365.1265.

Ethyl(S)-2-(4-bromobenzoyl)-6-methoxy-1-methyl-2,3,4,9-tetrahydro-1H-pyrido[3,4-b]indole-1-carboxylate (Compound 13): White solid; yield, 79%; ^1^H NMR (400 MHz, CDCl_3_) δ 8.41 (s, 1H), 7.65–7.54 (m, 2H), 7.38 (d, J = 8.4 Hz, 2H), 7.23 (d, J = 4.3 Hz, 1H), 6.93 (d, J = 2.4 Hz, 1H), 6.84 (dd, J = 8.8, 2.4 Hz, 1H), 4.19 (dq, J = 10.7, 7.1 Hz, 1H), 3.97 (ddt, J = 10.3, 7.6, 5.2 Hz, 2H), 3.83 (s, 3H), 3.52–3.39 (m, 1H), 2.96 (ddd, J = 15.1, 10.6, 4.6 Hz, 1H), 2.76 (dt, J = 15.1, 3.3 Hz, 1H), 2.00 (s, 3H), 1.09 (t, J = 7.1 Hz, 3H). ^13^C NMR (101 MHz, CDCl_3_) δ 171.87, 170.85, 154.46, 135.43, 133.21, 132.09, 131.55, 128.79, 126.76, 124.71, 112.69, 112.32, 108.78, 100.67, 62.11, 62.10, 56.12, 45.01, 21.83, 21.76, 14.11. ESI-MS: Calcd for C_23_H_24_BrN_2_O_4_ [M + H]^+^ 471.0914, found 471.0910.

Ethyl(S)-2-(4-iodobenzoyl)-6-methoxy-1-methyl-2,3,4,9-tetrahydro-1H-pyrido[3,4-b]indole-1-carboxylate (Compound 14): White solid; yield, 81%; ^1^H NMR (400 MHz, DMSO) δ 10.87 (s, 1H), 7.89 (d, J = 8.2 Hz, 2H), 7.26 (d, J = 8.2 Hz, 2H), 7.21 (d, J = 8.8 Hz, 1H), 6.94 (d, J = 2.3 Hz, 1H), 6.73 (dd, J = 8.8, 2.4 Hz, 1H), 4.05 (dq, J = 10.9, 7.1 Hz, 1H), 3.93–3.85 (m, 1H), 3.74 (s, 3H), 3.34 (s, 2H), 2.98–2.64 (m, 2H), 1.88 (s, 3H), 1.01 (t, J = 7.1 Hz, 3H). ^13^C NMR (101 MHz, DMSO) δ 170.58, 169.58, 153.37, 137.56, 135.90, 133.25, 131.50, 128.64, 126.05, 112.28, 111.59, 107.76, 100.03, 96.85, 61.53, 60.73, 55.39, 44.43, 21.20, 20.55, 13.89. ESI-MS: Calcd for C_23_H_24_IN_2_O_4_ [M + H]^+^ 519.0775, found 519.0768.

Ethyl(S)-6-methoxy-1-methyl-2-(thiophen-2-ylsulfonyl)-2,3,4,9-tetrahydro-1H-pyrido[3,4-b]indole-1-carboxylate (Compound 15): Milk white solid; yield, 80%; ^1^H NMR (400 MHz, CDCl_3_) δ 8.57 (s, 1H), 7.80 (dd, J = 3.8, 1.2 Hz, 1H), 7.64 (dd, J = 5.0, 1.2 Hz, 1H), 7.28 (d, J = 8.8 Hz, 1H), 7.10 (dd, J = 4.9, 3.9 Hz, 1H), 6.94 (d, J = 2.3 Hz, 1H), 6.88 (dd, J = 8.8, 2.4 Hz, 1H), 4.33 (dq, J = 10.8, 7.1 Hz, 1H), 4.25–4.10 (m, 1H), 3.85 (s, 3H), 3.55–3.43 (m, 1H), 2.89 (ddd, J = 14.6, 9.6, 4.8 Hz, 1H), 2.86–2.74 (m, 1H), 2.11 (s, 3H), 1.26 (t, J = 7.1 Hz, 3H), 1.01–0.83 (m, 1H). ^13^C NMR (101 MHz, CDCl_3_) δ 172.53, 154.37, 141.47, 133.60, 132.72, 132.47, 131.51, 127.12, 126.53, 112.85, 112.28, 108.32, 100.60, 63.80, 62.67, 56.02, 43.01, 24.07, 21.25, 14.02. ESI-MS: Calcd for C_20_H_23_N_2_O_5_S_2_ [M + H]^+^ 435.1043, found 435.1037.

Ethyl(S)-6-methoxy-1-methyl-2-undecanoyl-2,3,4,9-tetrahydro-1H-pyrido[3,4-b]indole-1-carboxylate (Compound 16): Light yellow solid; yield, 87%; ^1^H NMR (600 MHz, CDCl_3_) δ 8.44–8.11 (m, 1H), 7.28–7.22 (m, 1H), 6.95 (s, 1H), 6.84 (dd, J = 8.7, 2.0 Hz, 1H), 4.14 (ddd, J = 28.1, 17.6, 10.4 Hz, 2H), 3.99–3.92 (m, 1H), 3.85 (s, 3H), 3.50–3.39 (m, 1H), 3.00–2.90 (m, 1H), 2.84 (d, J = 14.9 Hz, 1H), 2.47 (ddt, J = 50.9, 14.9, 7.5 Hz, 2H), 1.85 (s, 3H), 1.74–1.62 (m, 2H), 1.41–1.22 (m, 14H), 1.09 (t, J = 7.1 Hz, 3H), 0.88 (t, J = 6.9 Hz, 3H). ^13^C NMR (151 MHz, CDCl_3_) δ 172.69, 172.29, 154.37, 133.76, 131.49, 126.70, 112.45, 112.28, 108.69, 100.64, 61.69, 61.46, 56.12, 42.67, 34.99, 32.03, 29.72, 29.67, 29.60, 29.50, 29.46, 25.34, 22.81, 21.91, 21.59, 14.25, 14.09. ESI-MS: Calcd for C_27_H_41_N_2_O_4_ [M + H]^+^ 457.3061, found 457.3056.

Ethyl(S)-2-butyryl-6-methoxy-1-methyl-2,3,4,9-tetrahydro-1H-pyrido[3,4-b]indole-1-carboxylate (Compound 17): White solid; yield, 88%; ^1^H NMR (400 MHz, DMSO) δ 10.48 (s, 1H), 6.92 (d, J = 8.7 Hz, 1H), 6.68 (d, J = 2.3 Hz, 1H), 6.44 (dd, J = 8.8, 2.4 Hz, 1H), 3.89 (d, J = 12.9 Hz, 1H), 3.71 (dq, J = 10.9, 7.1 Hz, 1H), 3.59 (d, J = 7.0 Hz, 1H), 3.48 (s, 3H), 3.09 (s, 1H), 3.06–2.95 (m, 1H), 2.21 (dt, J = 15.0, 7.5 Hz, 2H), 2.15–2.04 (m, 1H), 1.45 (s, 3H), 1.29 (h, J = 7.3 Hz, 2H), 0.72 (t, J = 7.1 Hz, 3H), 0.66 (t, J = 7.4 Hz, 3H). ^13^C NMR (101 MHz, DMSO) δ 171.48, 170.95, 153.31, 133.84, 131.45, 126.04, 112.18, 111.38, 107.75, 100.04, 60.89, 60.32, 55.38, 41.94, 35.64, 21.48, 20.49, 18.21, 13.83, 13.61. ESI-MS: Calcd for C_20_H_27_N_2_O_4_ [M + H]^+^ 359.1965, found 359.1959.

Ethyl(S)-6-methoxy-1-methyl-2-propionyl-2,3,4,9-tetrahydro-1H-pyrido[3,4-b]indole-1-carboxylate (Compound 18): White solid; yield, 86%; ^1^H NMR (400 MHz, CDCl_3_) δ 8.75 (s, 1H), 7.29 (d, J = 8.8 Hz, 1H), 6.97 (d, J = 2.4 Hz, 1H), 6.85 (dd, J = 8.8, 2.4 Hz, 1H), 4.24–4.15 (m, 1H), 4.15–4.08 (m, 1H), 3.95 (dq, J = 10.7, 7.1 Hz, 1H), 3.86 (s, 3H), 3.50–3.38 (m, 1H), 3.03–2.82 (m, 2H), 2.64–2.40 (m, 2H), 1.88 (s, 3H), 1.21 (t, J = 7.4 Hz, 3H), 1.10 (t, J = 7.1 Hz, 3H). ^13^C NMR (101 MHz, CDCl_3_) δ 173.24, 172.34, 154.28, 133.68, 131.58, 126.59, 112.36, 108.55, 100.56, 61.66, 61.50, 56.08, 42.47, 28.15, 21.83, 21.51, 14.03, 9.45. ESI-MS: Calcd for C_19_H_25_N_2_O_4_ [M + H]^+^ 345.1809, found 345.1806.

Ethyl(S)-2-dodecanoyl-6-methoxy-1-methyl-2,3,4,9-tetrahydro-1H-pyrido[3,4-b]indole-1-carboxylate (Compound 19): Light yellow green solid; yield, 92%; ^1^H NMR (600 MHz, CDCl_3_) δ 8.65 (s, 1H), 7.28 (d, J = 8.8 Hz, 1H), 6.96 (s, 1H), 6.85 (dd, J = 8.7, 2.1 Hz, 1H), 4.22–4.08 (m, 2H), 3.96 (dq, J = 10.9, 7.1 Hz, 1H), 3.86 (s, 3H), 3.50–3.41 (m, 1H), 2.96 (ddd, J = 15.1, 10.7, 4.5 Hz, 1H), 2.87 (t, J = 12.2 Hz, 1H), 2.53 (dt, J = 15.2, 7.7 Hz, 1H), 2.44 (dt, J = 15.0, 7.5 Hz, 1H), 1.87 (s, 3H), 1.74–1.64 (m, 2H), 1.42–1.24 (m, 16H), 1.10 (t, J = 7.1 Hz, 3H), 0.88 (t, J = 6.9 Hz, 3H). ^13^C NMR (151 MHz, CDCl_3_) δ 172.69, 172.39, 154.29, 133.70, 131.57, 126.61, 112.36, 108.56, 100.56, 61.69, 61.50, 56.08, 42.68, 34.95, 32.01, 29.74, 29.72, 29.64, 29.57, 29.47, 29.45, 25.34, 22.79, 21.91, 21.47, 14.23, 14.04. ESI-MS: Calcd for C_28_H_43_N_2_O_4_ [M + H]^+^ 471.3217, found 471.3215.

Substrate 2 (258 mg, 1.0 mmol) was placed in a 25 mL round-bottom flask equipped with a magnetic stir bar. After adding dichloromethane (5 mL) to dissolve the substrate, the following reagents were added sequentially: organic acid (1.2 mmol), EDCI hydrochloride (287.55 mg, 1.5 mmol), triethylamine (0.3 mL, 2.0 mmol), and DMAP (12.2 mg, 0.1 mmol). The reaction mixture was stirred at room temperature with TLC monitoring until completion. The reaction was quenched with water (10 mL) and extracted with dichloromethane (3 × 15 mL). The combined organic phases were concentrated under reduced pressure, and the crude product was purified by column chromatography to afford six target compounds (**20**–**25**).

Ethyl(S)-2-(4-chlorobenzoyl)-1-methyl-2,3,4,9-tetrahydro-1H-pyrido[3,4-b]indole-1-carboxylate (Compound 20): Light yellow semi-solid; yield,78%; ^1^H NMR (600 MHz, CDCl_3_) δ 8.11 (s, 1H), 7.48 (dt, J = 18.6, 8.0 Hz, 2H), 7.40–7.31 (m, 1H), 7.18 (dt, J = 46.9, 7.3 Hz, 1H), 4.88–3.65 (m, 2H), 3.62–3.43 (m, 1H), 3.37–2.46 (m, 2H), 2.03 (s, 1H), 1.26 (s, 3H), 1.16–1.10 (m, 3H). ^13^C NMR (151 MHz, CDCl_3_) δ 171.75, 170.89, 136.52, 136.35, 134.96, 132.54, 129.75, 129.16, 128.66, 127.99, 122.83, 120.11, 118.61, 111.48, 109.19, 62.09, 45.03, 29.86, 22.85, 21.90, 14.28. ESI-MS: Calcd for C_22_H_22_ClN_2_O_3_ [M + H]^+^ 397.1313, found 397.1306.

Ethyl(S,E)-2-(3-(4-chlorophenyl)acryloyl)-1-methyl-2,3,4,9-tetrahydro-1H-pyrido[3,4-b]indole-1-carboxylate (Compound 21): White solid; yield, 80%; ^1^H NMR (600 MHz, CDCl_3_) δ 8.78 (s, 1H), 7.63 (d, J = 15.5 Hz, 1H), 7.54 (d, J = 7.8 Hz, 1H), 7.49 (d, J = 8.4 Hz, 2H), 7.41 (d, J = 8.1 Hz, 1H), 7.36 (d, J = 8.4 Hz, 2H), 7.20 (t, J = 7.5 Hz, 1H), 7.13 (t, J = 7.4 Hz, 1H), 6.99 (d, J = 15.5 Hz, 1H), 4.33 (d, J = 13.1 Hz, 1H), 4.19 (dq, J = 10.8, 7.1 Hz, 1H), 4.03 (dq, J = 10.8, 7.1 Hz, 1H), 3.66–3.52 (m, 1H), 3.08 (ddd, J = 15.0, 10.6, 4.5 Hz, 1H), 2.95 (dt, J = 14.9, 3.2 Hz, 1H), 2.01 (d, J = 38.8 Hz, 3H), 1.10 (t, J = 7.1 Hz, 3H). ^13^C NMR (151 MHz, CDCl_3_) δ 172.33, 166.80, 141.82, 136.68, 135.90, 133.94, 132.94, 129.42, 129.32, 126.53, 122.78, 120.04, 119.48, 118.66, 111.87, 109.09, 62.25, 62.14, 43.34, 22.09, 21.92, 14.31. ESI-MS: Calcd for C_24_H_24_ClN_2_O_3_ [M + H]^+^ 423.1470, found 423.1465.

Ethyl(S,E)-1-methyl-2-(3-(p-tolyl)acryloyl)-2,3,4,9-tetrahydro-1H-pyrido[3,4-b]indole-1-carboxylate (Compound 22): White solid; yield, 81%; ^1^H NMR (600 MHz, CDCl_3_) δ 9.05 (dd, J = 36.2, 16.3 Hz, 1H), 7.74 (dd, J = 15.4, 2.8 Hz, 1H), 7.61 (d, J = 7.7 Hz, 1H), 7.54 (d, J = 7.9 Hz, 2H), 7.52–7.47 (m, 1H), 7.32–7.23 (m, 3H), 7.20 (t, J = 7.4 Hz, 1H), 7.05 (d, J = 15.4 Hz, 1H), 4.42 (d, J = 12.1 Hz, 1H), 4.33–4.22 (m, 1H), 4.16–4.05 (m, 1H), 3.71–3.60 (m, 1H), 3.22–3.10 (m, 1H), 3.01 (d, J = 14.9 Hz, 1H), 2.43 (d, J = 13.5 Hz, 3H), 2.06 (d, J = 3.4 Hz, 3H), 1.16 (t, J = 7.1 Hz, 3H). ^13^C NMR (151 MHz, CDCl_3_) δ 172.09, 166.78, 142.77, 139.94, 136.30, 132.66, 132.24, 129.44, 127.68, 126.10, 122.23, 119.50, 118.16, 117.32, 111.49, 108.64, 61.77, 61.65, 53.31, 42.81, 21.68, 21.43, 21.29, 13.84. ESI-MS: Calcd for C_25_H_27_N_2_O_3_ [M + H]^+^ 403.2016, found 403.2015.

Ethyl(S,E)-2-(3-(4-fluorophenyl)acryloyl)-1-methyl-2,3,4,9-tetrahydro-1H-pyrido[3,4-b]indole-1-carboxylate (Compound 23): White solid; yield, 84%; ^1^H NMR (600 MHz, CDCl_3_) δ 8.65 (dd, J = 41.6, 15.7 Hz, 1H), 7.66 (d, J = 15.4 Hz, 1H), 7.60–7.52 (m, 3H), 7.45–7.39 (m, 1H), 7.25–7.19 (m, 1H), 7.15 (t, J = 7.4 Hz, 1H), 7.13–7.07 (m, 2H), 6.95 (d, J = 15.4 Hz, 1H), 4.35 (d, J = 12.9 Hz, 1H), 4.20 (dq, J = 10.9, 7.1 Hz, 1H), 4.04 (dq, J = 10.8, 7.1 Hz, 1H), 3.66–3.56 (m, 1H), 3.09 (ddd, J = 15.0, 10.6, 4.4 Hz, 1H), 2.96 (dt, J = 14.9, 3.2 Hz, 1H), 1.99 (s, 3H), 1.12 (t, J = 7.1 Hz, 3H). ^13^C NMR (151 MHz, CDCl_3_) δ 171.91, 166.52, 164.33, 162.67, 141.59, 136.24, 132.59, 131.27, 129.57, 129.52, 126.16, 122.39, 119.66, 118.27, 115.94, 115.79, 111.42, 108.75, 61.79, 61.71, 42.90, 21.69, 21.57, 13.91. ESI-MS: Calcd for C_24_H_24_FN_2_O_3_ [M + H]^+^ 407.1765, found 407.1759.

Ethyl(S,E)-1-methyl-2-(3-(naphthalen-2-yl)acryloyl)-2,3,4,9-tetrahydro-1H-pyrido[3,4-b]indole-1-carboxylate (Compound 24): White solid; yield, 83%; ^1^H NMR (600 MHz, CDCl_3_) δ 8.59 (s, 1H), 8.53 (d, J = 15.2 Hz, 1H), 8.24 (d, J = 8.3 Hz, 1H), 7.90 (t, J = 7.8 Hz, 2H), 7.82 (d, J = 7.1 Hz, 1H), 7.63–7.50 (m, 4H), 7.43 (d, J = 8.1 Hz, 1H), 7.23 (t, J = 7.6 Hz, 1H), 7.16 (t, J = 7.4 Hz, 1H), 7.10 (d, J = 15.2 Hz, 1H), 4.41 (d, J = 13.1 Hz, 1H), 4.26 (dq, J = 10.9, 7.1 Hz, 1H), 4.10 (ddt, J = 21.2, 10.8, 7.1 Hz, 2H), 3.71–3.60 (m, 1H), 3.11 (ddd, J = 15.0, 10.6, 4.5 Hz, 1H), 2.97 (dt, J = 14.9, 3.3 Hz, 1H), 2.05 (d, J = 10.2 Hz, 3H), 1.16 (t, J = 7.1 Hz, 3H). ^13^C NMR (151 MHz, CDCl_3_) δ 172.11, 166.77, 140.22, 136.43, 133.81, 132.91, 132.86, 131.60, 130.15, 128.76, 126.86, 126.41, 126.33, 125.53, 124.74, 123.78, 122.61, 121.64, 119.89, 118.52, 111.59, 109.04, 62.05, 61.94, 60.52, 43.15, 21.91, 14.17. ESI-MS: Calcd for C_28_H_26_N_2_O_3_ [M + Na]^+^ 461.1836, found 461.1823.

Ethyl(S)-2-(10-bromodecanoyl)-1-methyl-2,3,4,9-tetrahydro-1H-pyrido[3,4-b]indole-1-carboxylate (Compound 25): White solid; yield, 92%; ^1^H NMR (600 MHz, CDCl_3_) δ 8.63–8.36 (m, 1H), 7.52 (d, J = 7.8 Hz, 1H), 7.38 (t, J = 5.7 Hz, 1H), 7.19 (t, J = 7.4 Hz, 1H), 7.13 (t, J = 7.4 Hz, 1H), 4.21–4.09 (m, 2H), 4.00–3.92 (m, 1H), 3.50–3.43 (m, 1H), 3.41 (t, J = 6.8 Hz, 2H), 3.03–2.95 (m, 1H), 2.90 (d, J = 14.8 Hz, 1H), 2.48 (ddt, J = 49.7, 15.0, 7.5 Hz, 2H), 1.88 (s, 3H), 1.85 (dd, J = 14.7, 7.2 Hz, 2H), 1.72–1.65 (m, 2H), 1.48–1.28 (m, 10H), 1.10 (t, J = 7.1 Hz, 3H). ^13^C NMR (151 MHz, CDCl_3_) δ 172.63, 172.25, 136.40, 132.97, 126.34, 122.50, 119.83, 118.43, 111.55, 108.91, 61.70, 61.42, 42.64, 34.94, 34.17, 32.91, 29.46, 29.42, 28.84, 28.25, 25.28, 21.85, 21.61, 21.58, 14.08. ESI-MS: Calcd for C_25_H_36_BrN_2_O_3_ [M + H]^+^ 491.1904, found 491.1898.

Substrate 3 (288 mg, 1.0 mmol) was charged into a 25 mL round-bottom flask equipped with a magnetic stir bar and dissolved in anhydrous dichloromethane (5 mL). The reaction was initiated by sequential addition of organic acid (1.2 mmol), EDCI (287.55 mg, 1.5 mmol), triethylamine (0.3 mL, 2.0 mmol), and DMAP (12.2 mg, 0.1 mmol). The mixture was stirred at room temperature with TLC monitoring until complete consumption of the starting material. The reaction was quenched with deionized water (10 mL), and the aqueous layer was extracted with dichloromethane (3 × 15 mL). The combined organic extracts were concentrated in vacuo, and the residue was purified by flash column chromatography (silica gel, gradient elution) to afford five target compounds (**26**–**30**) in 72–85% isolated yields.

Ethyl(S)-2-(4-chlorobenzoyl)-6-methoxy-1-methyl-2,3,4,9-tetrahydro-1H-pyrido[3,4-b]indole-1-carboxylate (Compound 26): Light yellow solid; yield, 80%; ^1^H NMR (400 MHz, CDCl_3_) δ 8.48 (s, 1H), 7.58–7.38 (m, 4H), 7.28 (d, J = 8.8 Hz, 1H), 6.96 (d, J = 2.4 Hz, 1H), 6.87 (dd, J = 8.8, 2.4 Hz, 1H), 4.22 (dq, J = 10.7, 7.1 Hz, 1H), 4.09–3.94 (m, 2H), 3.86 (s, 3H), 3.55–3.42 (m, 1H), 3.00 (ddd, J = 15.1, 10.6, 4.6 Hz, 1H), 2.80 (dt, J = 15.1, 3.3 Hz, 1H), 2.03 (s, 3H), 1.12 (t, J = 7.1 Hz, 3H). ^13^C NMR (101 MHz, CDCl_3_) δ 171.89, 170.81, 154.45, 136.45, 134.95, 133.23, 131.56, 129.13, 128.60, 126.75, 112.67, 112.33, 108.76, 100.65, 62.12, 62.09, 56.11, 45.02, 21.84, 21.75, 14.10. ESI-MS: Calcd for C_23_H_23_ClN_2_O_4_ [M + Na]^+^ 449.1239, found 449.1228.

Ethyl(S,E)-2-(3-(4-chlorophenyl)acryloyl)-6-methoxy-1-methyl-2,3,4,9-tetrahydro-1H-pyrido[3,4-b]indole-1-carboxylate( Compound 27): Light yellow solid; yield, 80%; ^1^H NMR (400 MHz, CDCl_3_) δ 8.12 (s, 1H), 7.61 (d, J = 15.5 Hz, 1H), 7.49 (d, J = 8.5 Hz, 2H), 7.41–7.33 (m, 2H), 7.24 (d, J = 5.0 Hz, 1H), 6.96 (d, J = 2.4 Hz, 2H), 6.86 (dd, J = 8.8, 2.5 Hz, 1H), 4.30 (d, J = 12.4 Hz, 1H), 4.24–3.97 (m, 2H), 3.86 (s, 3H), 3.66–3.53 (m, 1H), 3.03 (ddd, J = 14.9, 10.4, 4.5 Hz, 1H), 2.89 (dt, J = 15.0, 3.5 Hz, 1H), 1.93 (s, 3H), 1.11 (t, J = 7.1 Hz, 3H). ^13^C NMR (101 MHz, CDCl_3_) δ 171.90, 166.61, 154.49, 141.69, 135.75, 133.76, 133.51, 131.43, 129.25, 129.15, 126.77, 119.27, 112.64, 112.22, 108.84, 100.72, 62.04, 61.92, 56.14, 43.17, 29.84, 21.94, 14.16. ESI-MS: Calcd for C_25_H_25_ClN_2_O_4_ [M + Na]^+^ 475.1395, found 475.1379.

Ethyl(S,E)-6-methoxy-1-methyl-2-(3-(p-tolyl)acryloyl)-2,3,4,9-tetrahydro-1H-pyrido[3,4-b]indole-1-carboxylate (Compound 28): Light yellow solid; yield, 82%; ^1^H NMR (400 MHz, CDCl_3_) δ 8.93 (s, 1H), 7.70 (d, J = 15.4 Hz, 1H), 7.49 (d, J = 8.0 Hz, 2H), 7.34 (d, J = 8.8 Hz, 1H), 7.22 (d, J = 8.0 Hz, 2H), 7.01 (dd, J = 8.9, 6.5 Hz, 2H), 6.88 (dd, J = 8.8, 2.4 Hz, 1H), 4.39 (d, J = 13.0 Hz, 1H), 4.30–3.99 (m, 2H), 3.89 (s, 3H), 3.66–3.54 (m, 1H), 3.08 (ddd, J = 15.0, 10.6, 4.4 Hz, 1H), 2.93 (dt, J = 14.9, 3.0 Hz, 1H), 2.40 (s, 3H), 2.00 (s, 3H), 1.12 (t, J = 7.1 Hz, 3H). ^13^C NMR (101 MHz, CDCl_3_) δ 172.26, 166.96, 154.26, 142.97, 140.14, 133.58, 132.43, 131.62, 129.63, 127.88, 126.60, 117.50, 112.41, 112.39, 108.58, 100.53, 62.02, 61.83, 56.05, 43.02, 21.93, 21.60, 21.49, 14.04. ESI-MS: Calcd for C_26_H_29_N_2_O_4_ [M + H]^+^ 433.2122, found 433.2119.

Ethyl(S,E)-2-(3-(4-fluorophenyl)acryloyl)-6-methoxy-1-methyl-2,3,4,9-tetrahydro-1H-pyrido[3,4-b]indole-1-carboxylate (Compound 29): Light yellow solid; yield, 84%; ^1^H NMR (600 MHz, CDCl_3_) δ 8.36 (s, 1H), 7.64 (d, J = 15.4 Hz, 1H), 7.55 (dd, J = 8.0, 5.6 Hz, 2H), 7.28 (d, J = 8.8 Hz, 1H), 7.09 (t, J = 8.5 Hz, 2H), 7.01–6.90 (m, 2H), 6.87 (dd, J = 8.7, 2.2 Hz, 1H), 4.33 (d, J = 13.0 Hz, 1H), 4.16 (tdt, J = 21.5, 14.3, 7.1 Hz, 1H), 4.07–3.98 (m, 1H), 3.87 (s, 3H), 3.60 (t, J = 10.4 Hz, 1H), 3.04 (ddd, J = 14.8, 10.6, 4.4 Hz, 1H), 2.91 (d, J = 14.9 Hz, 1H), 1.96 (s, 3H), 1.11 (t, J = 7.1 Hz, 3H). ^13^C NMR (151 MHz, CDCl_3_) δ 172.03, 166.70, 162.88, 154.42, 141.82, 133.54, 131.49, 129.79, 129.74, 126.71, 118.42, 118.41, 116.15, 116.01, 112.57, 112.29, 108.75, 100.64, 62.03, 61.90, 56.11, 43.12, 21.95, 21.79, 14.14. ESI-MS: Calcd for C_25_H_26_FN_2_O_4_ [M + H]^+^ 437.1871, found 437.1867.

Ethyl(S)-2-(10-bromodecanoyl)-6-methoxy-1-methyl-2,3,4,9-tetrahydro-1H-pyrido[3,4-b]indole-1-carboxylate (Compound 30): White solid; yield, 90%; ^1^H NMR (600 MHz, CDCl_3_) δ 8.36 (dd, J = 41.4, 16.7 Hz, 1H), 7.26 (dd, J = 8.6, 2.0 Hz, 1H), 6.96 (s, 1H), 6.85 (dd, J = 8.8, 2.2 Hz, 1H), 4.20–4.07 (m, 2H), 3.96 (dq, J = 10.8, 7.1 Hz, 1H), 3.85 (s, 3H), 3.48–3.42 (m, 1H), 3.40 (t, J = 6.8 Hz, 2H), 3.00–2.92 (m, 1H), 2.85 (d, J = 14.9 Hz, 1H), 2.52 (dt, J = 15.1, 7.6 Hz, 1H), 2.43 (dt, J = 15.0, 7.4 Hz, 1H), 1.92–1.79 (m, 5H), 1.71–1.64 (m, 2H), 1.47–1.28 (m, 10H), 1.10 (t, J = 7.1 Hz, 3H). ^13^C NMR (151 MHz, CDCl_3_) δ 172.60, 172.22, 154.35, 133.72, 131.46, 126.67, 112.43, 112.26, 108.67, 100.61, 61.68, 61.46, 56.10, 42.65, 34.93, 34.17, 32.91, 29.45, 29.41, 28.83, 28.24, 25.27, 21.90, 21.59, 14.09. ESI-MS: Calcd for C_26_H_38_BrN_2_O_4_ [M + H]^+^ 521.2009, found 521.2007.

Substrate 2 (258 mg, 1.0 mmol) was placed in a 25 mL round-bottom flask equipped with a magnetic stir bar and dissolved in anhydrous N,N-dimethylformamide (5 mL). Potassium carbonate (414 mg, 3.0 mmol) was added, and the mixture was stirred at room temperature for 30 min. Subsequently, the corresponding halide (2.0 mmol) was added portion wise, and the reaction was monitored by TLC until completion. The mixture was quenched with saturated sodium chloride solution (10 mL) and extracted with ethyl acetate (3 × 15 mL). The combined organic layers were concentrated under reduced pressure, and the crude product was purified by flash column chromatography (silica gel, hexanes/ethyl acetate) to afford four target compounds (**31**–**32**) in 65–82% isolated yields.

Ethyl(S)-2-(2-(4-methoxyphenyl)-2-oxoethyl)-1-methyl-2,3,4,9-tetrahydro-1H-pyrido[3,4-b]indole-1-carboxylate (Compound 31): Yellow solid; yield, 69%; ^1^H NMR (400 MHz, CDCl_3_) δ 8.29 (s, 1H), 8.18 (d, J = 8.9 Hz, 1H), 7.48 (d, J = 7.8 Hz, 1H), 7.32 (d, J = 8.0 Hz, 1H), 7.15 (t, J = 7.1 Hz, 1H), 7.07 (t, J = 7.4 Hz, 1H), 6.91 (d, J = 8.9 Hz, 2H), 4.31–4.07 (m, 4H), 3.84 (s, 3H), 3.31–3.18 (m, 1H), 3.06–2.97 (m, 1H), 2.85 (ddd, J = 13.5, 8.3, 5.2 Hz, 1H), 2.72 (dt, J = 15.2, 4.5 Hz, 1H), 2.05 (d, J = 12.7 Hz, 1H), 1.77 (s, 3H), 1.26 (t, J = 7.2 Hz, 3H). ^13^C NMR (101 MHz, CDCl_3_) δ 197.39, 174.30, 164.30, 137.02, 133.90, 131.60, 129.95, 127.56, 122.84, 120.18, 119.28, 114.34, 111.73, 110.78, 64.32, 62.39, 59.05, 56.19, 47.59, 23.70, 22.08, 15.11. ESI-MS: Calcd for C_24_H_27_N_2_O_4_ [M + H]^+^ 407.1965, found 407.1960.

Ethyl(S)-1-methyl-2-(naphthalen-2-ylmethyl)-2,3,4,9-tetrahydro-1H-pyrido[3,4-b]indole-1-carboxylate (Compound 32): Light yellow solid; yield, 86%; ^1^H NMR (400 MHz, CDCl_3_) δ 8.31 (s, 1H), 7.97 (s, 1H), 7.88 (d, J = 8.5 Hz, 3H), 7.76–7.69 (m, 1H), 7.59–7.46 (m, 3H), 7.39 (d, J = 8.1 Hz, 1H), 7.19 (ddd, J = 14.9, 11.2, 7.1 Hz, 2H), 4.36 (dq, J = 10.8, 7.1 Hz, 1H), 4.24 (dq, J = 10.8, 7.1 Hz, 1H), 4.09 (q, J = 14.7 Hz, 2H), 3.27 (dt, J = 12.0, 6.1 Hz, 1H), 3.04–2.94 (m, 1H), 2.78 (dd, J = 8.5, 3.5 Hz, 2H), 1.92 (s, 3H), 1.36 (t, J = 7.1 Hz, 3H). ^13^C NMR (101 MHz, CDCl_3_) δ 174.40, 138.27, 136.36, 133.91, 133.52, 132.93, 128.01, 127.77, 126.99, 126.71, 126.59, 126.03, 125.54, 122.13, 119.53, 118.61, 111.07, 110.02, 63.82, 61.63, 55.25, 44.77, 23.02, 21.83, 14.48. ESI-MS: Calcd for C_26_H_27_N_2_O_2_ [M + H]^+^ 399.2067, found 399.2059.

Substrate 3 (288 mg, 1.0 mmol) was dissolved in anhydrous N,N-dimethylformamide (5 mL) in a 25 mL round-bottom flask equipped with a magnetic stir bar. Potassium carbonate (414 mg, 3.0 mmol) was added, and the mixture was stirred at room temperature for 30 min. The corresponding halide (2.0 mmol) was then added portionwise, and the reaction was monitored by TLC until completion. The reaction was quenched with saturated sodium chloride solution (10 mL) and extracted with ethyl acetate (3 × 15 mL). The combined organic layers were concentrated under reduced pressure, and the crude product was purified by flash column chromatography (silica gel, hexanes/ethyl acetate) to afford three target compounds (**33**–**34**) in 70–88% isolated yields.

Ethyl(S)-6-methoxy-2-(2-(4-methoxyphenyl)-2-oxoethyl)-1-methyl-2,3,4,9-tetrahydro-1H-pyrido[3,4-b]indole-1-carboxylate(Compound 33): Brown solid; yield, 68%; ^1^H NMR (400 MHz, CDCl_3_) δ 8.19 (d, J = 8.8 Hz, 2H), 8.10 (s, 1H), 7.22 (d, J = 8.8 Hz, 1H), 6.93 (t, J = 5.6 Hz, 3H), 6.83 (dd, J = 8.8, 2.3 Hz, 1H), 4.31–4.16 (m, 2H), 4.13 (t, J = 12.2 Hz, 2H), 3.85 (d, J = 7.9 Hz, 6H), 3.33–3.19 (m, 1H), 3.10–2.97 (m, 1H), 2.83 (ddd, J = 13.6, 8.2, 5.2 Hz, 1H), 2.70 (dt, J = 15.1, 4.4 Hz, 1H), 1.77 (s, 3H), 1.28 (t, J = 7.2 Hz, 3H). ^13^C NMR (101 MHz, CDCl_3_) δ 196.68, 173.54, 163.64, 154.16, 134.13, 131.48, 130.93, 129.35, 127.29, 113.68, 112.21, 111.78, 110.01, 100.72, 63.70, 61.68, 58.41, 56.06, 55.54, 46.93, 23.10, 21.50, 14.47. ESI-MS: Calcd for C_25_H_29_N_2_O_5_ [M + H]^+^ 437.2071, found 437.2063.

Ethyl(S)-6-methoxy-1-methyl-2-(naphthalen-2-ylmethyl)-2,3,4,9-tetrahydro-1H-pyrido[3,4-b]indole-1-carboxylate (Compound 34): Yellow green solid; yield, 84%; ^1^H NMR (400 MHz, CDCl_3_) δ 8.25 (s, 1H), 8.00 (s, 1H), 7.98–7.85 (m, 3H), 7.74 (dd, J = 8.5, 1.4 Hz, 1H), 7.60–7.47 (m, 2H), 7.30 (d, J = 8.8 Hz, 1H), 7.04 (d, J = 2.4 Hz, 1H), 6.93 (dd, J = 8.8, 2.5 Hz, 1H), 4.39 (dq, J = 10.8, 7.1 Hz, 1H), 4.27 (dq, J = 10.7, 7.1 Hz, 1H), 4.11 (dd, J = 29.4, 14.7 Hz, 2H), 3.92 (s, 3H), 3.30 (dt, J = 12.1, 6.2 Hz, 1H), 3.07–2.96 (m, 1H), 2.86–2.72 (m, 2H), 1.94 (s, 3H), 1.38 (t, J = 7.1 Hz, 3H). ^13^C NMR (101 MHz, CDCl_3_) δ 174.31, 154.16, 138.30, 134.79, 133.54, 132.94, 131.52, 127.99, 127.77, 127.34, 126.72, 126.59, 126.01, 125.53, 112.13, 111.80, 109.83, 100.71, 63.88, 61.58, 56.04, 55.23, 44.79, 23.03, 21.88, 14.46. ESI-MS: Calcd for C_27_H_29_N_2_O_3_ [M + H]^+^ 429.2173, found 429.2166.

### 3.3. Biology

#### 3.3.1. Cell Culture

The following human cancer cell lines were utilized in this study: lung adenocarcinoma (A549), obtained from the Chinese Academy of Medical Sciences (Kunming, China); hepatocellular carcinoma (HePG2), sourced from the Chinese Academy of Sciences (Kunming, China); colorectal carcinoma (HCT116), provided by Yunnan University (Kunming, China); cervical adenocarcinoma (Hela), acquired from Wuhan Life Science and Technology Co., Ltd. (Wuhan, China); and triple-negative breast cancer (MDA-MB-231), obtained from the Chinese Academy of Sciences (Kunming, China). These cell lines were cultured in RPMI-1640 medium supplemented with 10% fetal bovine serum (FBS), 1% penicillin, and 1% streptomycin (Meilun Biotechnology, Dalian, China) at 37 °C under a 5% CO_2_ atmosphere.

#### 3.3.2. In Vitro Cytotoxic Activity

Cells were seeded in 96-well plates at a density of 1.5 × 10^4^ cells per well. ARRY-520 (a known Eg5 inhibitor) served as the positive control. After 12 h of pre-incubation at 37 °C, test compounds (tetrahydro-β-carboline derivatives) were added at specified concentrations and incubated for 48 h at 37 °C. Cell viability was assessed using CCK-8 reagent: following 1 h incubation, absorbance was measured at 450 nm. Dose–response curves and IC_50_ values were calculated using GraphPad Prism 9.0 software.

#### 3.3.3. Scratch Migration Assay

A549 cells in the logarithmic growth phase were inoculated in 6-well plates at a density of 4 × 10^6^ cells/well. Four dishes were inoculated at each concentration, and when the cell growth density reached 80–90%, the medium was discarded, and a straight line was drawn with the tip of the gun perpendicular to the horizontal line at the bottom of the dish of the 6-well plate. A cell-free biospecific area was delineated, and the cells were then washed three times with phosphate-buffered saline (PBS) to remove detached cells. Different concentrations of the compound **8** (3, 6, and 12 µM) and compound **16** (2.5, 5, and 10 µM) were added to the wells, and the scratch wounds were imaged at 0 and 24 h under a microscope, and these images were analyzed using ImageJ 2024 software.

#### 3.3.4. Colony-Forming Assay

A549 cells were inoculated in 6-well plates at a density of 1 × 10^3^ cells/well. After waiting for the cells to be completely attached to the wall, drug-containing complete medium was added, and the liquid was changed every 3 days. After 9 days, cell cloning occurred when visible cell clusters were observed on the plates. After completing the culture, the cells were fixed with 4% paraformaldehyde, stained with 0.1% crystal violet dye for 2 min, washed three times with PBS, and then dried at room temperature before being photographed and analyzed for the cell colonies using ImageJ software.

#### 3.3.5. Western Blot

A549 cells were cultured in DMEM medium (Corning, Corning, NY, USA) containing 10% fetal bovine serum (Gibco, Milton, UK) and 1% penicillin-streptomycin (HyClone) at 37 °C, 5% CO_2_. When cell fusion reached 80–90%, digestion was performed using 0.25% trypsin-EDTA (Gibco). After being treated with different concentrations of compounds for 24 h, the cells were washed twice with pre-cooled PBS, lysed on ice by adding RIPA lysis buffer containing 0.1% PMSF (Mellen Biotech, Council Bluffs, IA, USA) for 30 min, and the lysates were centrifuged at 4 °C for 15 min at 12,000× *g* to collect the supernatant, and the protein concentration was determined by the BCA method (Thermo Scientific, Waltham, MA, USA) and adjusted to the same level. The protein concentration was determined by the BCA method (Thermo Scientific) and adjusted to the same concentration. In total, 30 μg of total protein was separated by 8–10% gradient SDS-PAGE electrophoresis and transferred to a 0.45 μm PVDF membrane (Millipore, St. Louis, MO USA), closed in 5% skimmed milk for 1 h, and then mixed with primary antibodies (Bax #A12009, Bcl-2 #12789-1-AP, Eg5 #23333-1-AP, β-actin #12282, Caspase3 #19677-1-AP, Cleaved-Caspase3 #10198-1-AP, Caspase7 #27155-1-AP, ABclonal), which were incubated overnight at 4 °C, washed in TBST, and incubated for 1 hr at room temperature with the corresponding HRP-labeled secondary antibodies (anti-mouse IgG #7076 or anti-rabbit IgG #7074, 1: 5000) incubated at room temperature for 1 h. ECL chemiluminescent reagent (Millipore) was developed and analyzed in grayscale by ImageJ.

#### 3.3.6. Molecular Docking

Autodock-Vina was used to detect the binding ability of compounds **8** and **16** to Eg5 target proteins. CHEMDRAW was used to draw three-dimensional (3D) structures of the compounds, and RSCD-PDB was used to obtain protein structure files. The crystal ligands and water molecules were removed from the proteins, hydrogen atoms were added, and default settings were selected for other parameters. When the ligand binds to the receptor, it changes the structural morphology of the receptor and forms a stable ligand-receptor complex system [30].

#### 3.3.7. Molecular Dynamics Simulation

Gromacs2022.3 software was used for molecular dynamics simulation. For small molecule preprocessing, AmberTools22 is used to add the GAFF force field to small molecules, while Gaussian 16W is used to hydrogenate small molecules and calculate the RESP potential. Potential data will be added to the topology file of the molecular dynamics system. The simulation conditions were carried out at a static temperature of 300 K and atmospheric pressure (1 Bar). Amber99sb-ildn was used as a force field, water molecules were used as solvent (Tip 3p water model), and the total charge of the simulation system was neutralized by adding an appropriate number of Na^+^ ions. The simulation system adopts the steepest descent method to minimize the energy and then carries out the isothermal isovolumic ensemble (NVT) equilibrium and isothermal isobaric ensemble (NPT) equilibrium for 100,000 steps, respectively, with the coupling constant of 0.1 ps and the duration of 100ps. Finally, the free molecular dynamics simulation was performed. The process consisted of 5,000,000 steps, the step length was 2fs, and the total duration was 100 ns. After the simulation was completed, the built-in tool of the software was used to analyze the trajectory, and the root-mean-square variance (RMSD), root-mean-square fluctuation (RMSF), and protein rotation radius of each amino acid trajectory were calculated, combined with the free energy (MMGBSA), free energy topography, and other data [31,32].

#### 3.3.8. Statistical Analysis

Data are presented as the means ± standard error of the mean (SEM). One-way analysis of variance (ANOVA) was performed using the prismchs software package. A value of *p* < 0.05 was considered to be statistically significant.

## 4. Conclusions

In this study, we designed and synthesized a series of tetrahydro-β-carboline derivatives and evaluated their antitumor activity against A549, HepG2, HCT116, MCF-7, and MDA-MB-231 cell lines using the CCK-8 assay (with ARRY-520 as a positive control). Among **33** compounds tested, compounds **8** and **16** demonstrated the most potent anticancer effects. These lead compounds significantly inhibited A549 cell proliferation, migration, and colony formation at low concentrations. Mechanistic studies revealed they promoted apoptosis by upregulating Bax, downregulating Bcl-2, and activating caspase-3. Molecular docking studies indicated strong binding to the Eg5 protein domain through multiple hydrogen bonds, while molecular dynamics simulations confirmed enhanced stability of the Eg5-compound complexes in physiological conditions. Our results suggest that despite the higher activity of ARRY-520, tetrahydro-β-carboline derivatives have potential advantages in terms of selectivity and structural optimizability, providing a new direction for the development of highly efficient and low-toxicity therapeutic agents for lung cancer. Compounds **8** and **16** specifically target Eg5 in A549 cells and inhibit its proliferation-inhibiting and apoptosis-inducing activities, making them promising lead compounds for the treatment of lung cancer.

Despite the important findings of this study, several limitations remain that need to be addressed in follow-up work. The current study is limited to in vitro cellular experiments, and in the future, the in vivo antitumor activity of the compounds needs to be verified by establishing an A549 xenograft tumor model and systematically evaluating their potential toxicity to vital organs.

## Figures and Tables

**Table 1:** Antiproliferative activities of selected compounds (IC_50_, µM).

Compound No.	A549	HePG2	HCT116	Hela	MDA-MB-231
1	>40	>40	>40	>40	>40
ARRY-520	1.24	4.2	1.38	3.2	0.17
2	>40	>40	>40	>40	>40
3	>40	>40	>40	>40	>40
4	23.59	23.85	21.66	>40	13.35
5	>40	>40	>40	>40	>40
6	>40	>40	>40	>40	>40
7	>40	>40	>40	>40	>40
8	5.43	>40	31.40	>40	16.79
9	>40	>40	>40	>40	>40
10	>40	>40	>40	>40	>40
11	11.57	>40	>40	>40	>40
12	>40	>40	>40	>40	28.63
13	>40	>40	>40	>40	>40
14	>40	>40	>40	>40	>40
15	>40	>40	>40	>40	>40
16	4.58	>40	24.05	>40	23.62
17	>40	>40	>40	>40	>40
18	>40	>40	>40	>40	>40
19	13.22	>40	>40	>40	>40
20	25.94	22.56	>40	>40	35.65
21	18.24	38.99	>40	>40	39.78
22	>40	>40	>40	>40	>40
23	>40	>40	>40	>40	>40
24	>40	>40	>40	>40	>40
25	>40	>40	>40	>40	>40
26	>40	>40	>40	>40	>40
27	>40	>40	>40	>40	>40
28	>40	>40	>40	>40	>40
29	>40	>40	>40	>40	>40
30	>40	>40	>40	>40	>40
31	>40	>40	>40	>40	>40
32	9.80	>40	>40	>40	>40
33	>40	>40	>40	>40	>40
34	>40	>40	>40	>40	>40

## Data Availability

The original contributions presented in the study are included in the article/Supplementary Materials; further inquiries can be directed to the corresponding authors.

## Supplementary Materials

The following supporting information can be downloaded at https://www.mdpi.com/article/10.3390/ijms26115396/s1.
