# Supplementary material for: Antitumor Activity of Tetrahydro-β-carboline Derivatives via Inhibition of Kinesin Spindle Protein: Validation by Molecular Docking, Molecular Dynamics, and In Vitro Assays
Source: Int J Mol Sci. 2025 Jun 4;26(11):5396. doi: 10.3390/ijms26115396 (PMC12154573; doi:10.3390/ijms26115396)

Design, synthesis and antitumor activity of tetrahydro-β-carbolin-3-carbonylhydrazide derivatives

Ming Zhang [1 2†], Saizhen Guo[1 2†], Xinyuan Zhang[1 2] , Wenjuan Yuan [2], Zemin Xiang[1] and Yongkai Xi [1 2*]

[1] College of Science Yunnan Agricultural University,Kunming 650201, China
[2] College of Food Science and Technology, Yunnan Agricultural University, Kunming 650201, China
* Correspondence:2021062@ynau.edu.cn (Y.X.); Tel.: +886-871-65226191 (Y.X.)

Table of Contents

1. ^{1}H and ^{13}C NMR of compound (**2~34**) …...………...………...……………...…S2
3. Immunoblot grayscale figure…….…………….……...…….……….………....S35

1.^{1}H and ^{13}C NMR of compound (2-34)

Ethyl(S)-1-methyl-2,3,4,9-tetrahydro-1H-pyrido[3,4-b]indole-1-carboxyla (**2**):

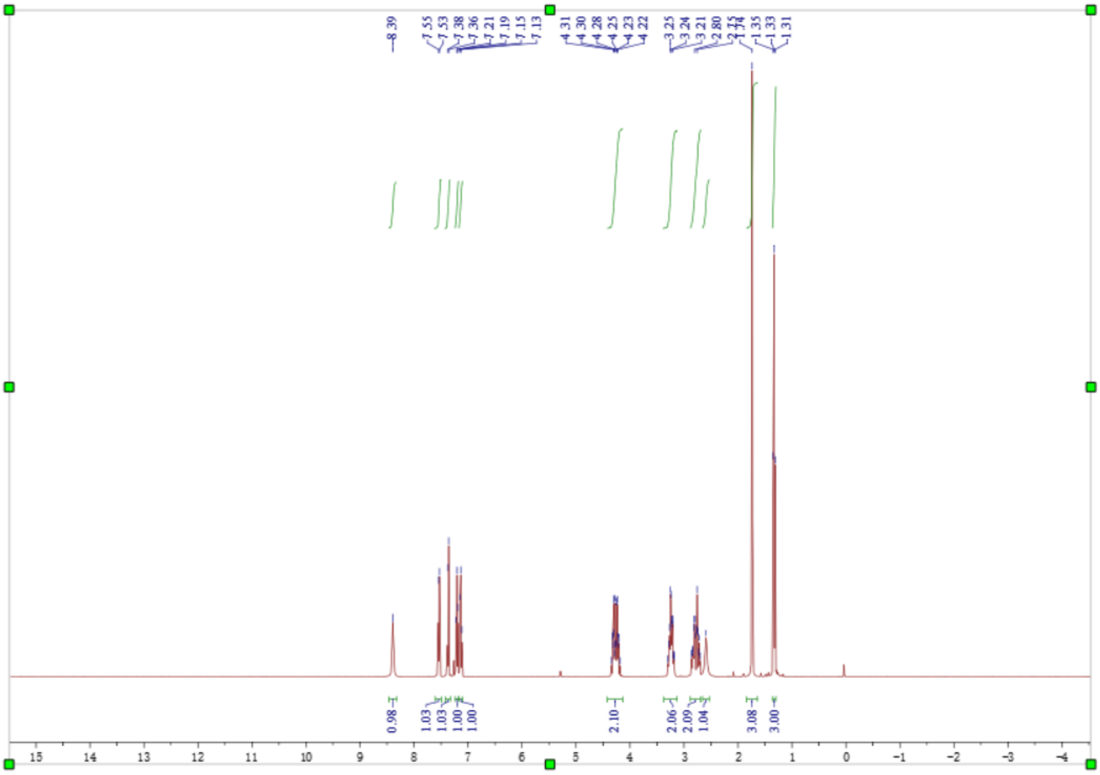

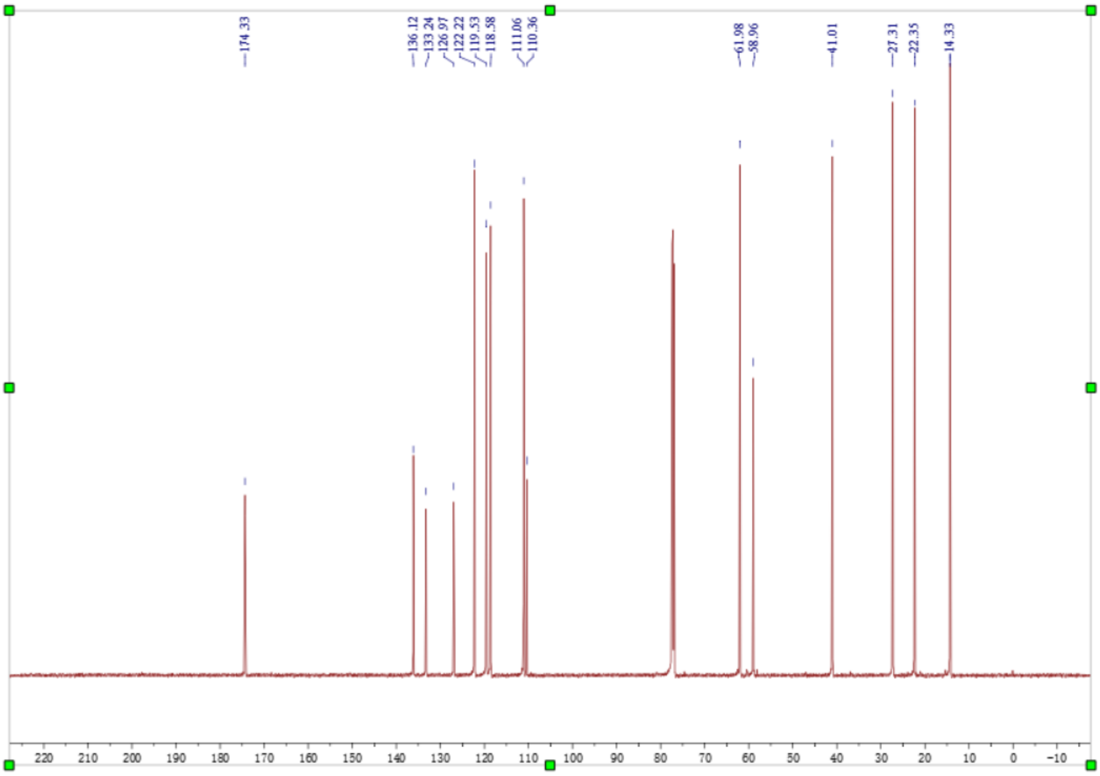

Ethyl(S)-6-methoxy-1-methyl-2,3,4,9-tetrahydro-1H-pyrido[3,4-b]indole-1-carboxylate (**3**):

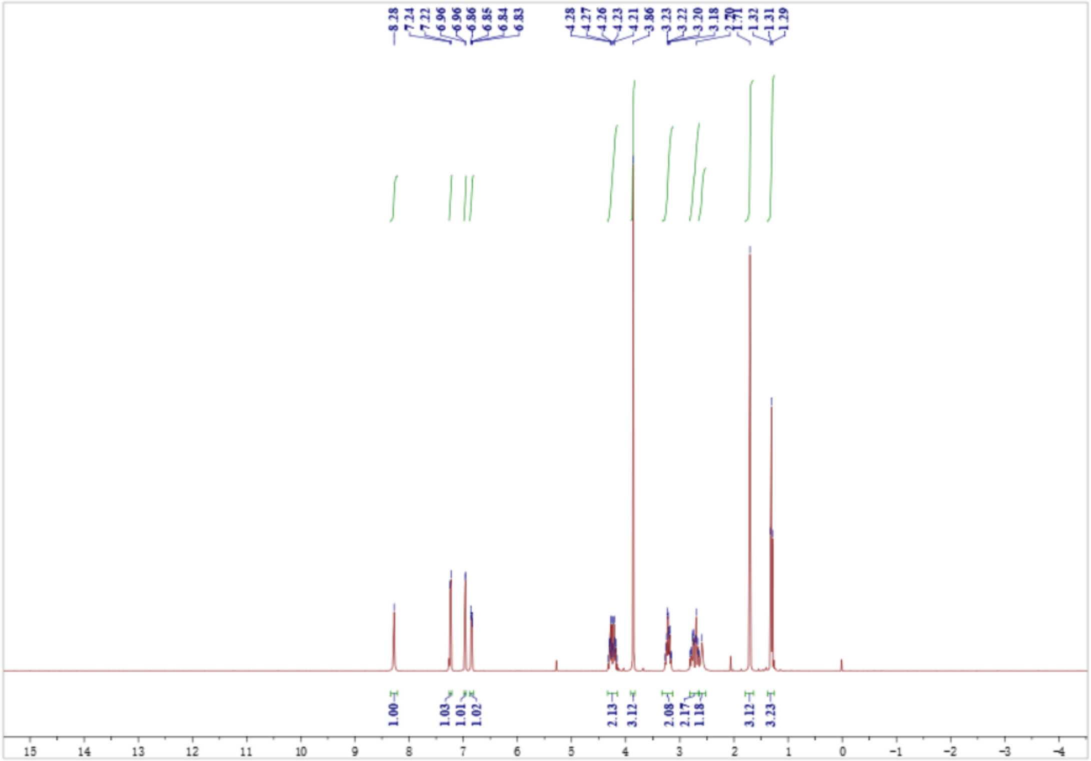

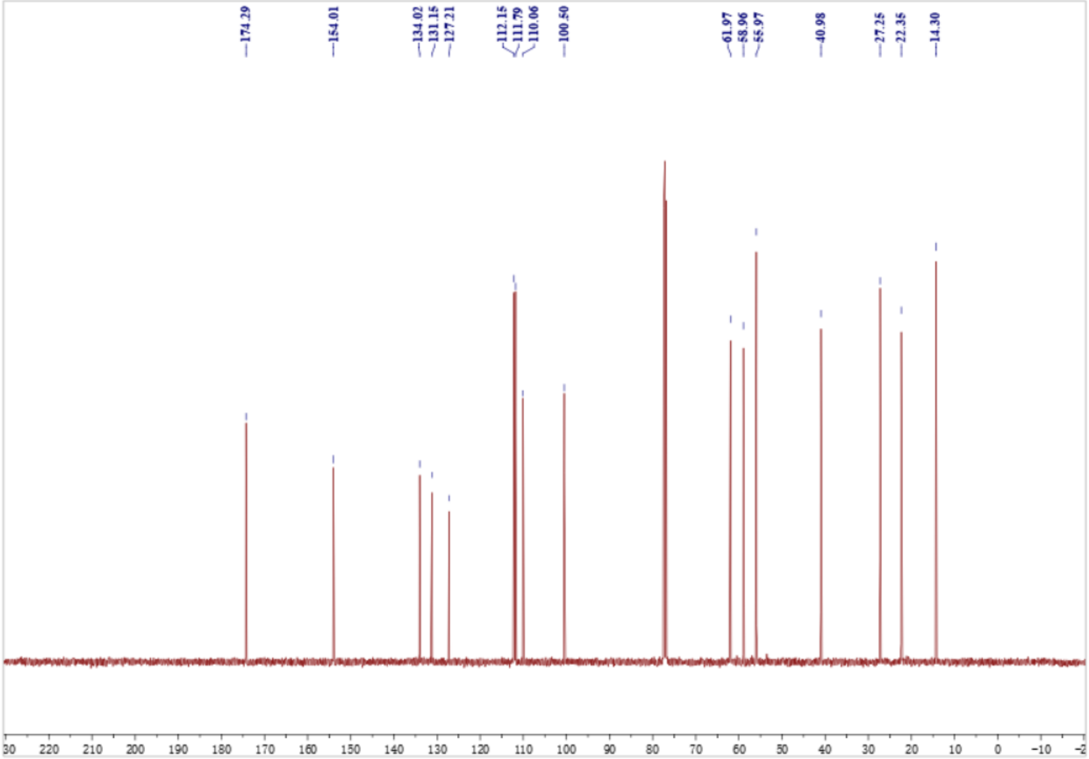

Ethyl(S)-2-(2-chloroacetyl)-1-methyl-2,3,4,9-tetrahydro-1H-pyrido[3,4-b]indole-1-carboxylate (**4**):

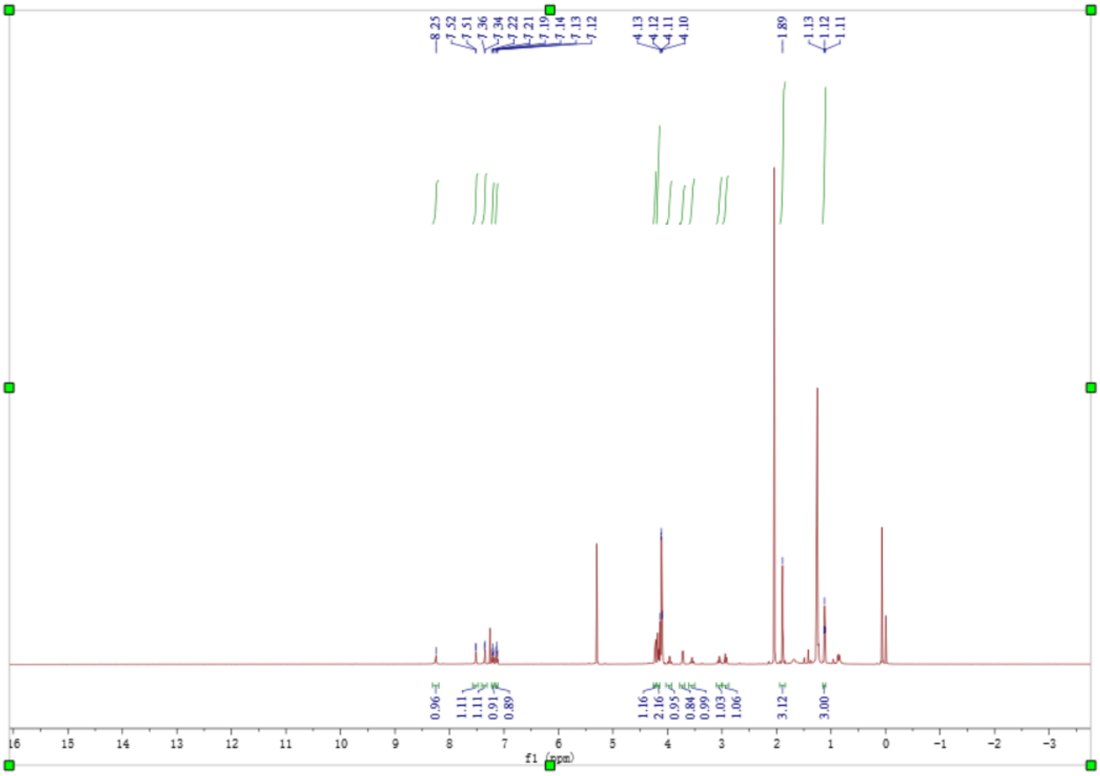

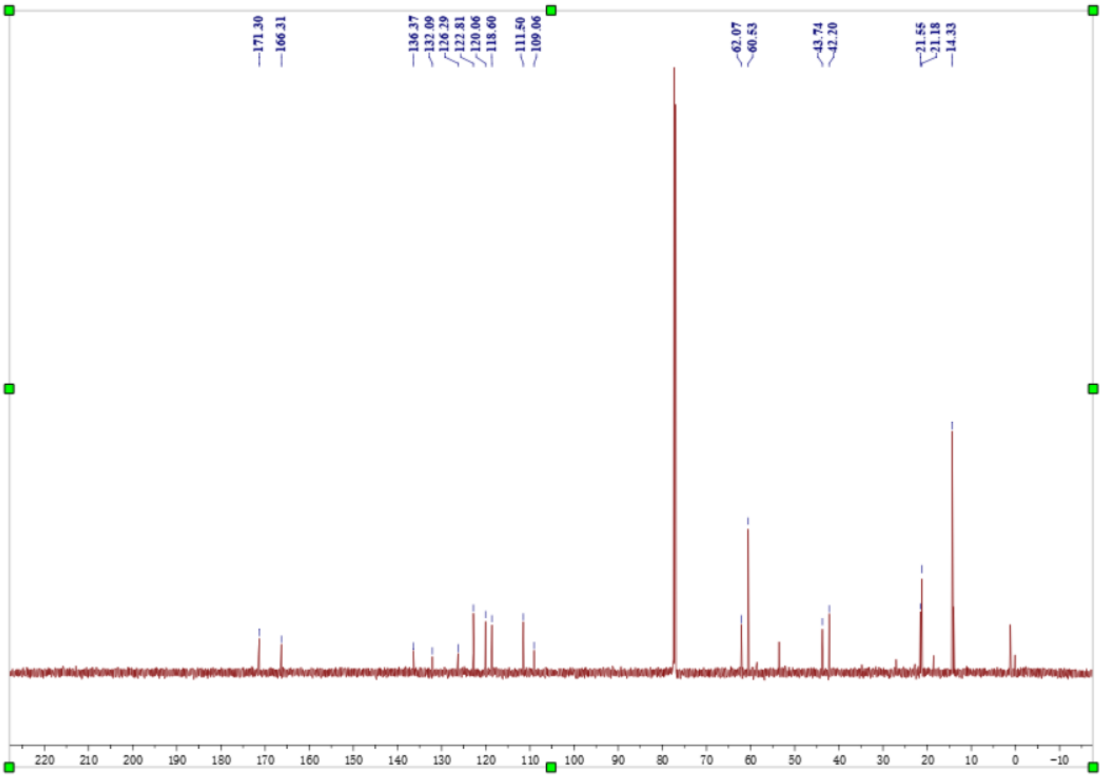

Ethyl(S)-2-(4-bromobenzoyl)-1-methyl-2,3,4,9-tetrahydro-1H-pyrido[3,4-b]indole-1-carboxylate (**5**):

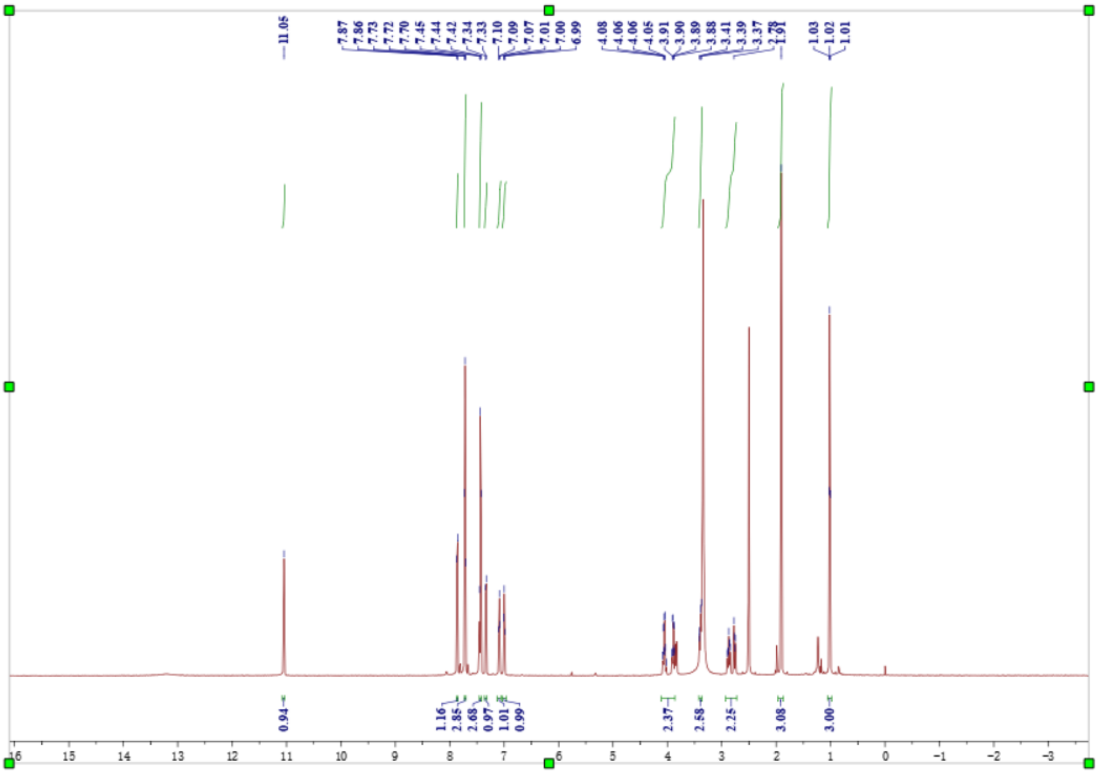

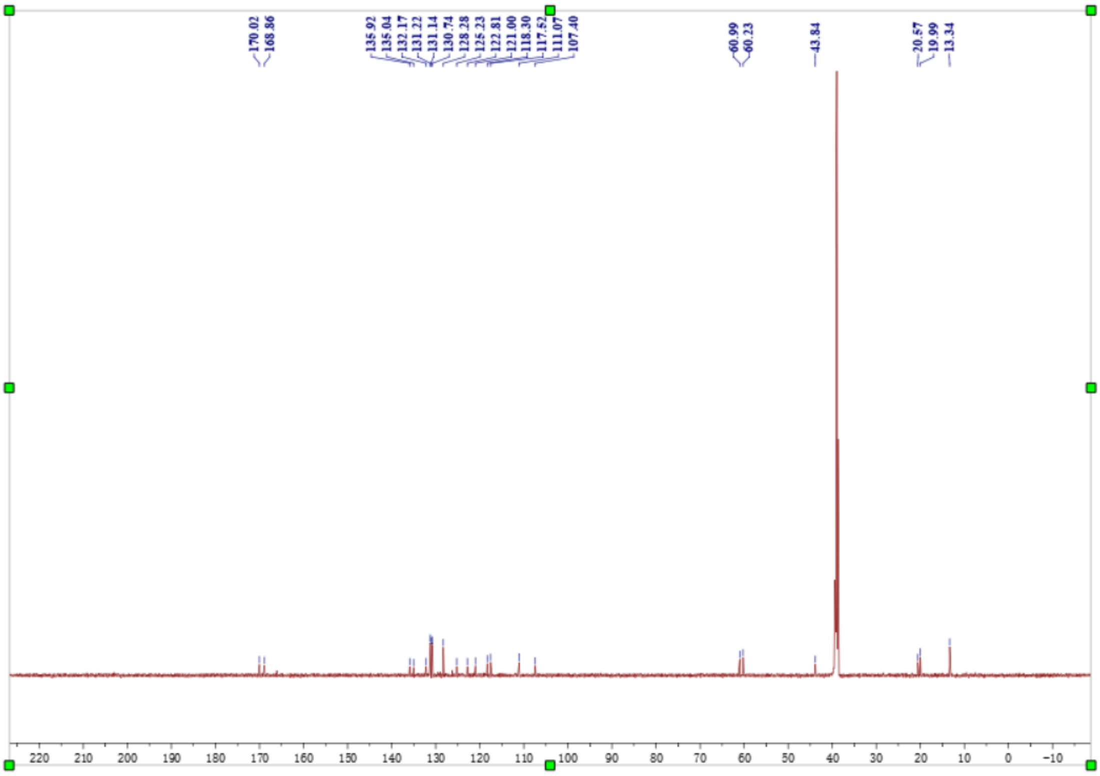

Ethyl(S)-2-(4-iodobenzoyl)-1-methyl-2,3,4,9-tetrahydro-1H-pyrido[3,4-b]indole-1-carboxylate (**6**):

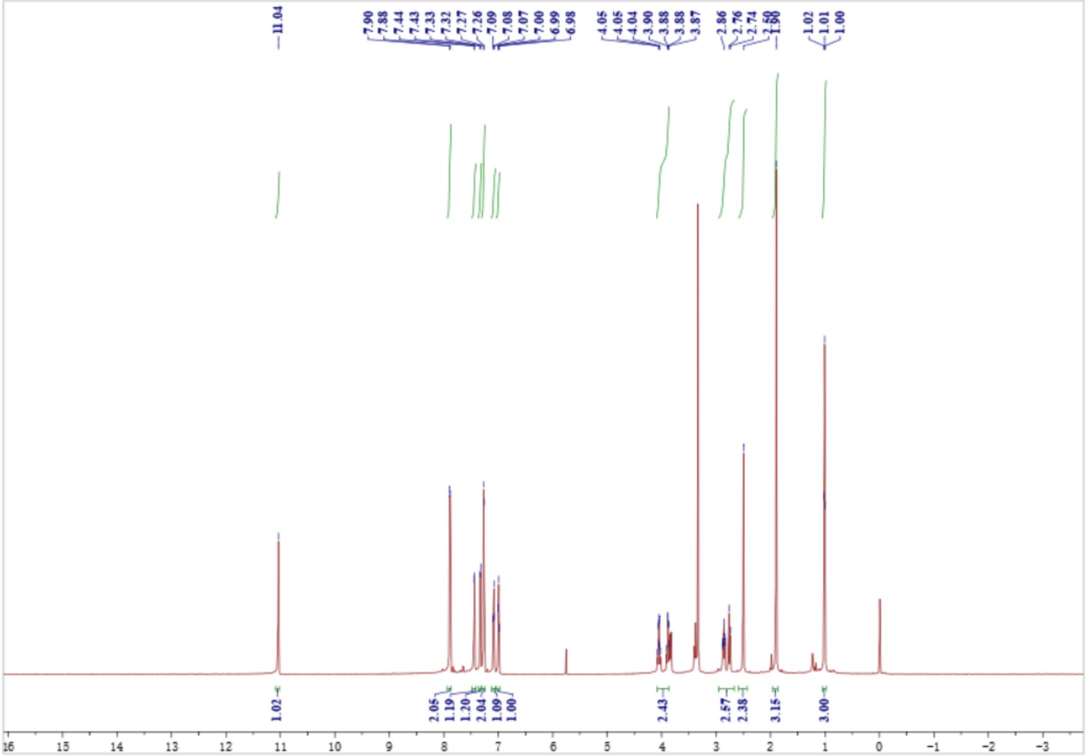

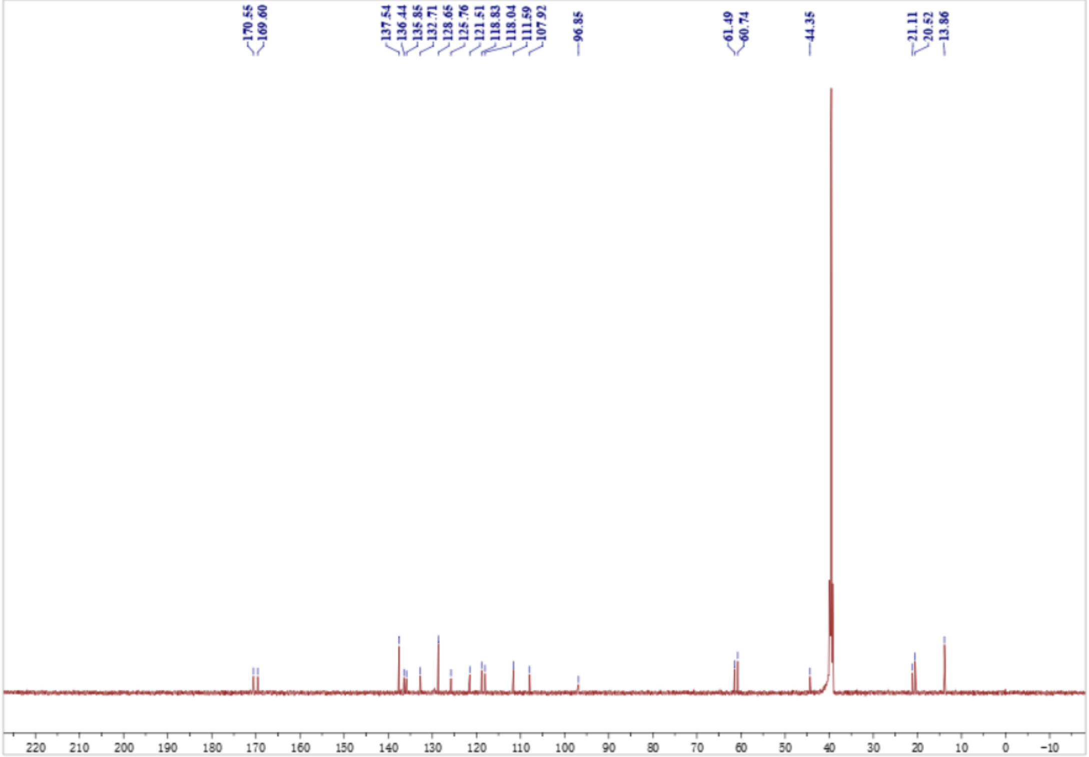

Ethyl(S)-1-methyl-2-(thiophen-2-ylsulfonyl)-2,3,4,9-tetrahydro-1H-pyrido[3,4-b]indole-1-carboxylate (**7**):

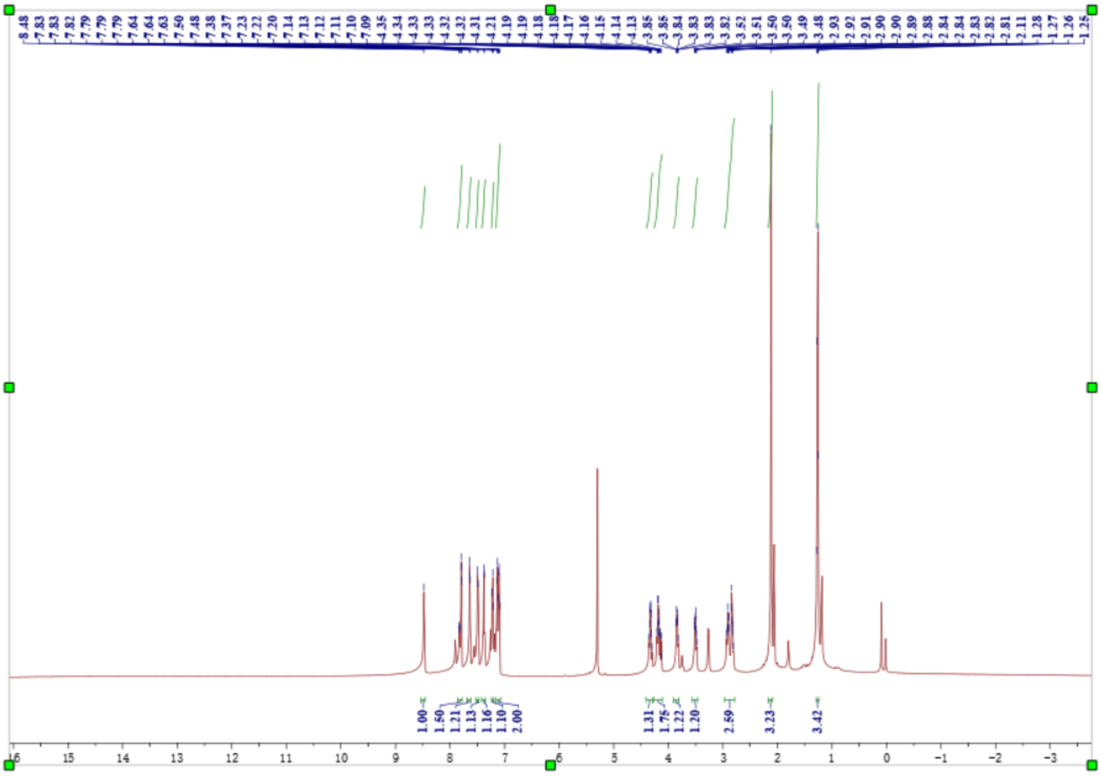

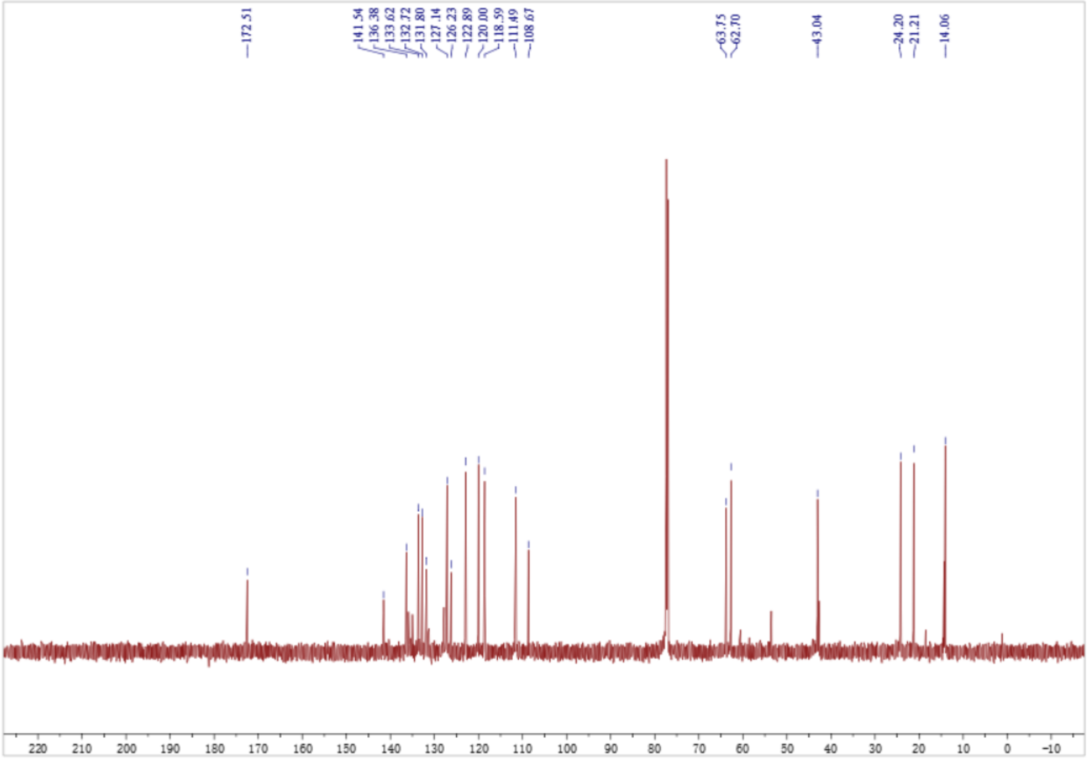

Ethyl(S)-1-methyl-2-undecanoyl-2,3,4,9-tetrahydro-1H-pyrido[3,4-b]indole-1-carboxylate (**8**):

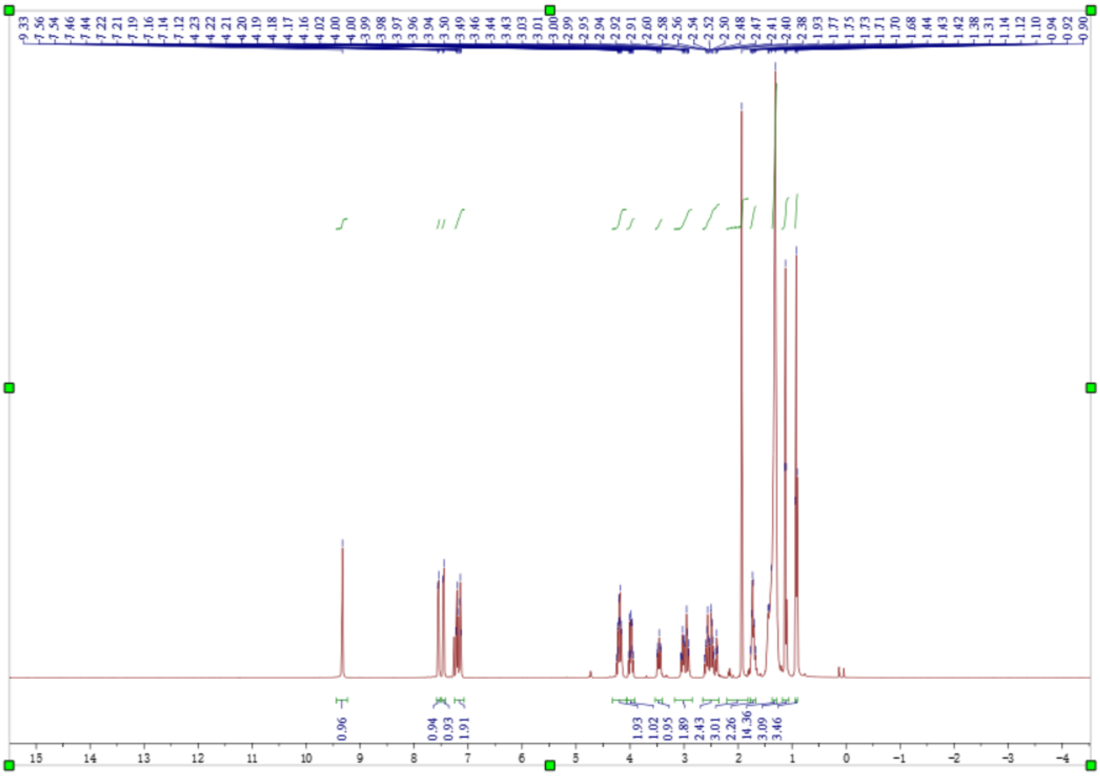

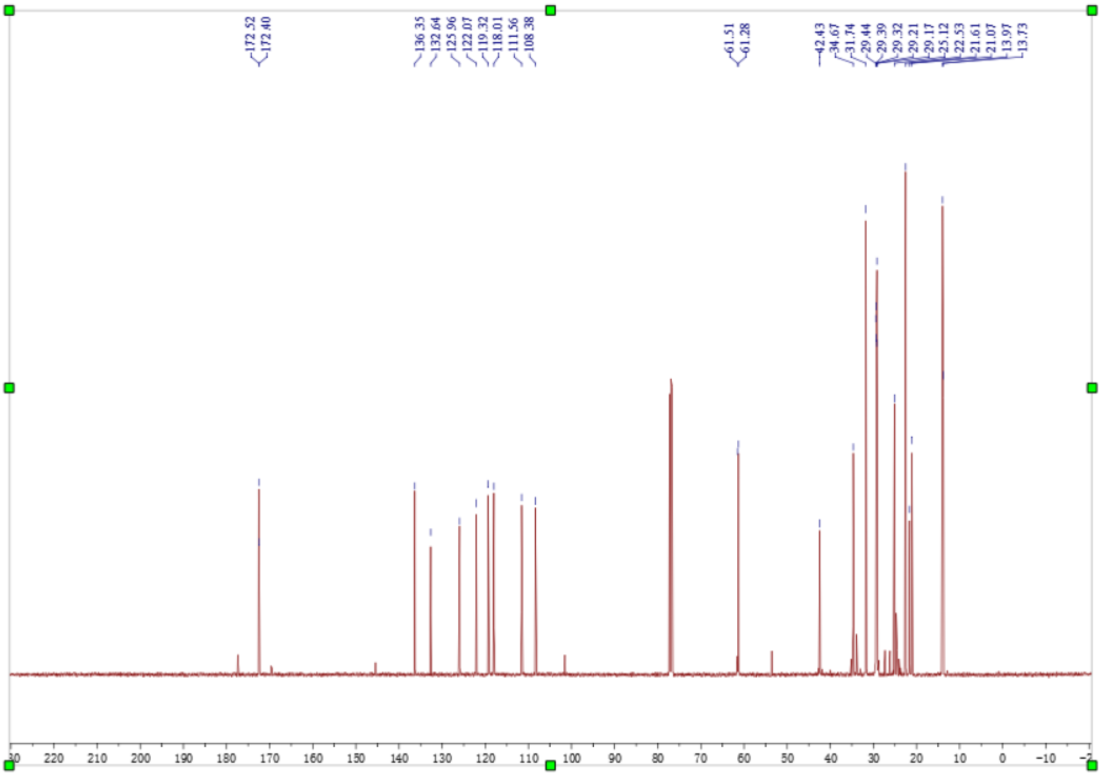

Ethyl(S)-2-butyryl-1-methyl-2,3,4,9-tetrahydro-1H-pyrido[3,4-b]indole-1-carboxylate (**9**):

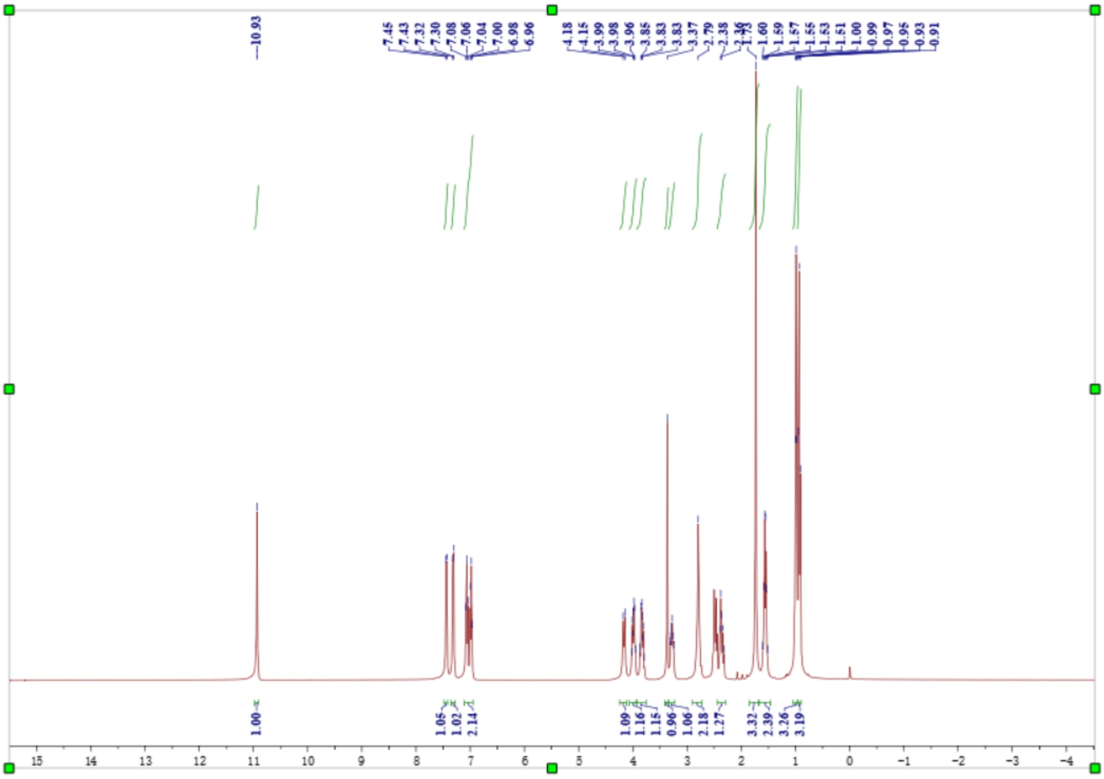

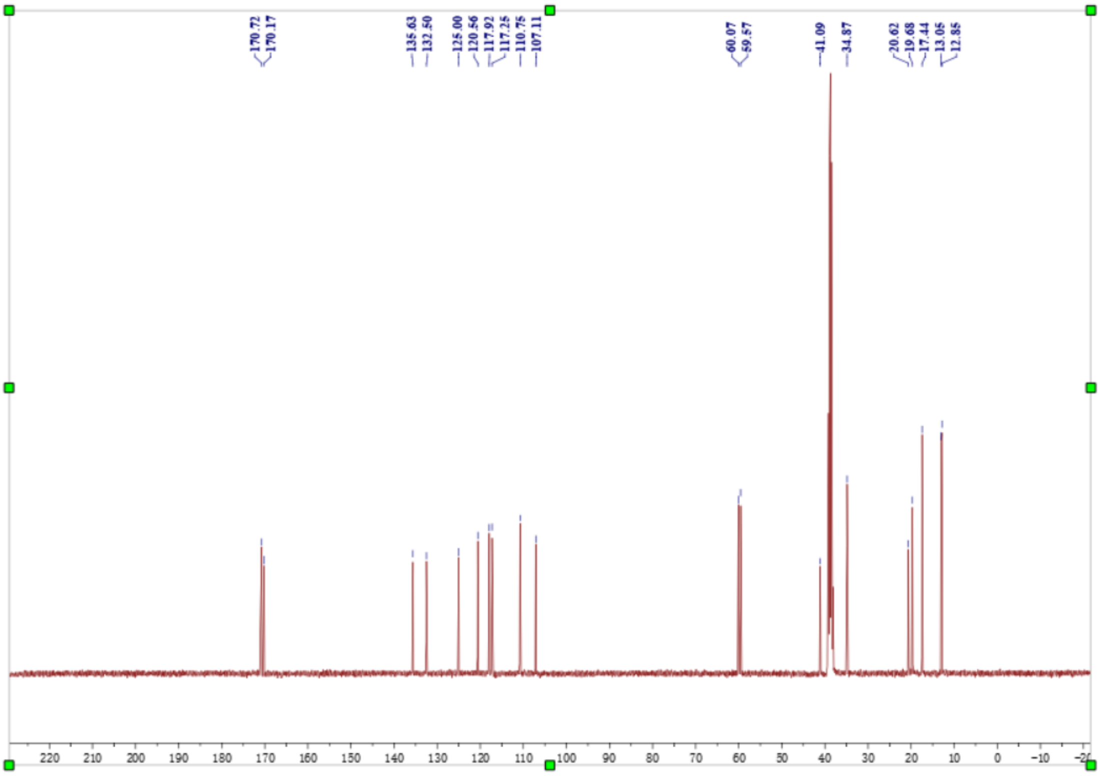

Ethyl(S)-1-methyl-2-propionyl-2,3,4,9-tetrahydro-1H-pyrido[3,4-b]indole-1-carboxylate (**10**):

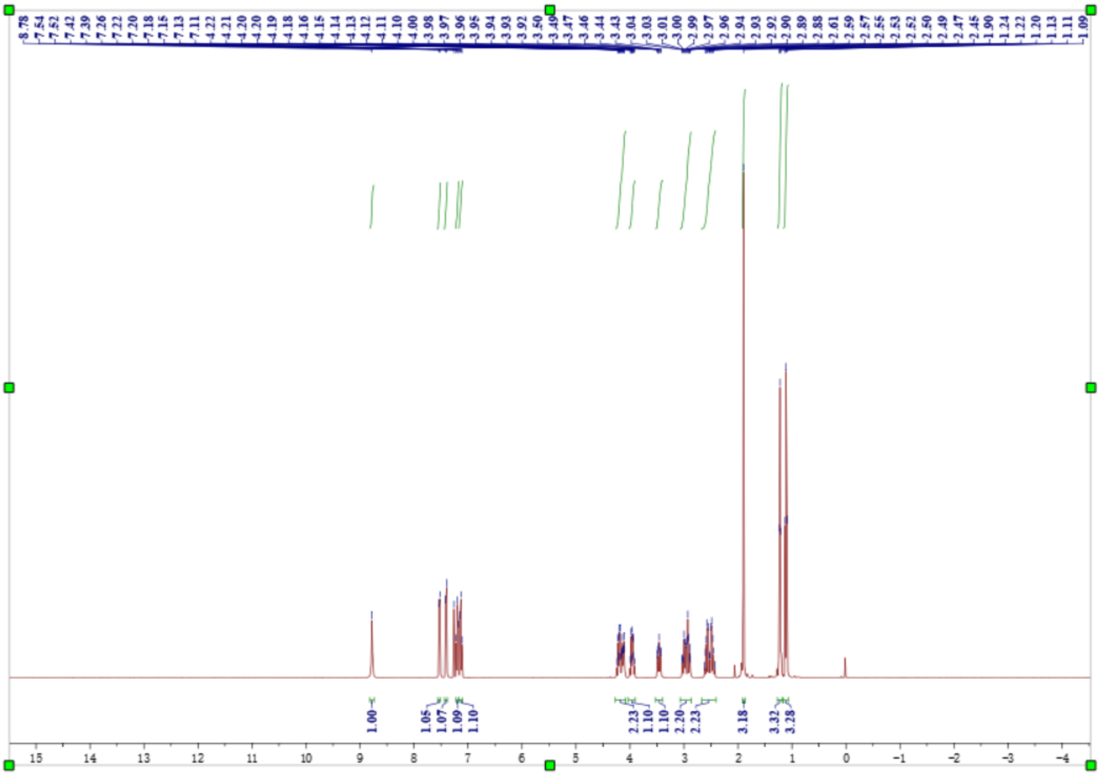

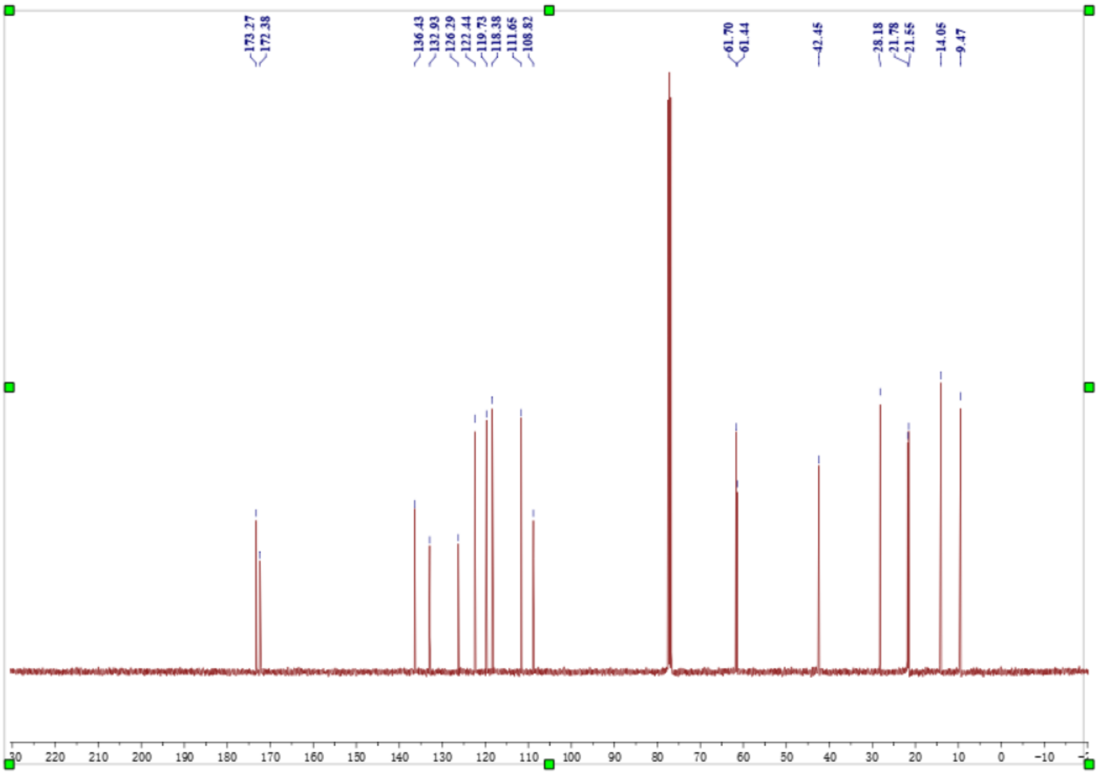

Ethyl(S)-2-dodecanoyl-1-methyl-2,3,4,9-tetrahydro-1H-pyrido[3,4-b]indole-1-carboxylate (**11**):

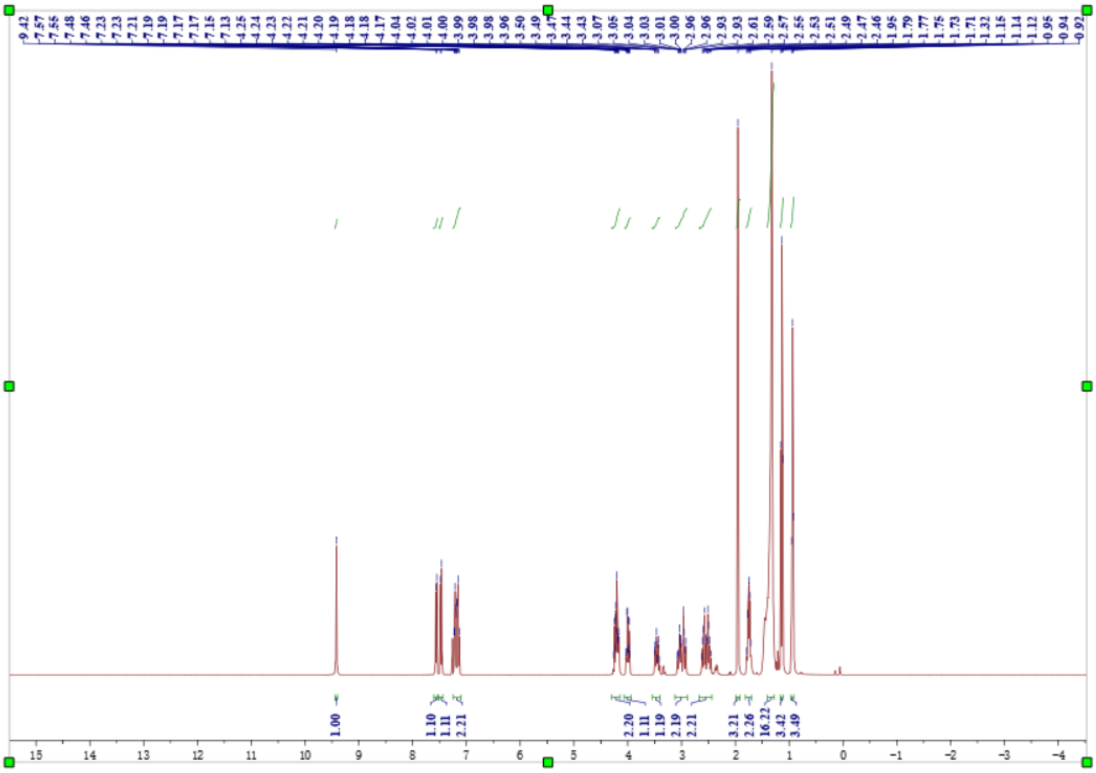

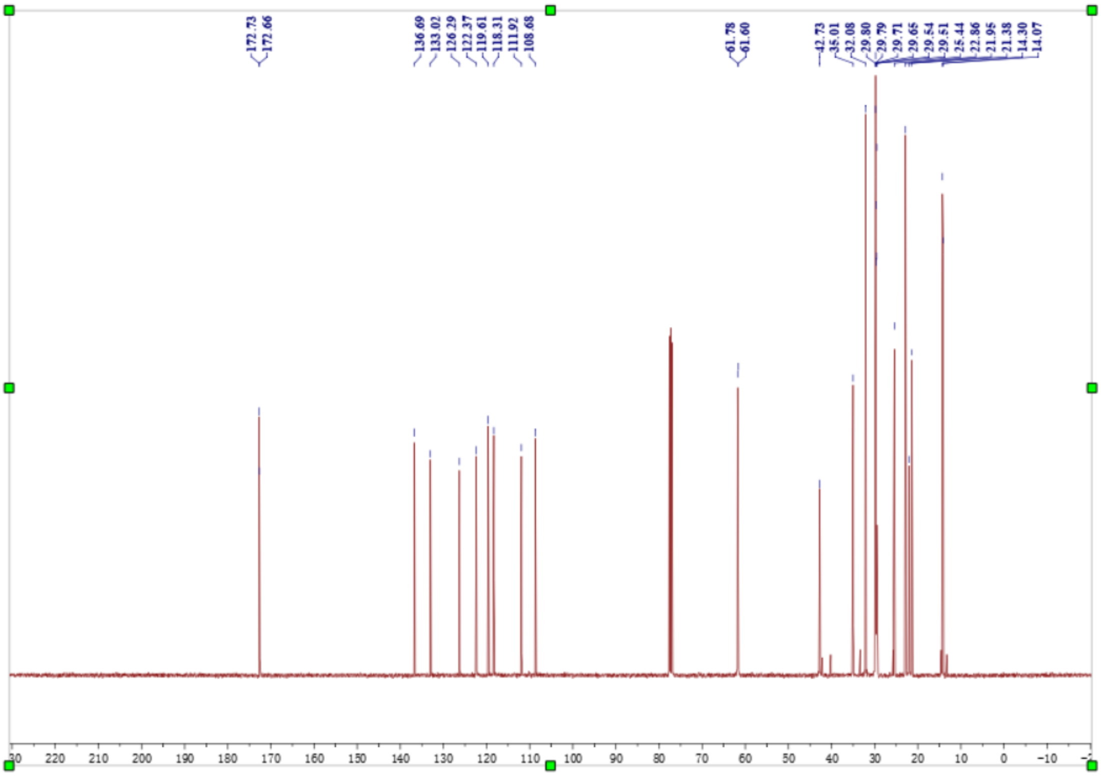

Ethyl(S)-2-(2-chloroacetyl)-6-methoxy-1-methyl-2,3,4,9-tetrahydro-1H-pyrido[3,4-b]indole-1-carboxylate (**12**):

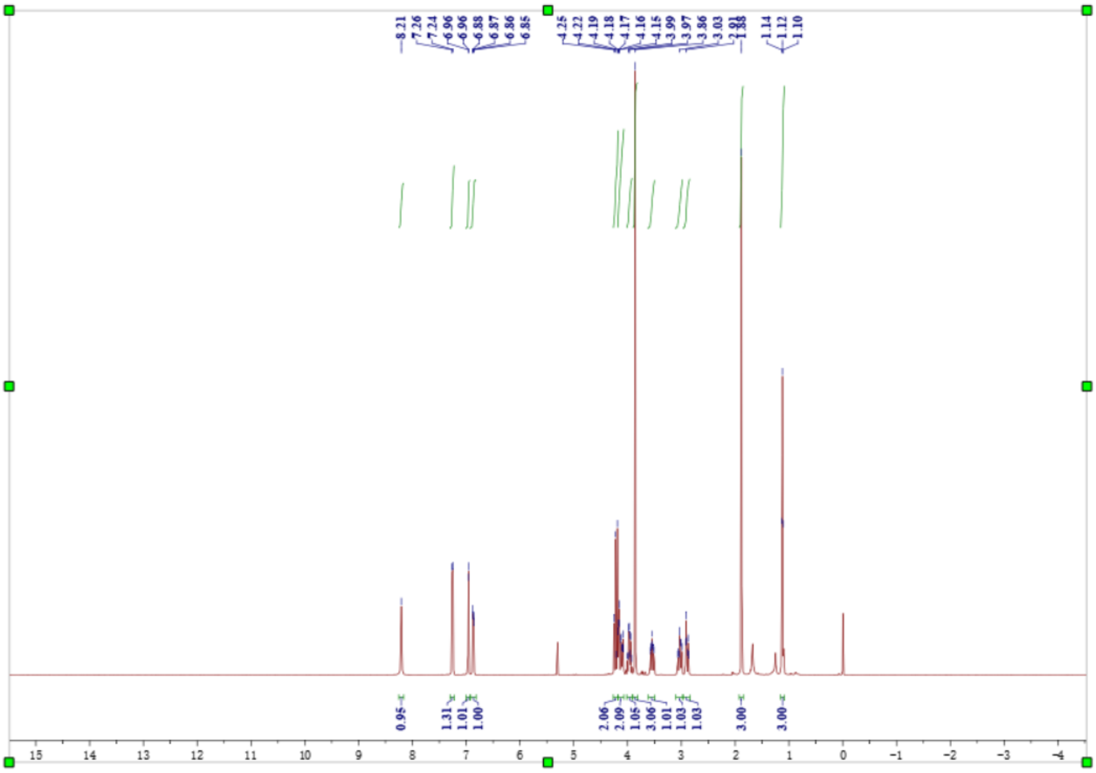

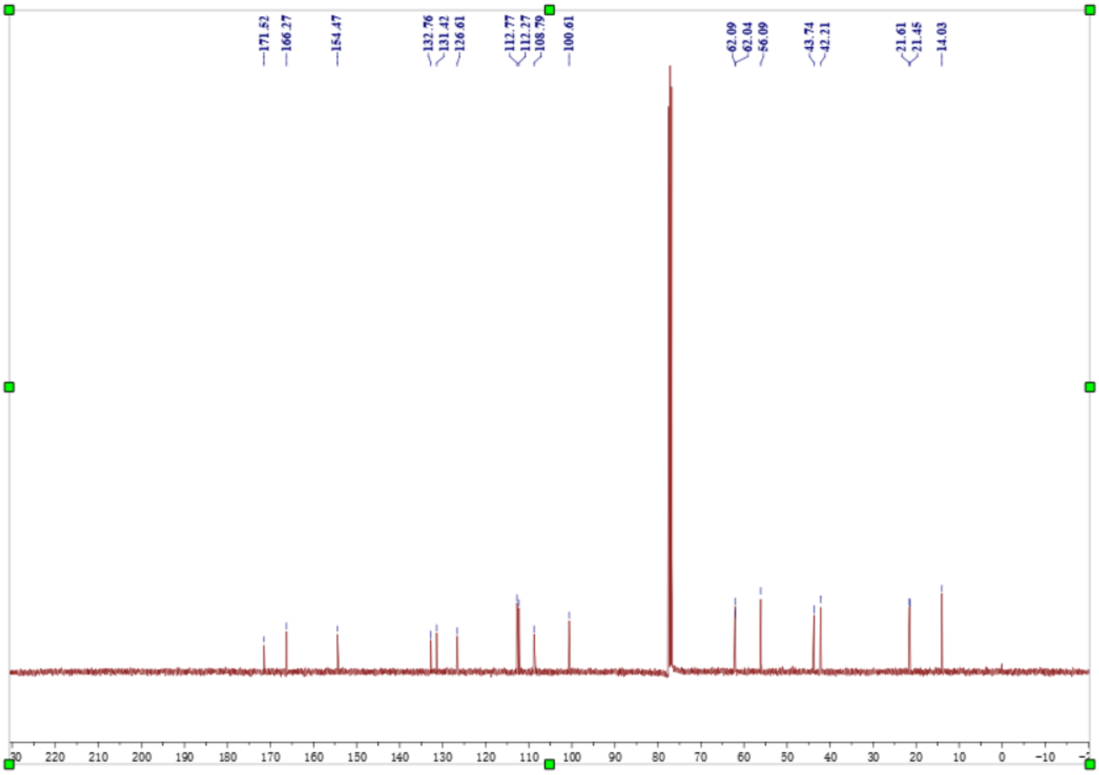

Ethyl(S)-2-(4-bromobenzoyl)-6-methoxy-1-methyl-2,3,4,9-tetrahydro-1H-pyrido[3,4-b]indole-1-carboxylate (**13**):

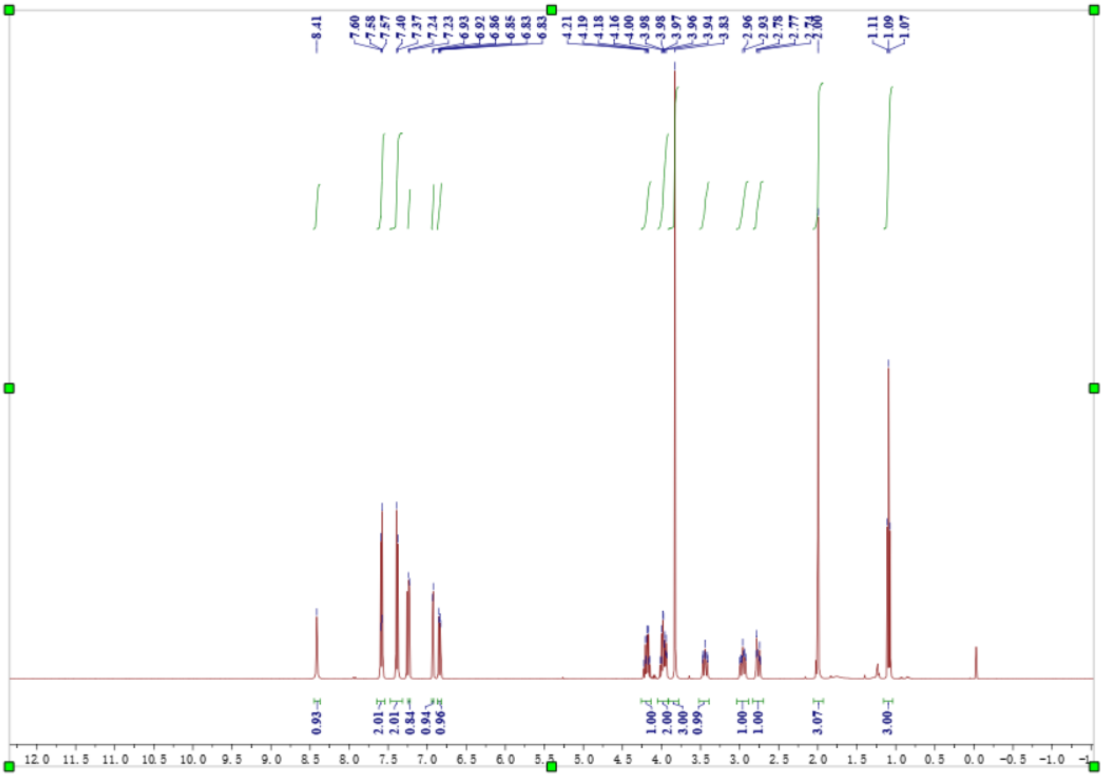

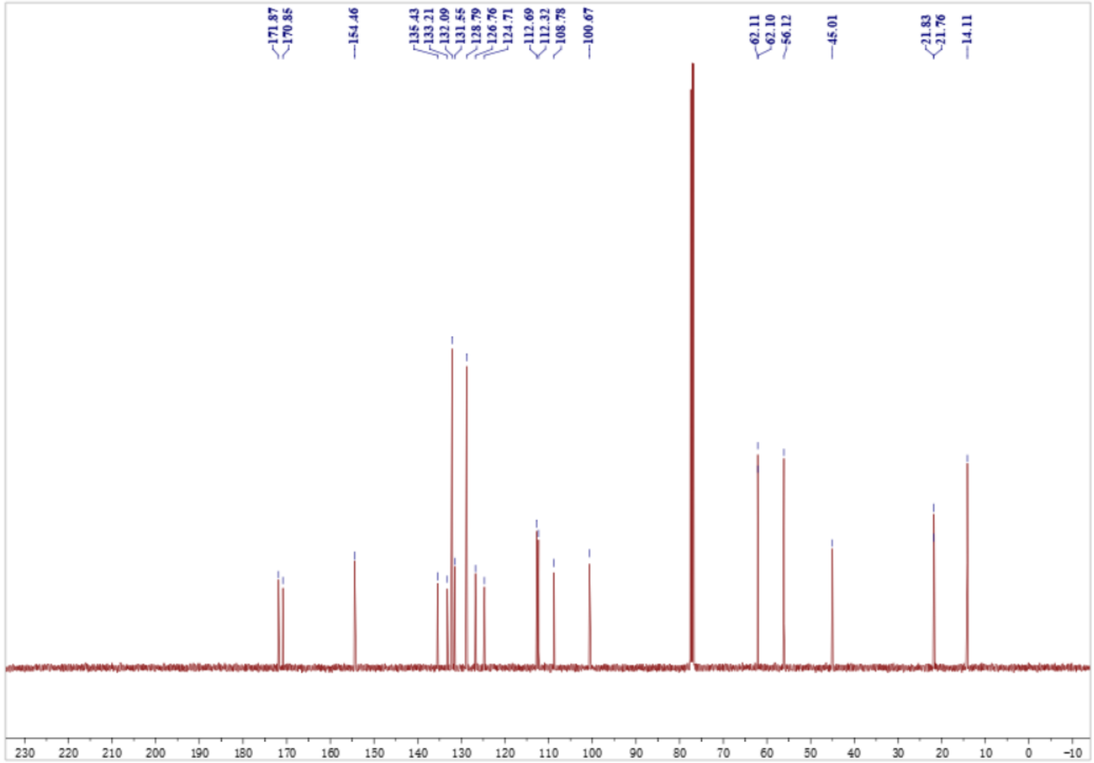

Ethyl(S)-2-(4-iodobenzoyl)-6-methoxy-1-methyl-2,3,4,9-tetrahydro-1H-pyrido[3,4-b]indole-1-carboxylate (**14**):

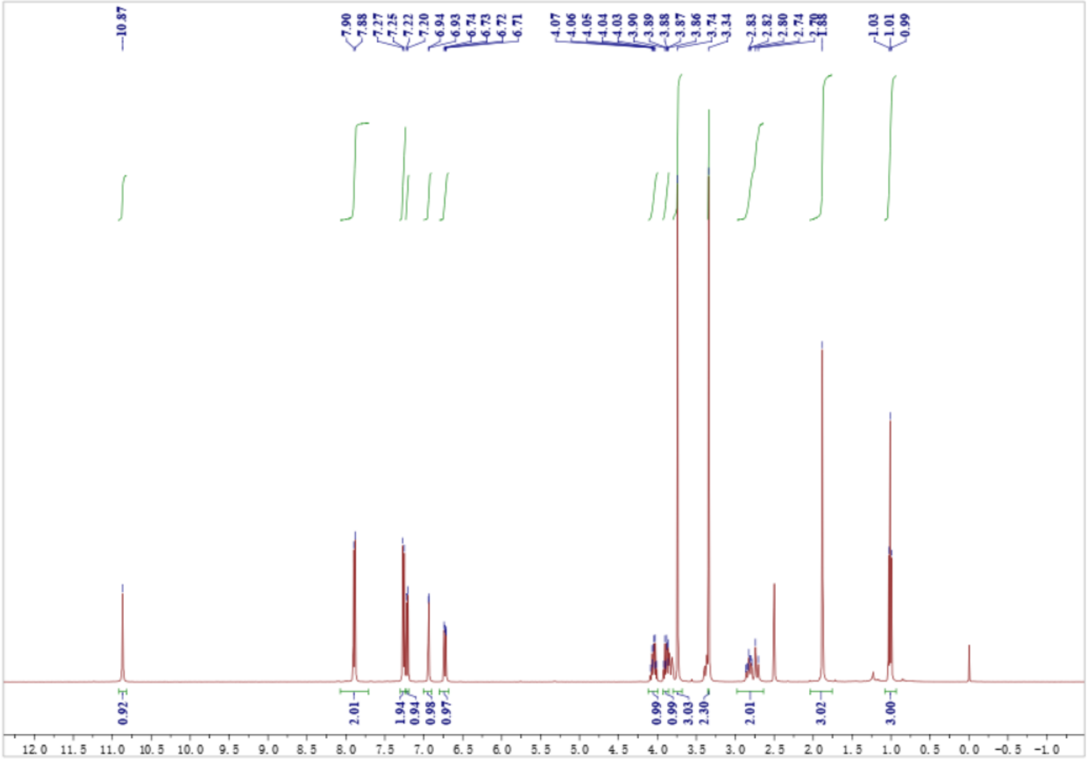

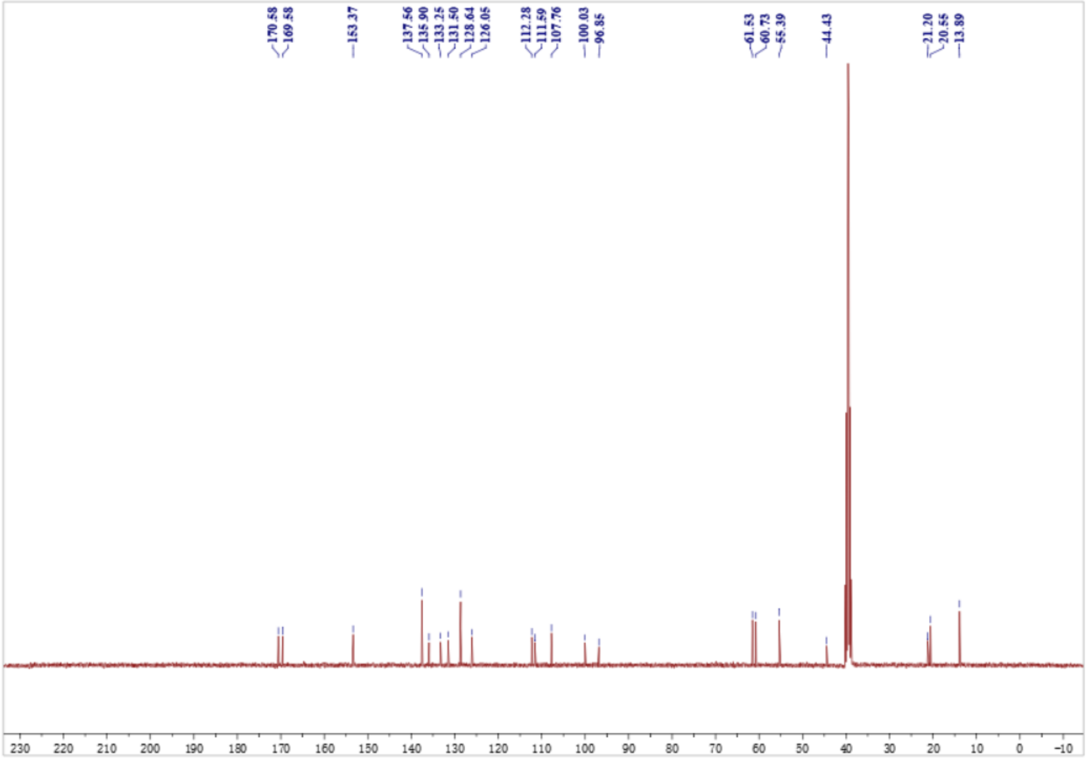

Ethyl(S)-6-methoxy-1-methyl-2-(thiophen-2-ylsulfonyl)-2,3,4,9-tetrahydro-1H-pyrido[3,4-b]indole-1-carboxylate (**15**):

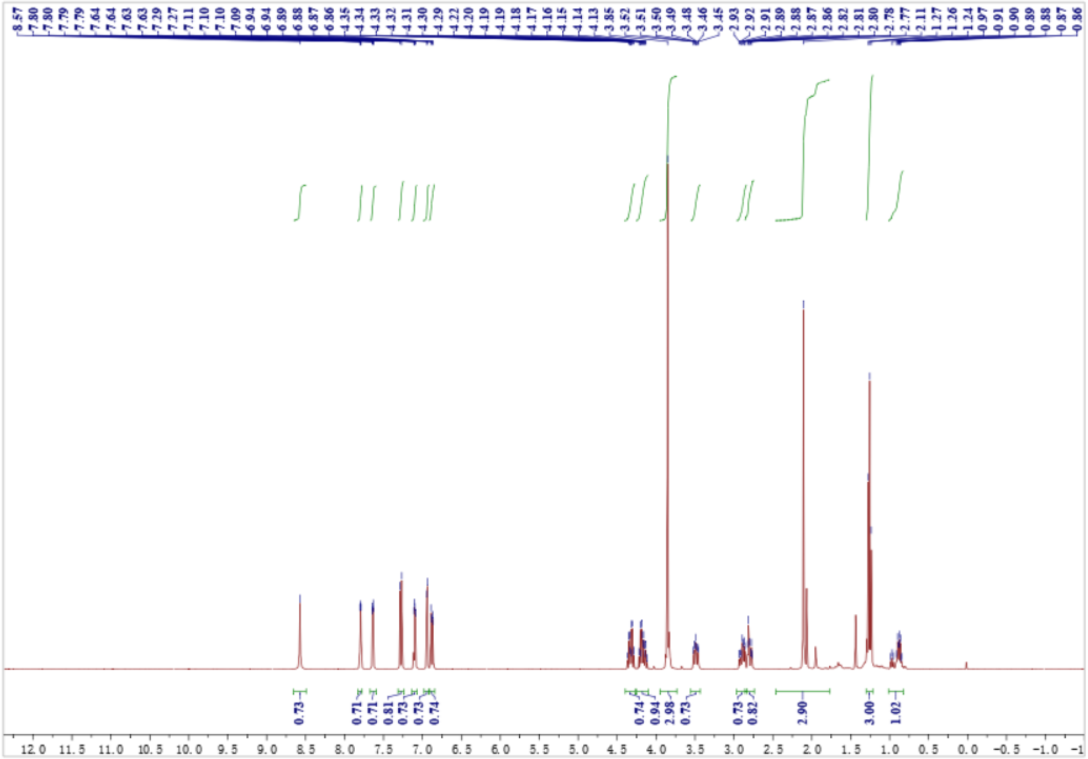

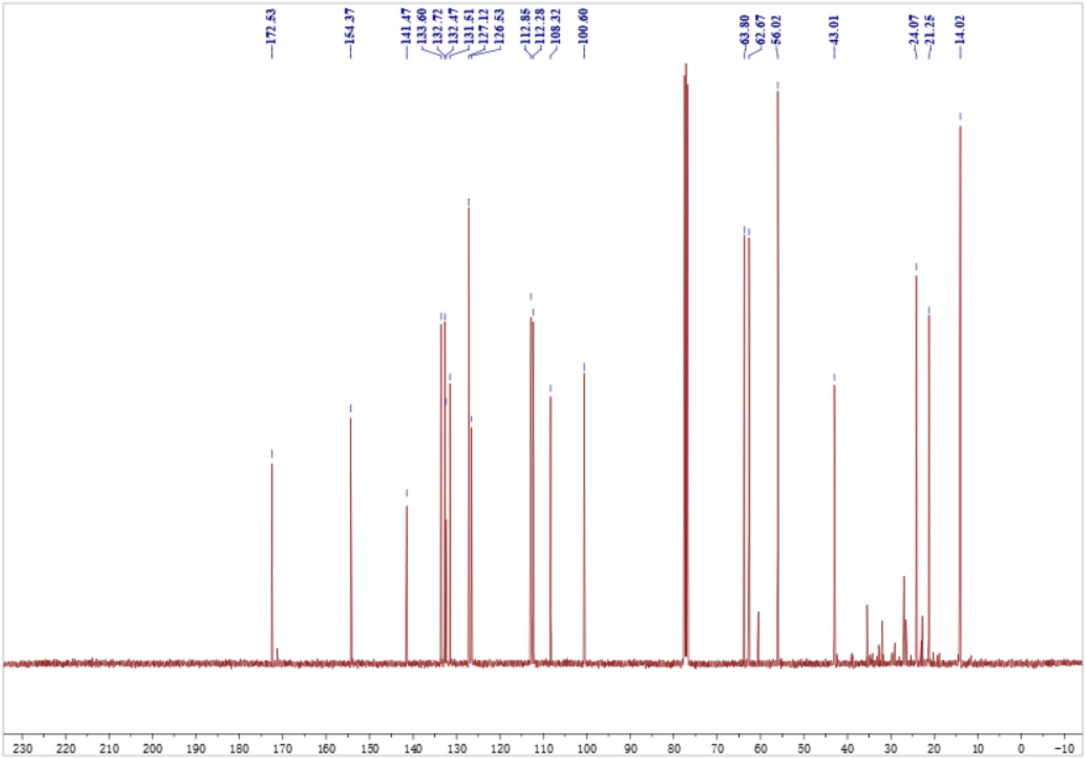

Ethyl(S)-6-methoxy-1-methyl-2-undecanoyl-2,3,4,9-tetrahydro-1H-pyrido[3,4-b]indole-1-carboxylate (**16**):

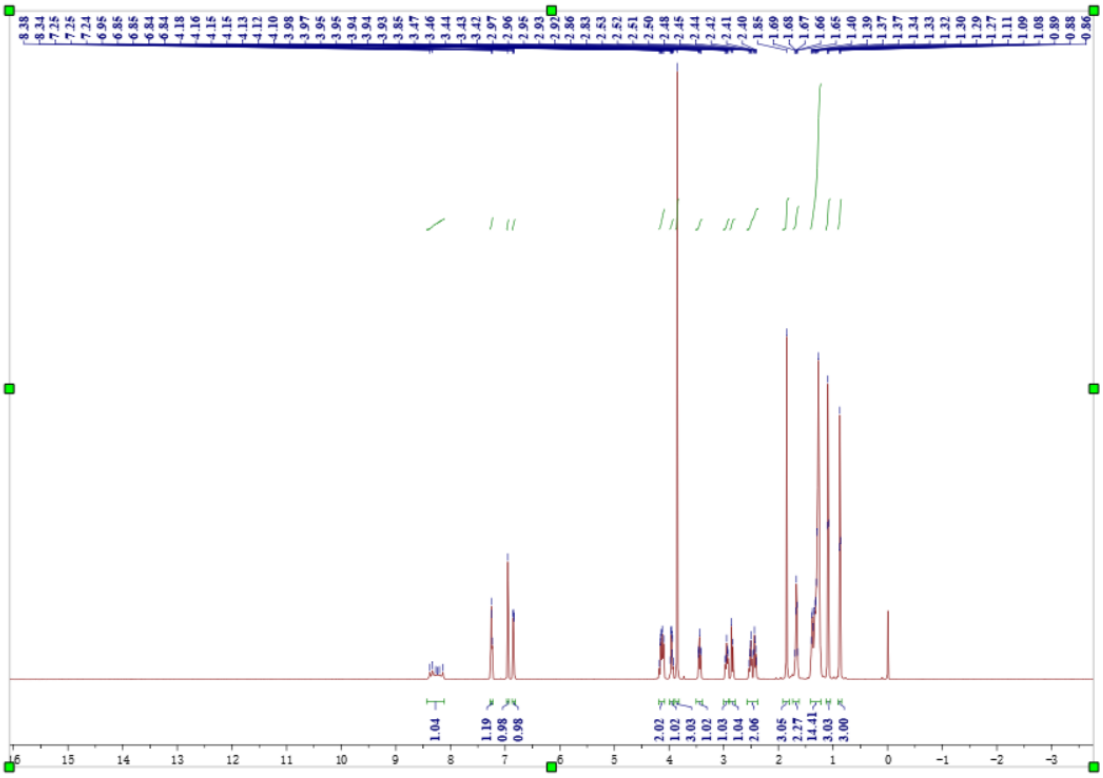

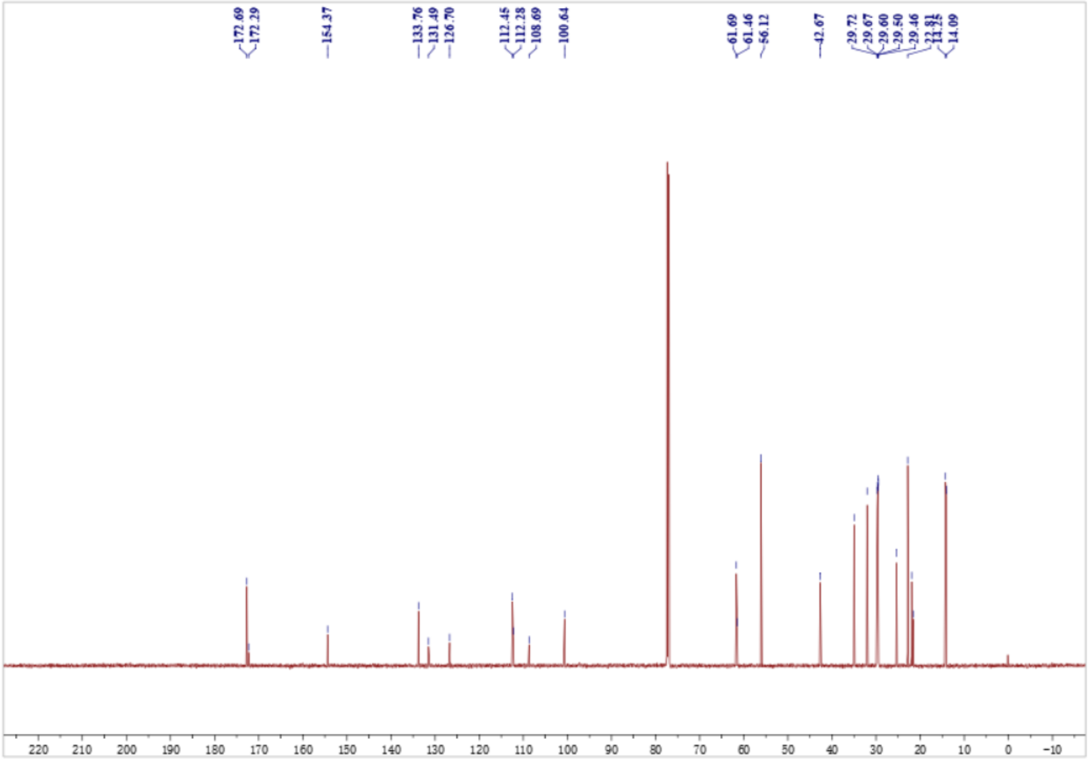

Ethyl(S)-2-butyryl-6-methoxy-1-methyl-2,3,4,9-tetrahydro-1H-pyrido[3,4-b]indole-1-carboxylate (**17**):

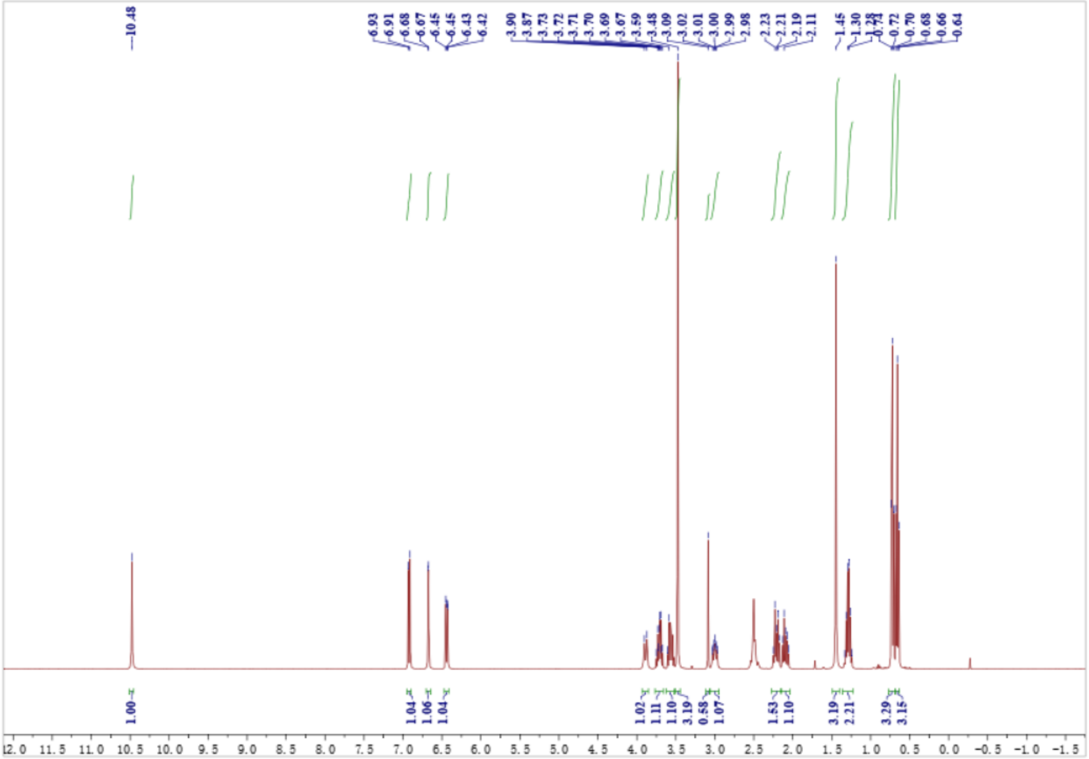

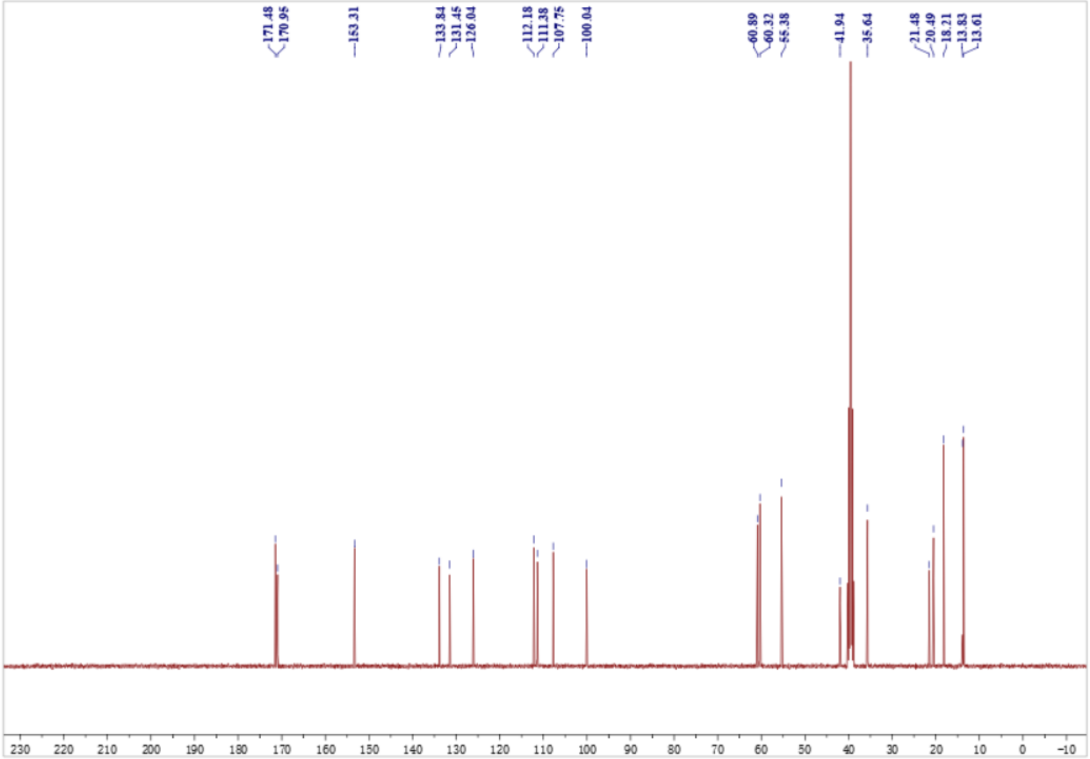

Ethyl(S)-6-methoxy-1-methyl-2-propionyl-2,3,4,9-tetrahydro-1H-pyrido[3,4-b]indole-1-carboxylate (**18**):

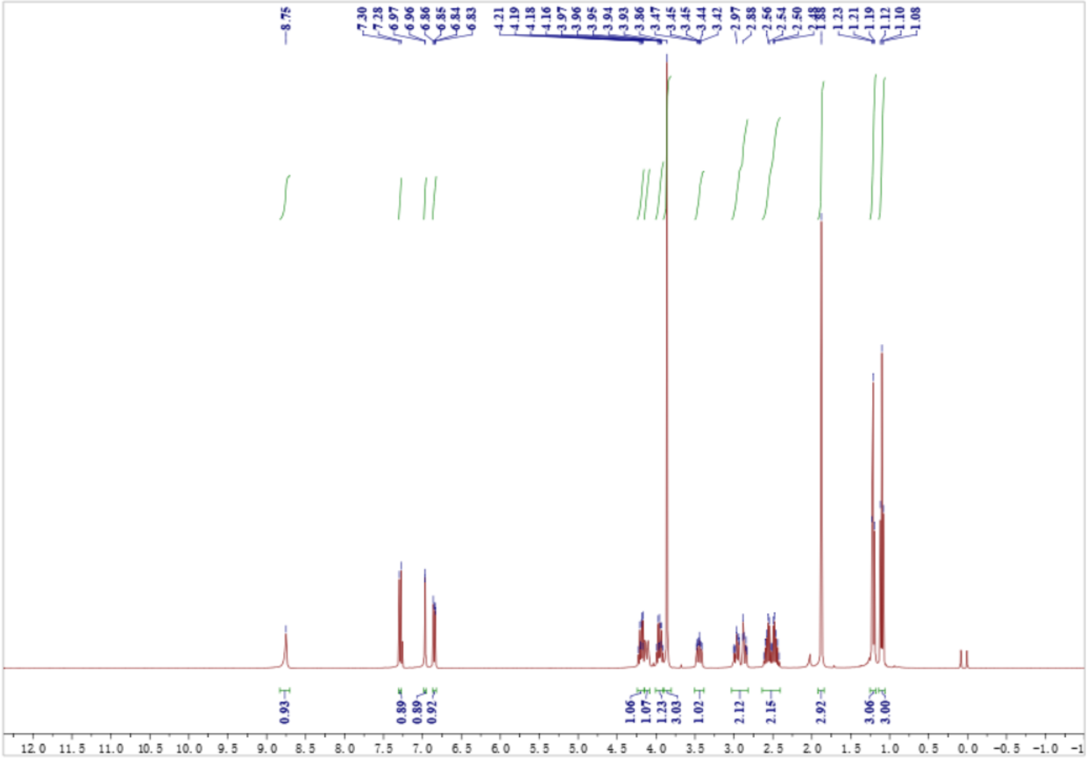

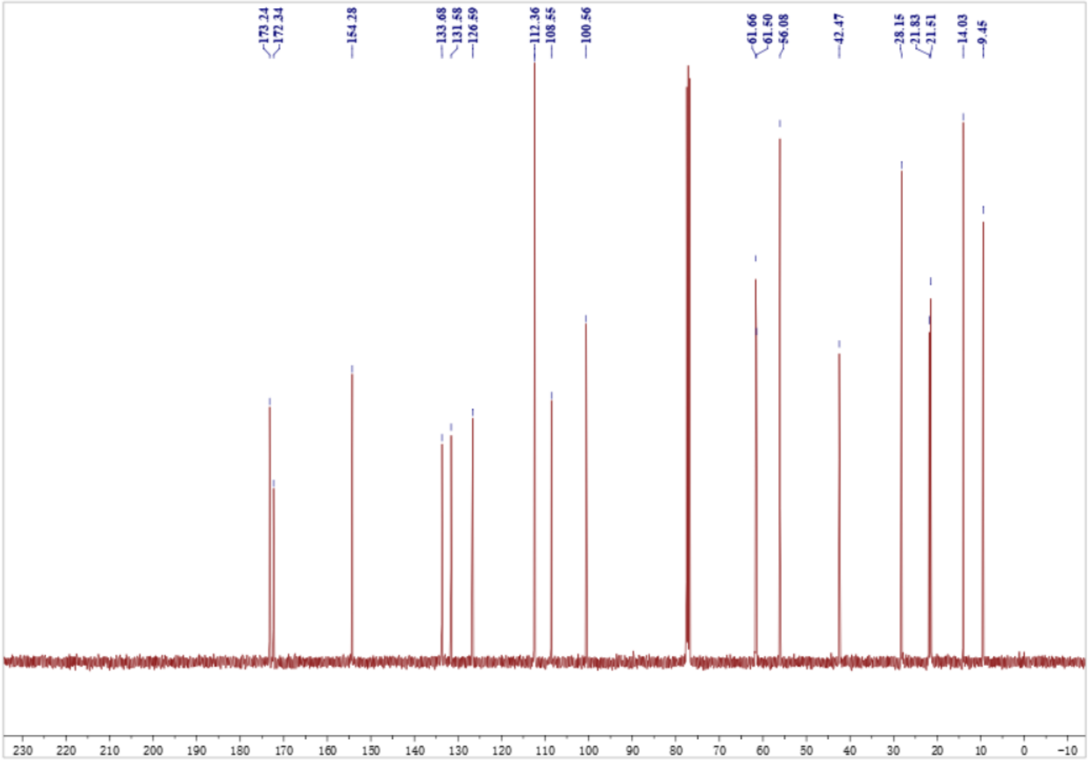

Ethyl(S)-2-dodecanoyl-6-methoxy-1-methyl-2,3,4,9-tetrahydro-1H-pyrido[3,4-b]indole-1-carboxylate (**19**):

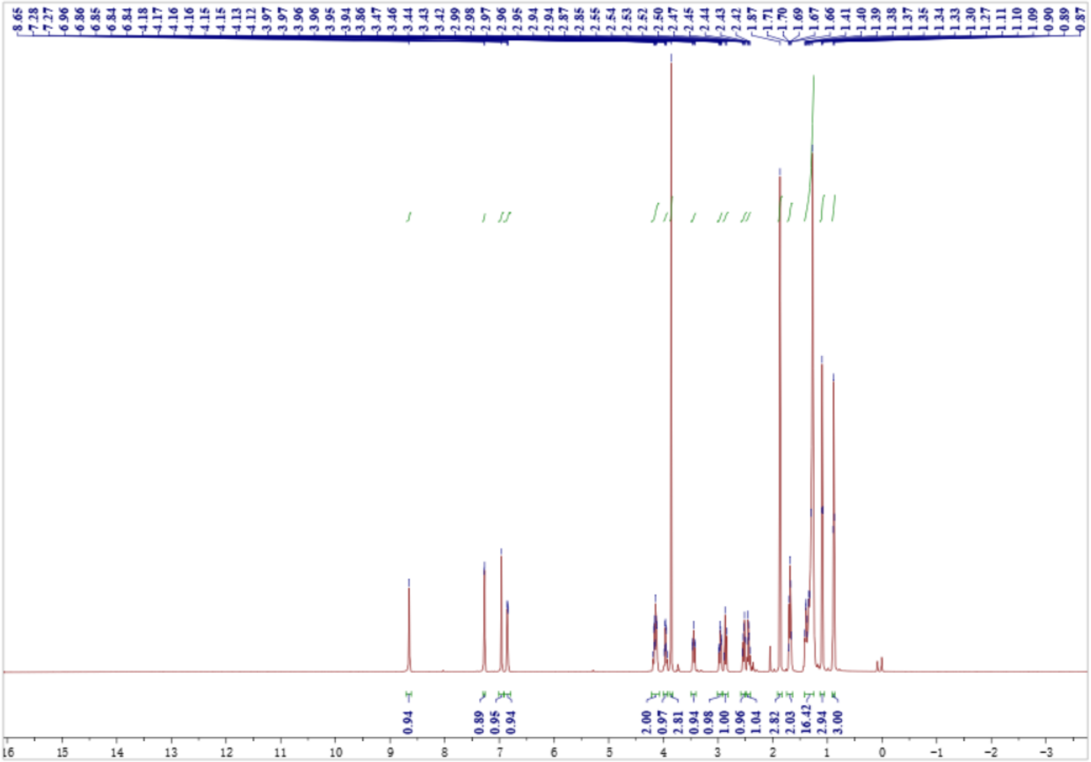

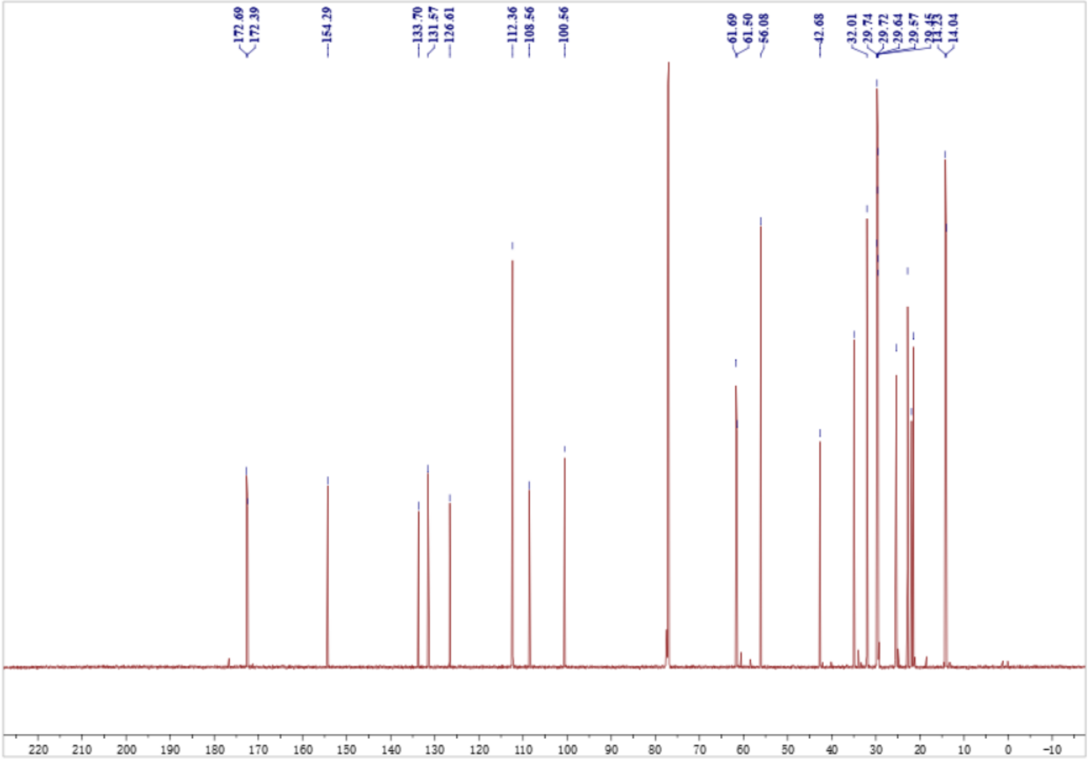

Ethyl(S,E)-2-(3-(4-chlorophenyl)acryloyl)-1-methyl-2,3,4,9-tetrahydro-1H-pyrido[3,4-b]indole-1-carboxylate (**20**):

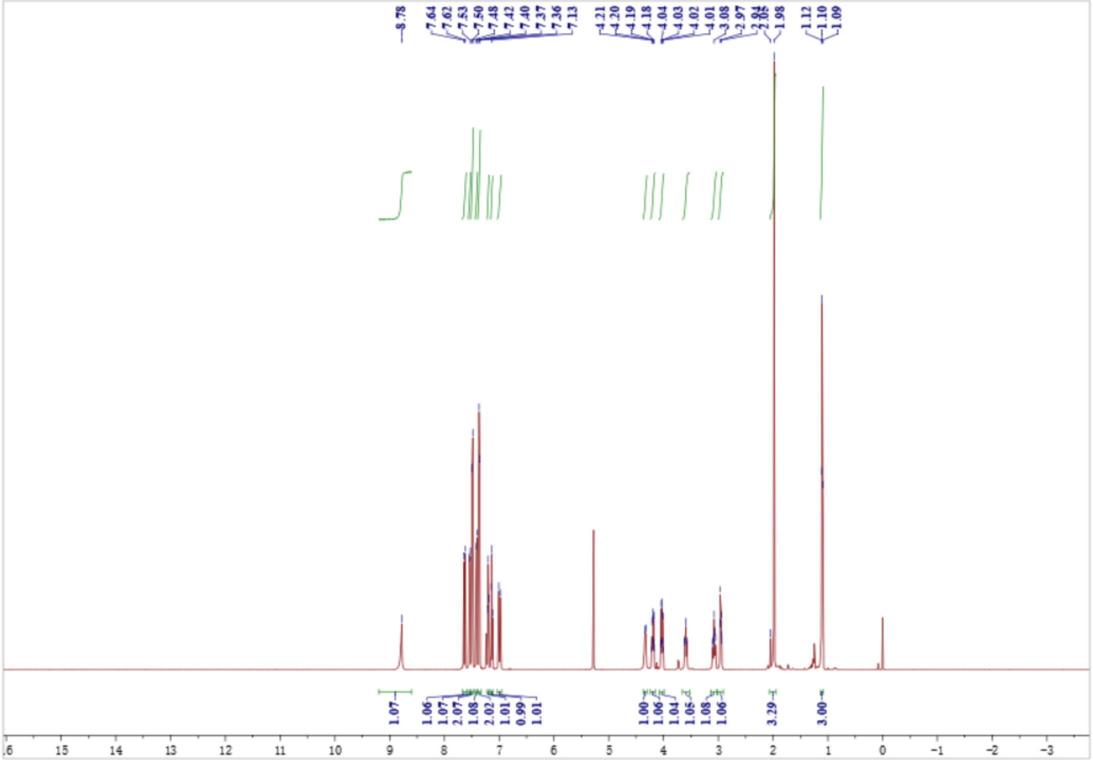

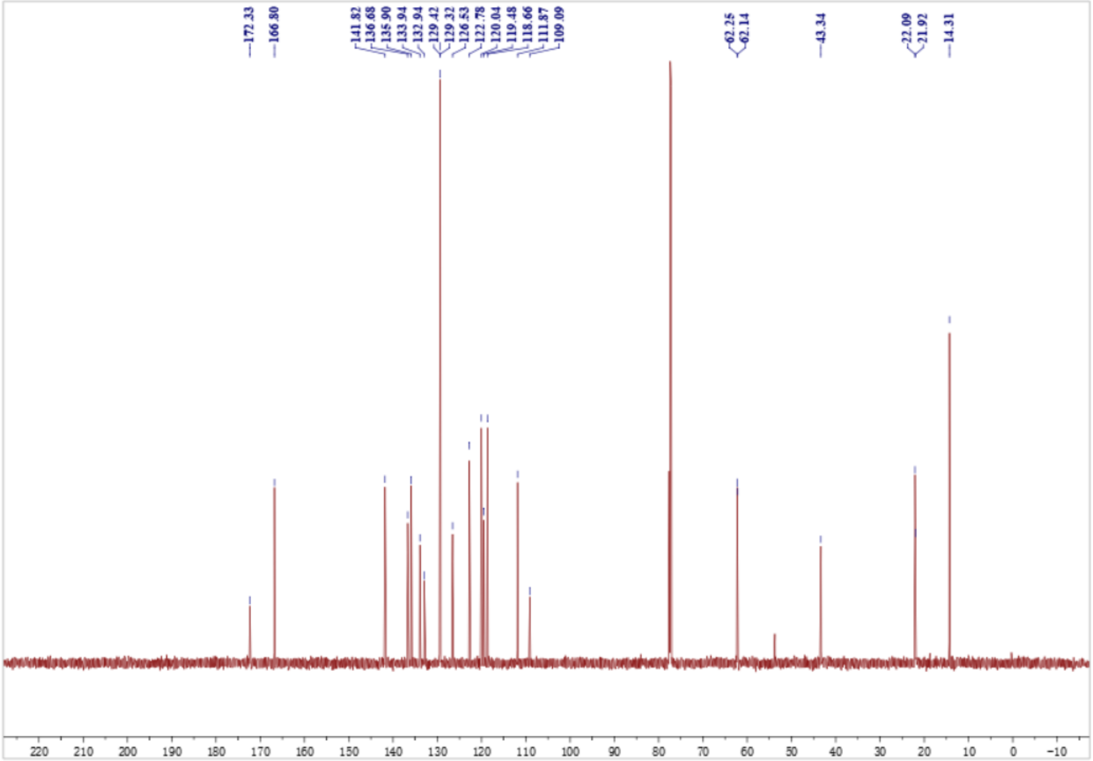

Ethyl(S,E)-1-methyl-2-(3-(p-tolyl)acryloyl)-2,3,4,9-tetrahydro-1H-pyrido[3,4-b]indole-1-carboxylate (**21**):

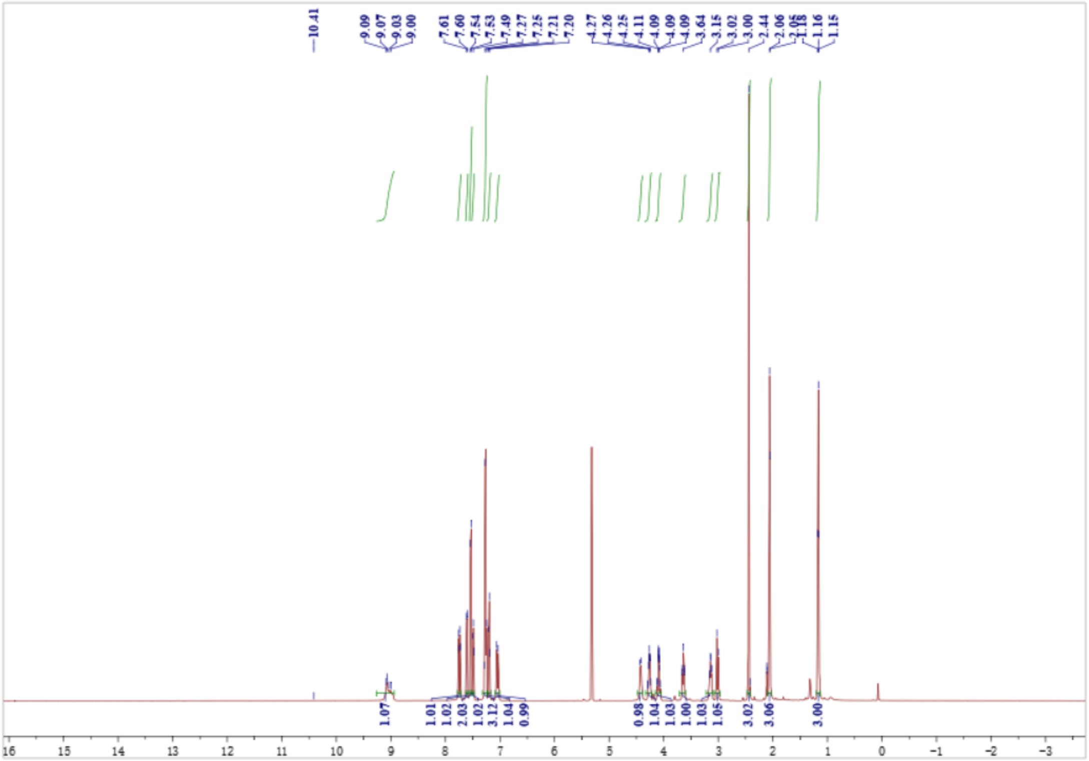

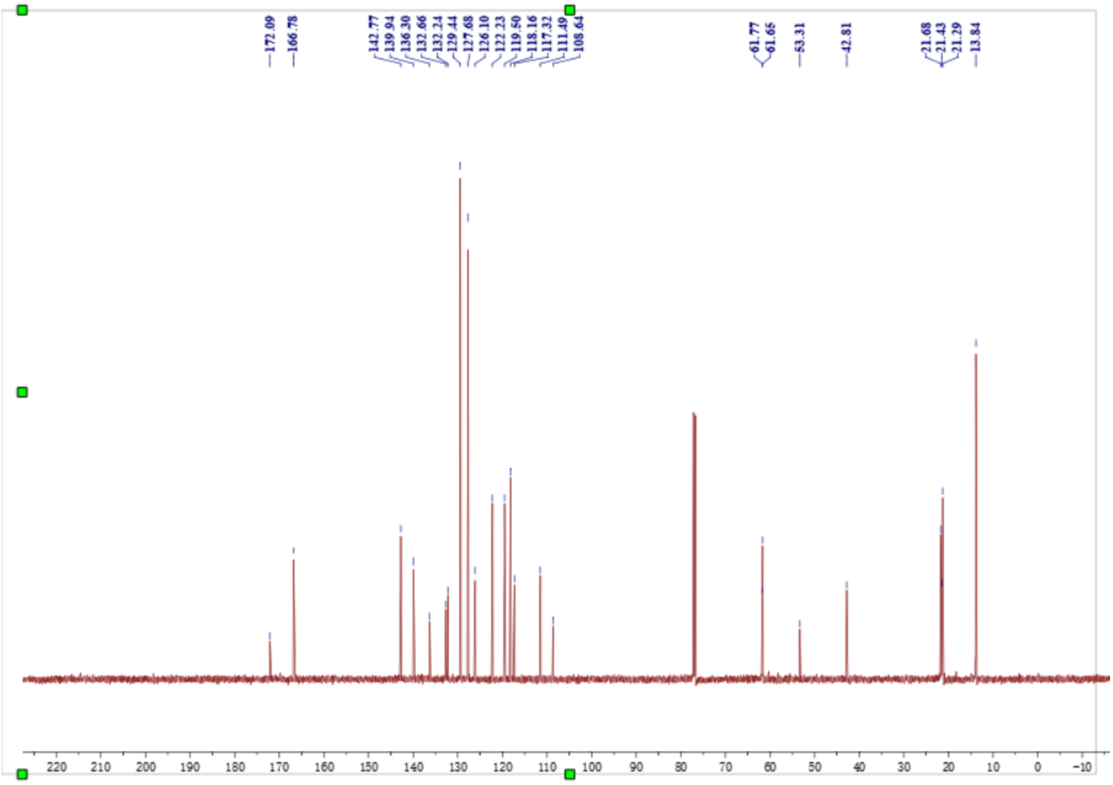

Ethyl(S,E)-2-(3-(4-fluorophenyl)acryloyl)-1-methyl-2,3,4,9-tetrahydro-1H-pyrido[3,4-b]indole-1-carboxylate)(**22**):

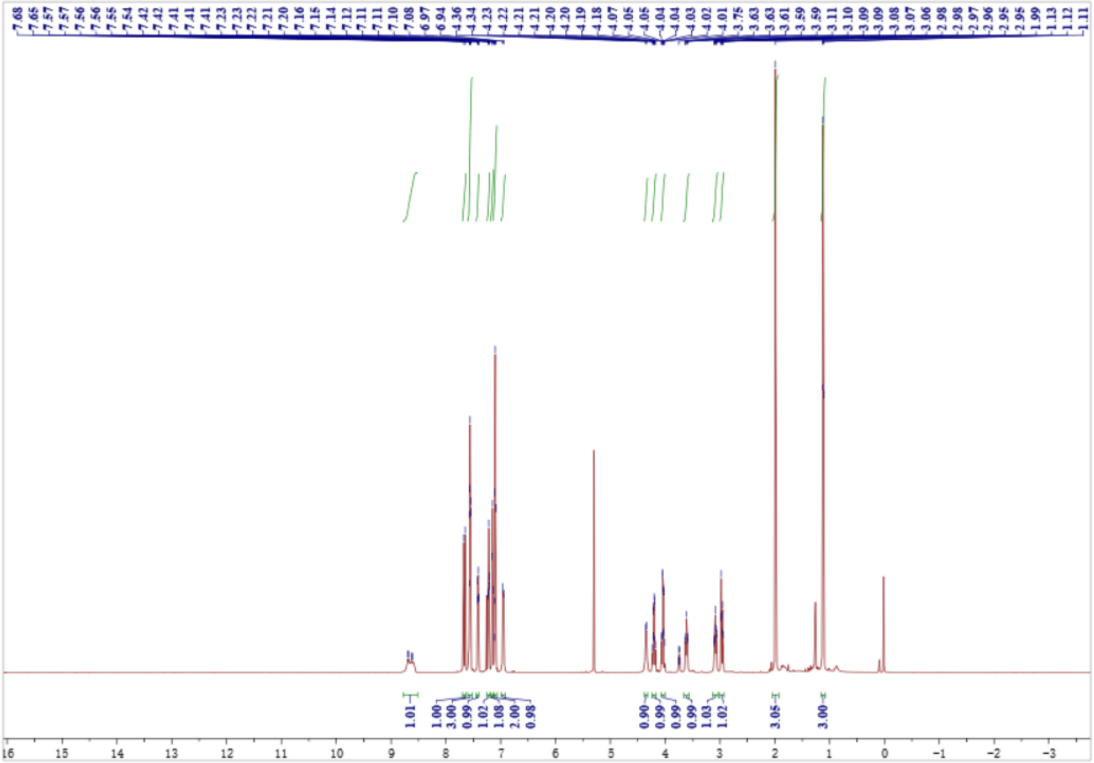

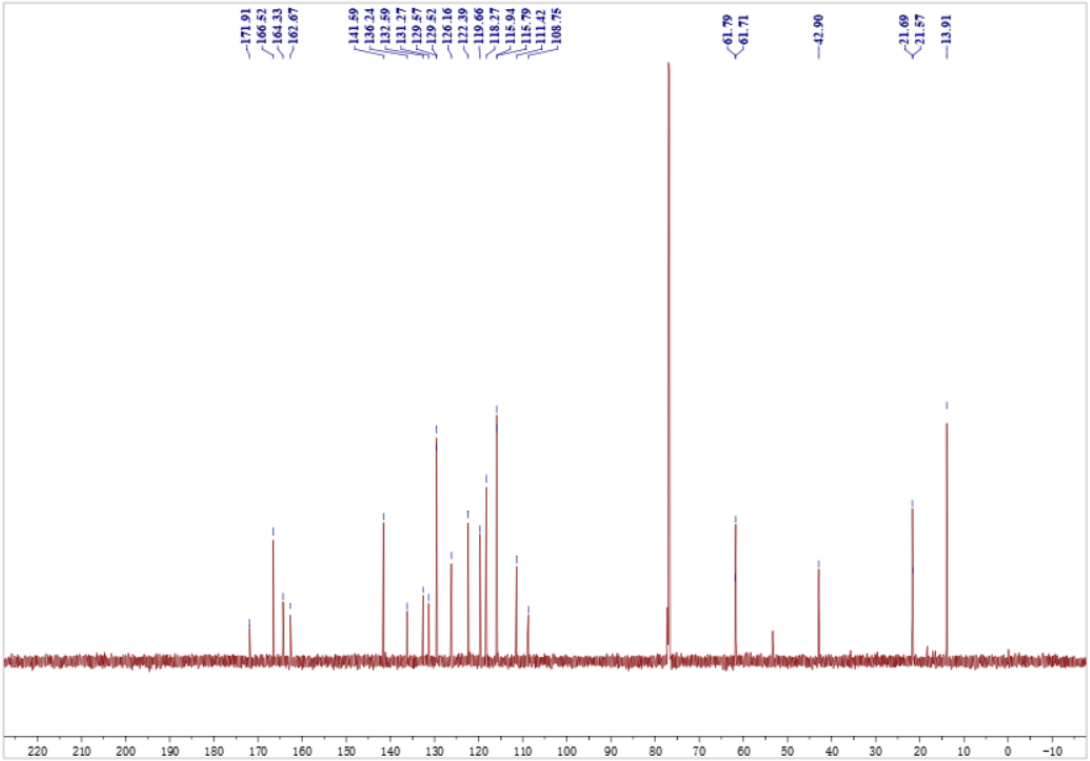

Ethyl(S,E)-1-methyl-2-(3-(naphthalen-2-yl)acryloyl)-2,3,4,9-tetrahydro-1H-pyrido[3,4-b]indole-1-carboxylate (**23**):

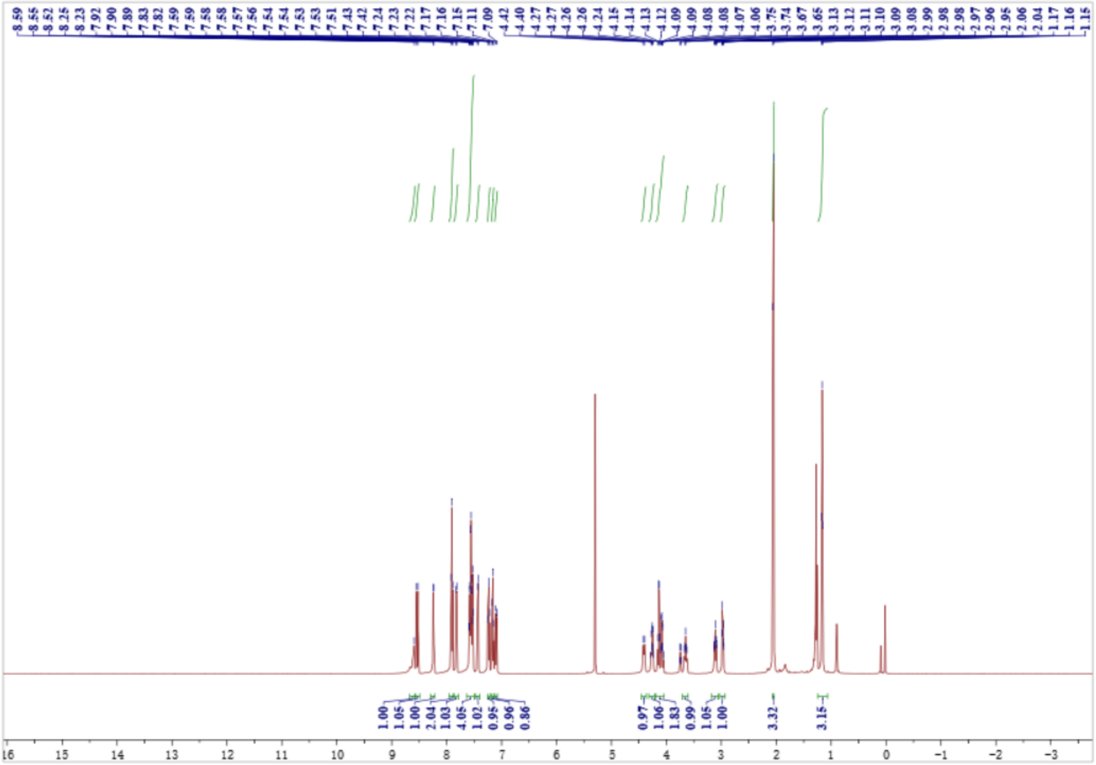

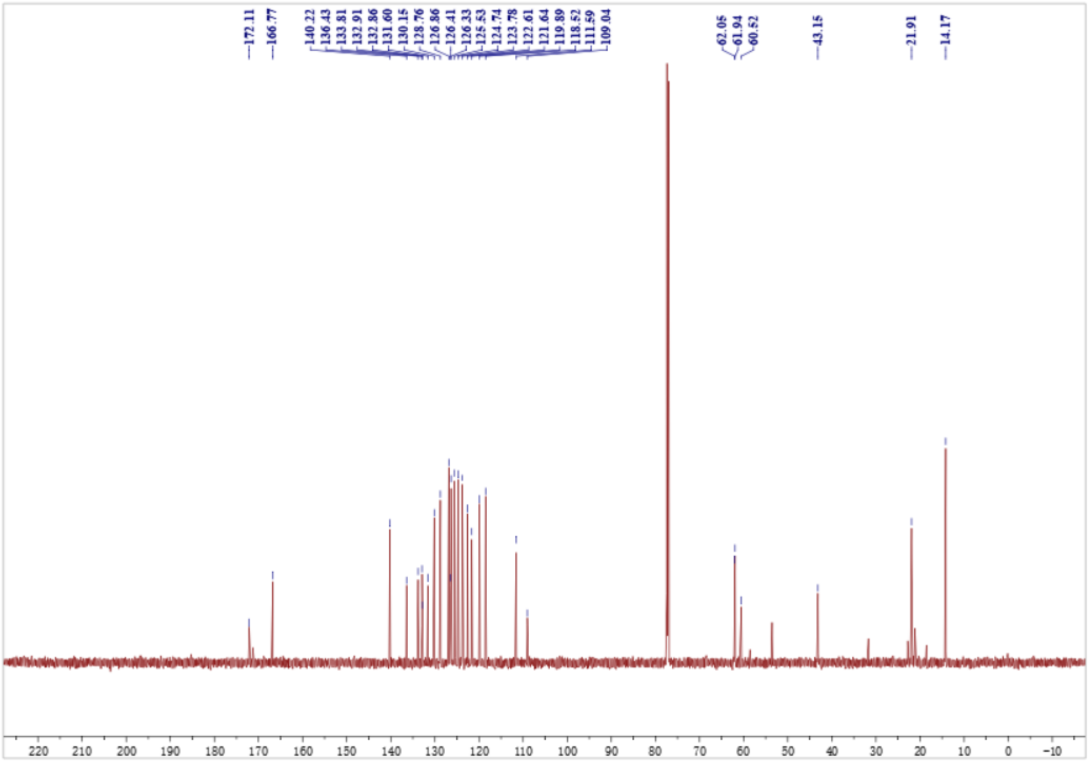

Ethyl(S)-2-(10-bromodecanoyl)-1-methyl-2,3,4,9-tetrahydro-1H-pyrido[3,4-b]indole-1-carboxylate (**24**):

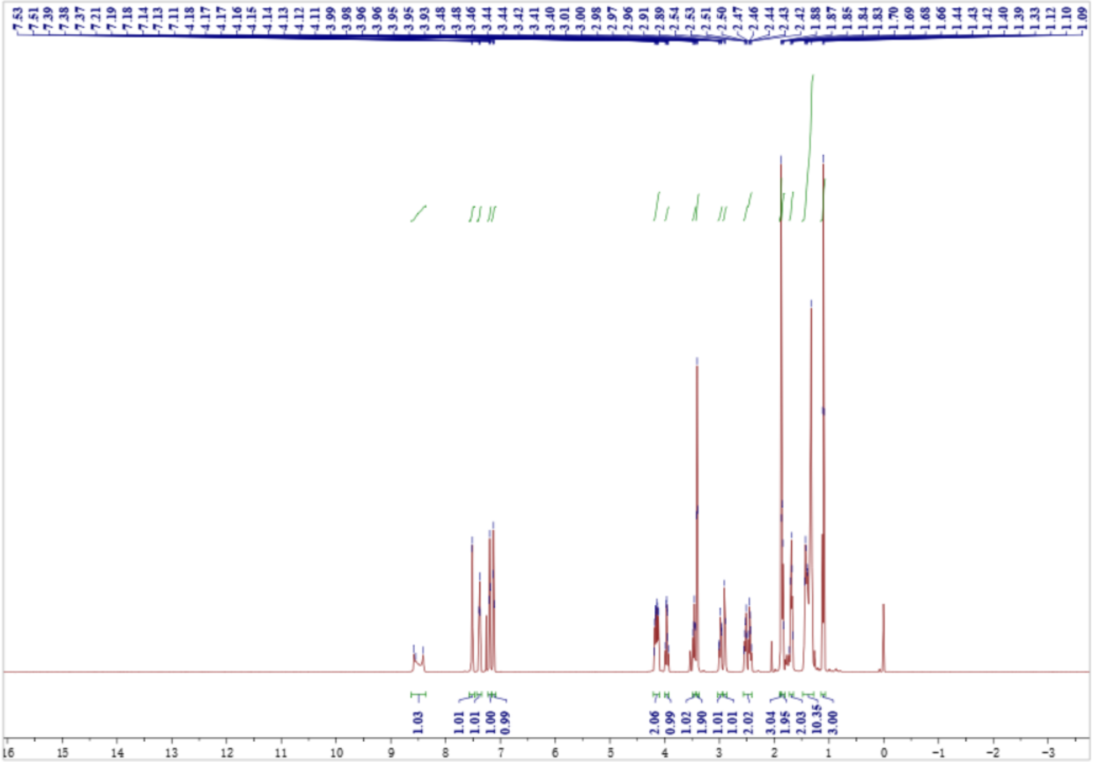

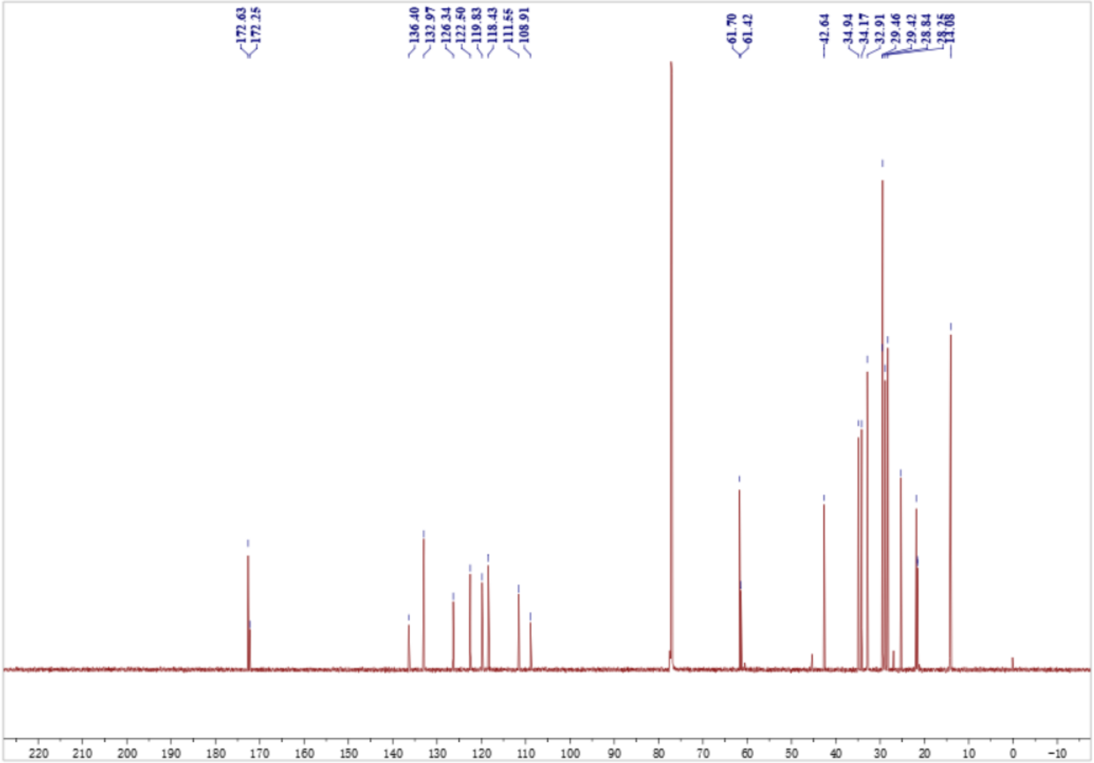

Ethyl(S)-2-(4-chlorobenzoyl)-6-methoxy-1-methyl-2,3,4,9-tetrahydro-1H-pyrido[3,4-b]indole-1-carboxylate (**25**):

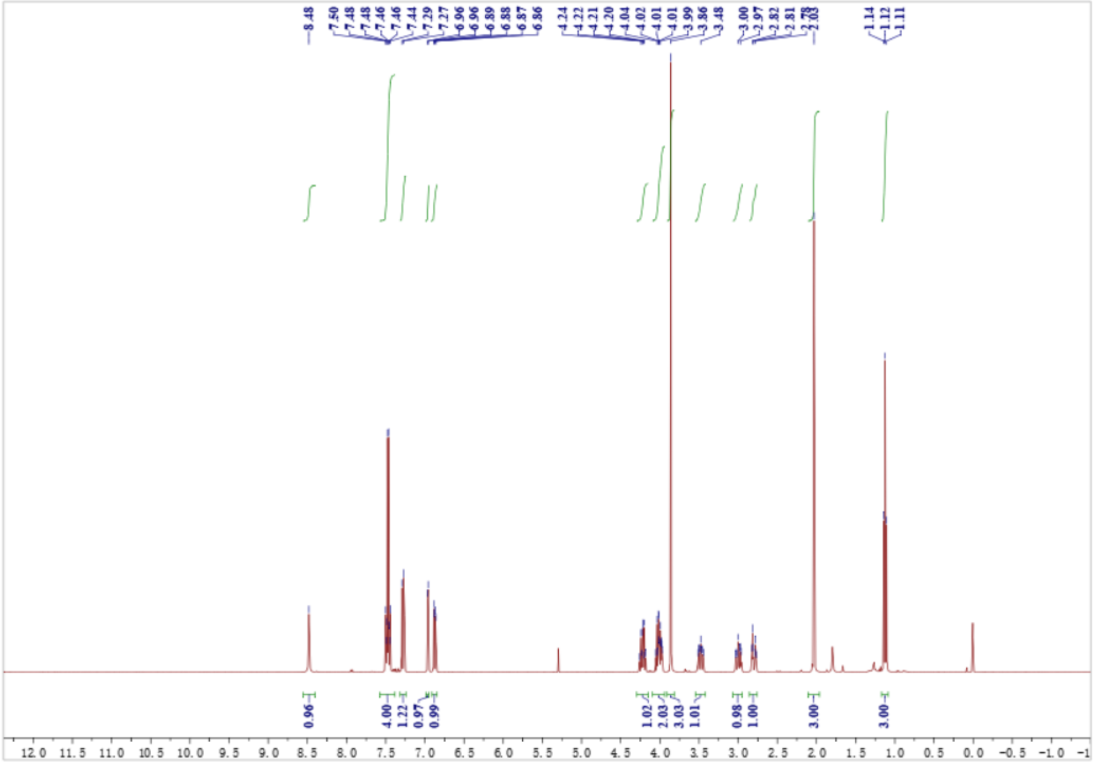

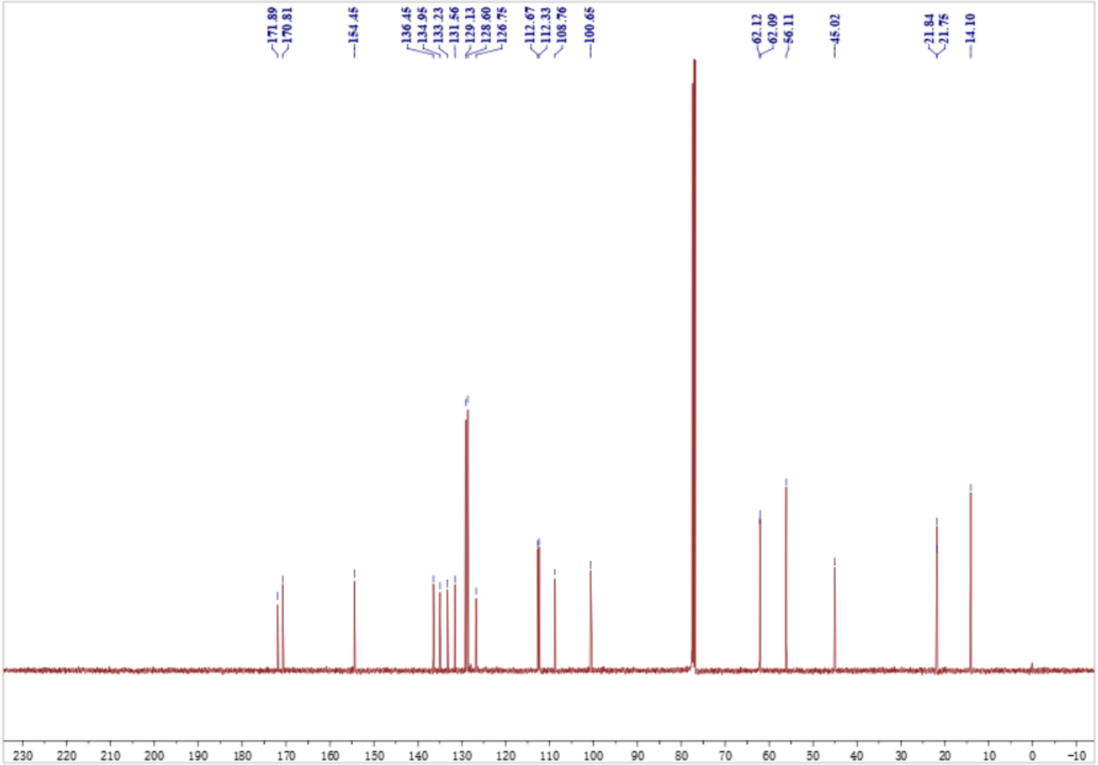

Ethyl(S,E)-2-(3-(4-chlorophenyl)acryloyl)-6-methoxy-1-methyl-2,3,4,9-tetrahydro-1H-pyrido[3,4-b]indole-1-carboxylate (**26**):

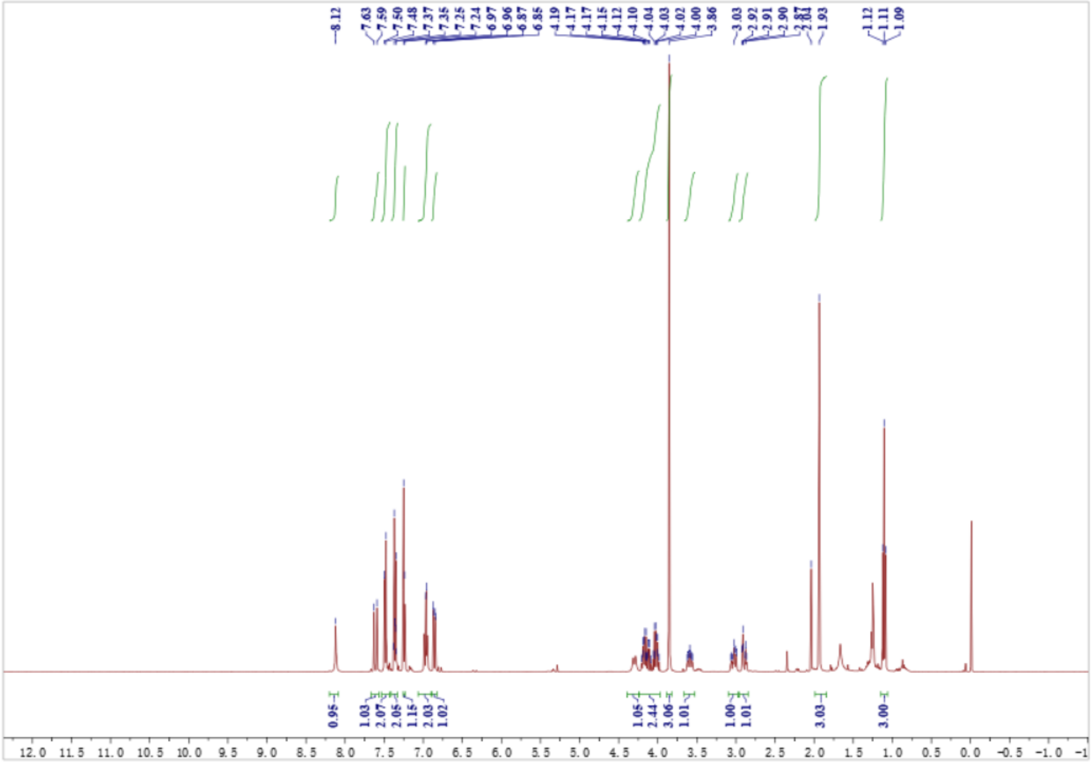

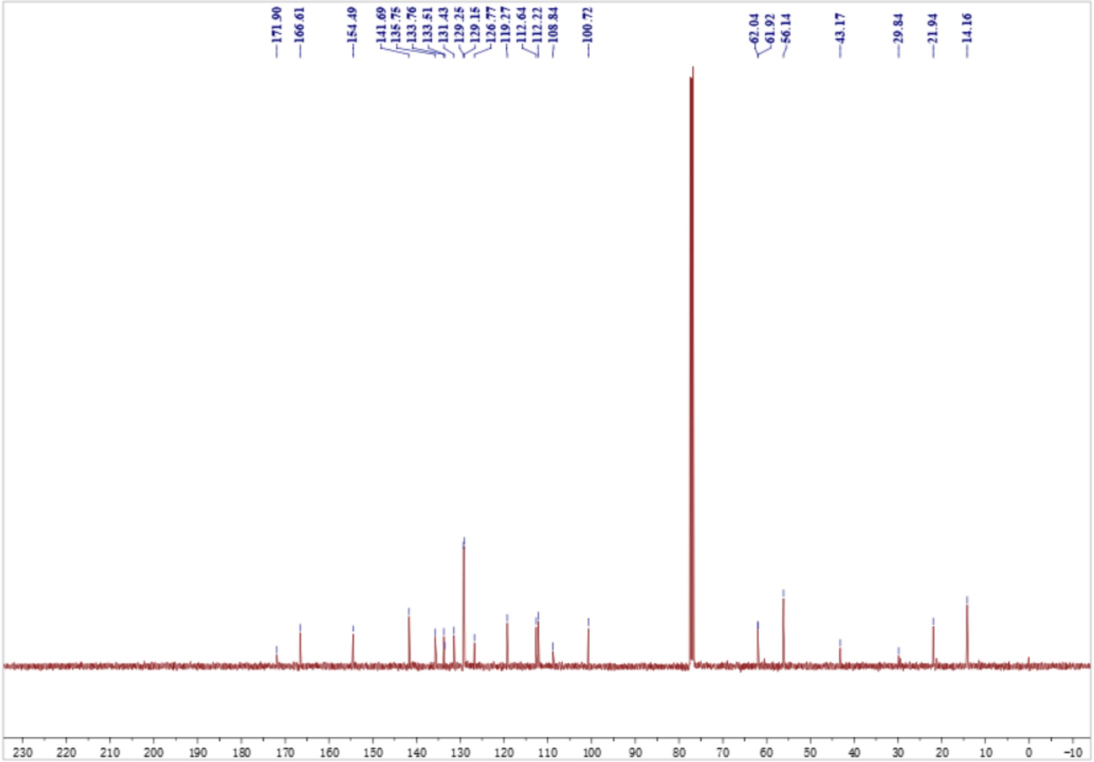

Ethyl(S,E)-6-methoxy-1-methyl-2-(3-(p-tolyl)acryloyl)-2,3,4,9-tetrahydro-1H-pyrido[3,4-b]indole-1-carboxylate (**27**):

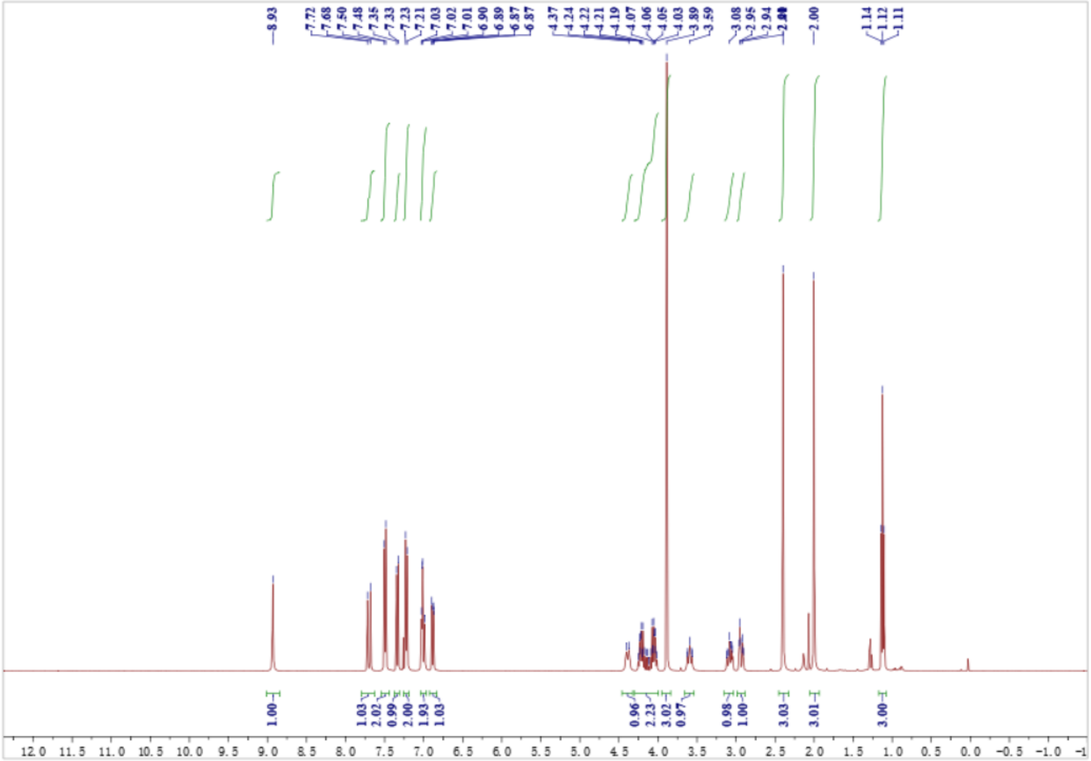

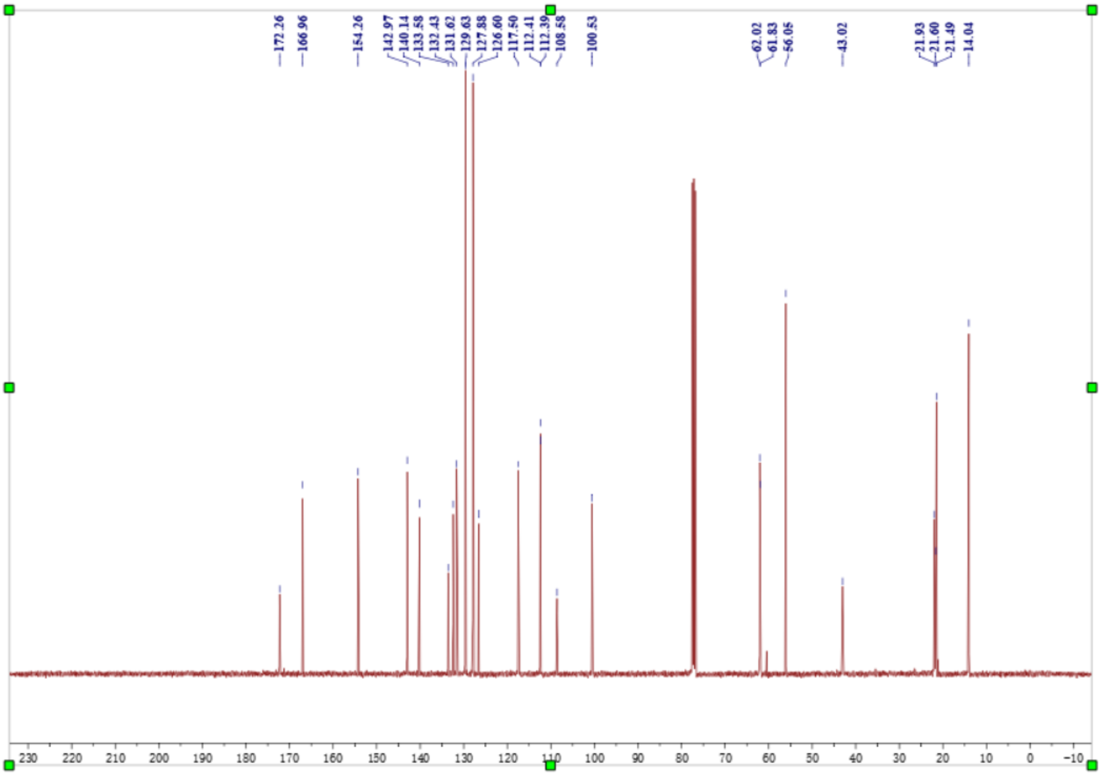

Ethyl(S,E)-2-(3-(4-fluorophenyl)acryloyl)-6-methoxy-1-methyl-2,3,4,9-tetrahydro-1H-pyrido[3,4-b]indole-1-carboxylate (**28**):

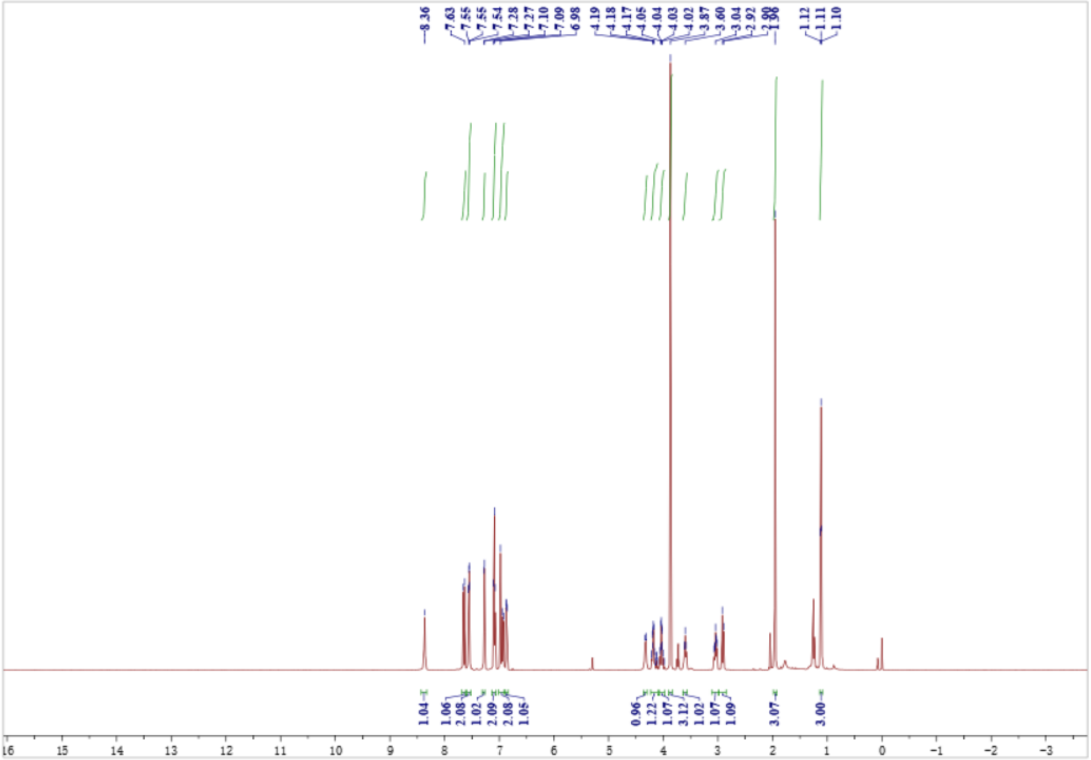

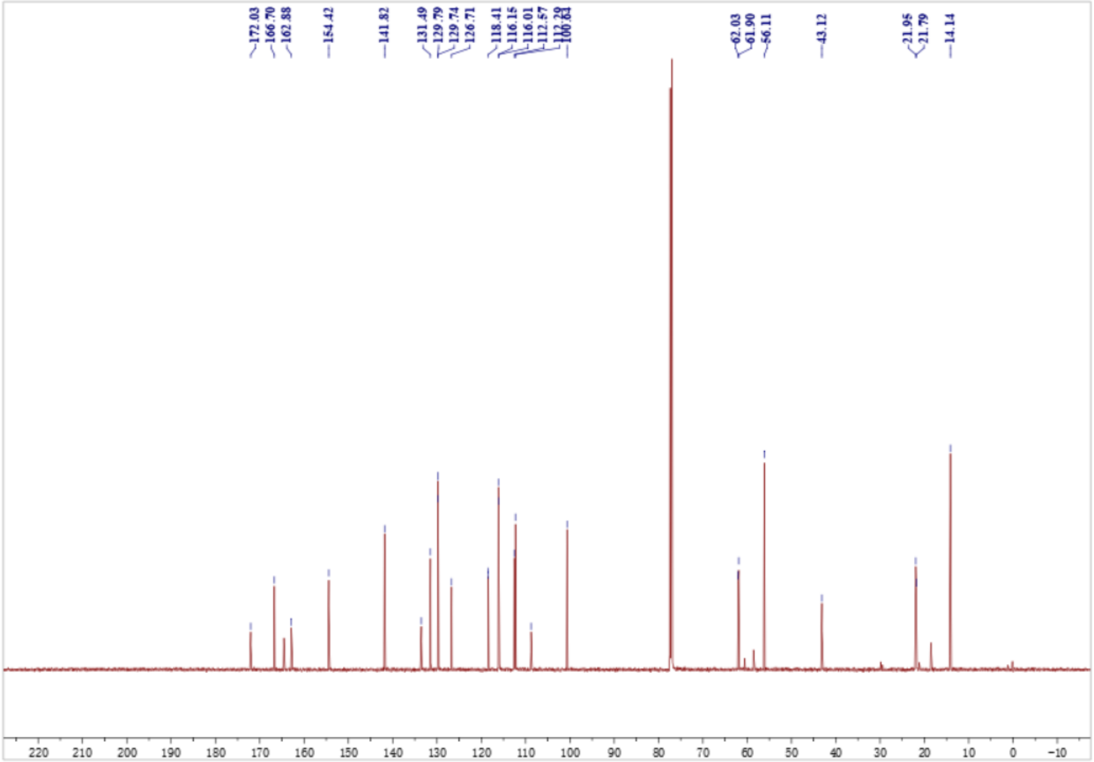

Ethyl(S)-2-(10-bromodecanoyl)-6-methoxy-1-methyl-2,3,4,9-tetrahydro-1H-pyrido[3,4-b]indole-1-carboxylate (**29**):

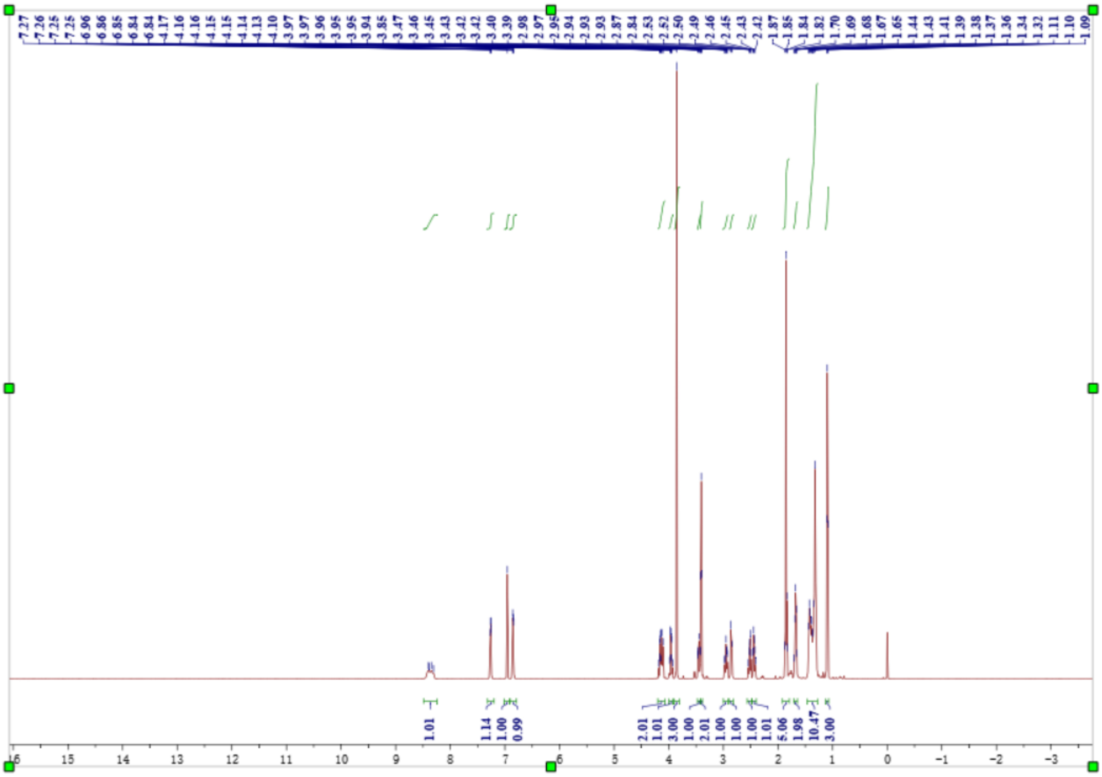

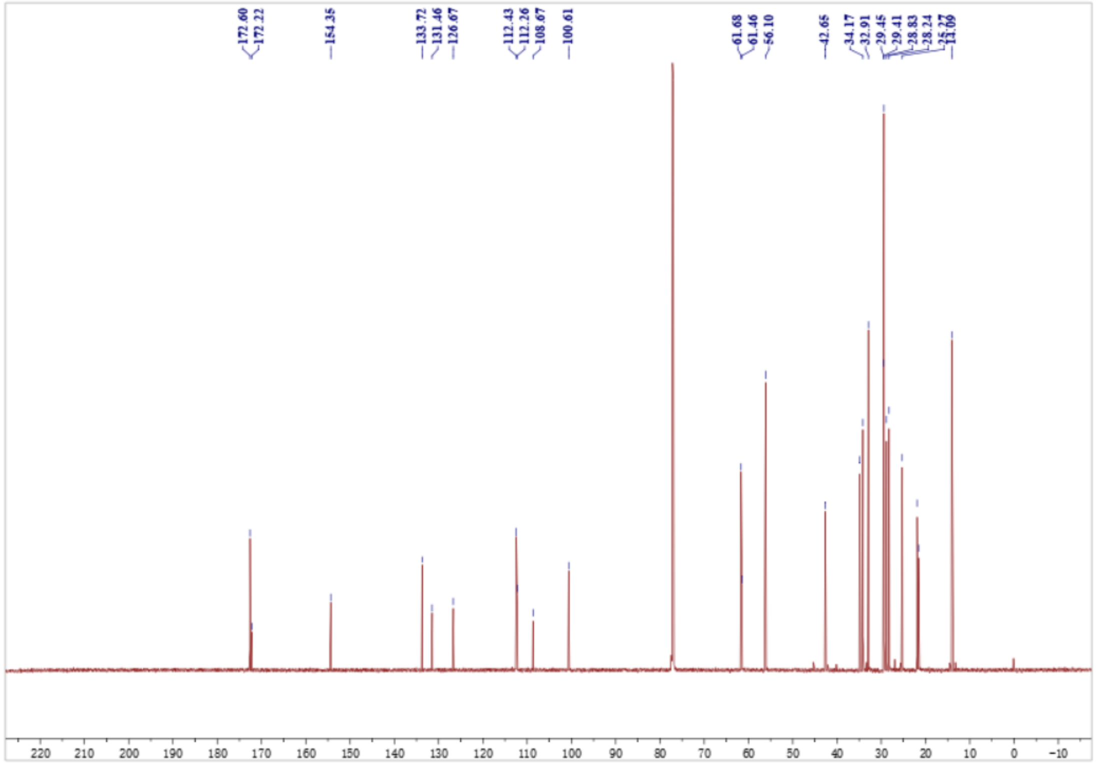

Ethyl(S)-2-(2-(4-methoxyphenyl)-2-oxoethyl)-1-methyl-2,3,4,9-tetrahydro-1H-pyrido[3,4-b]indole-1-carboxylate (**30**):

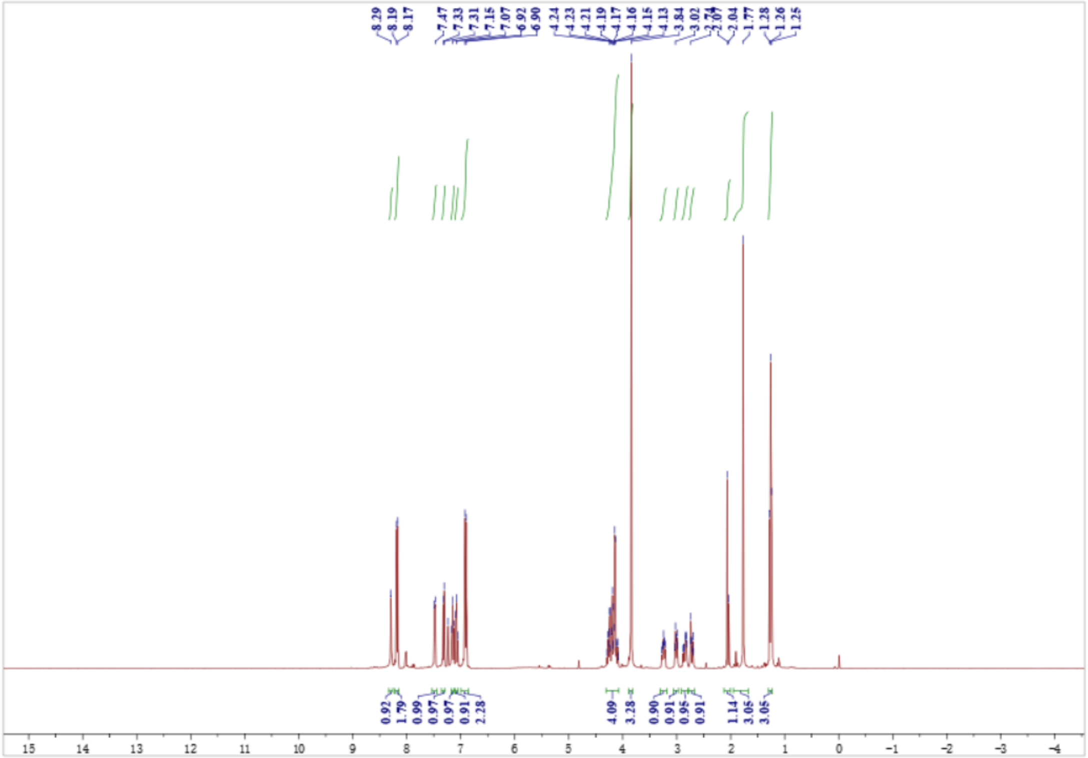

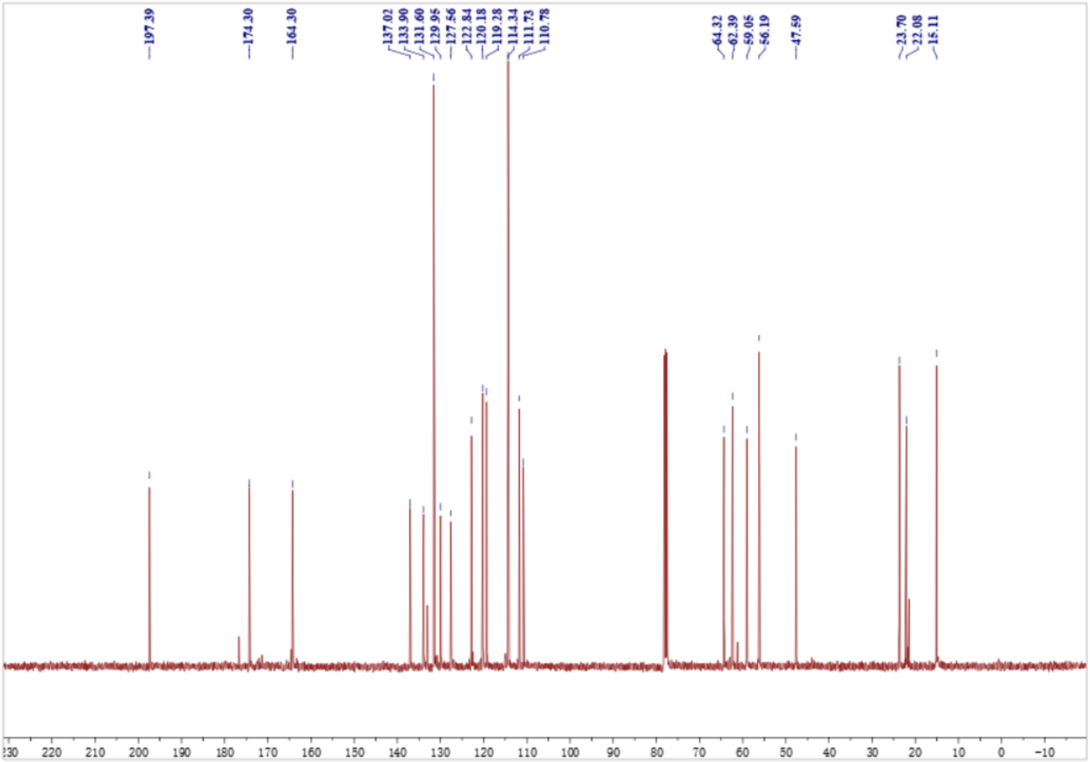

Ethyl(S)-1-methyl-2-(naphthalen-1-ylmethyl)-2,3,4,9-tetrahydro-1H-pyrido[3,4-b]indole-1-carboxylate (**31**):

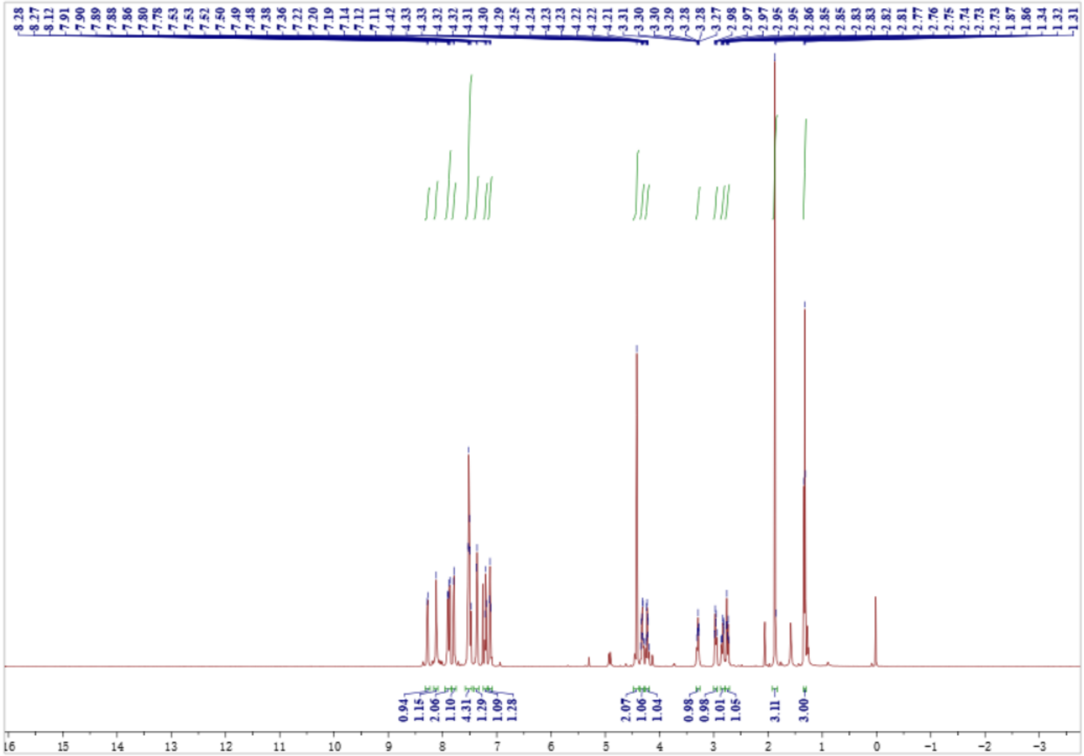

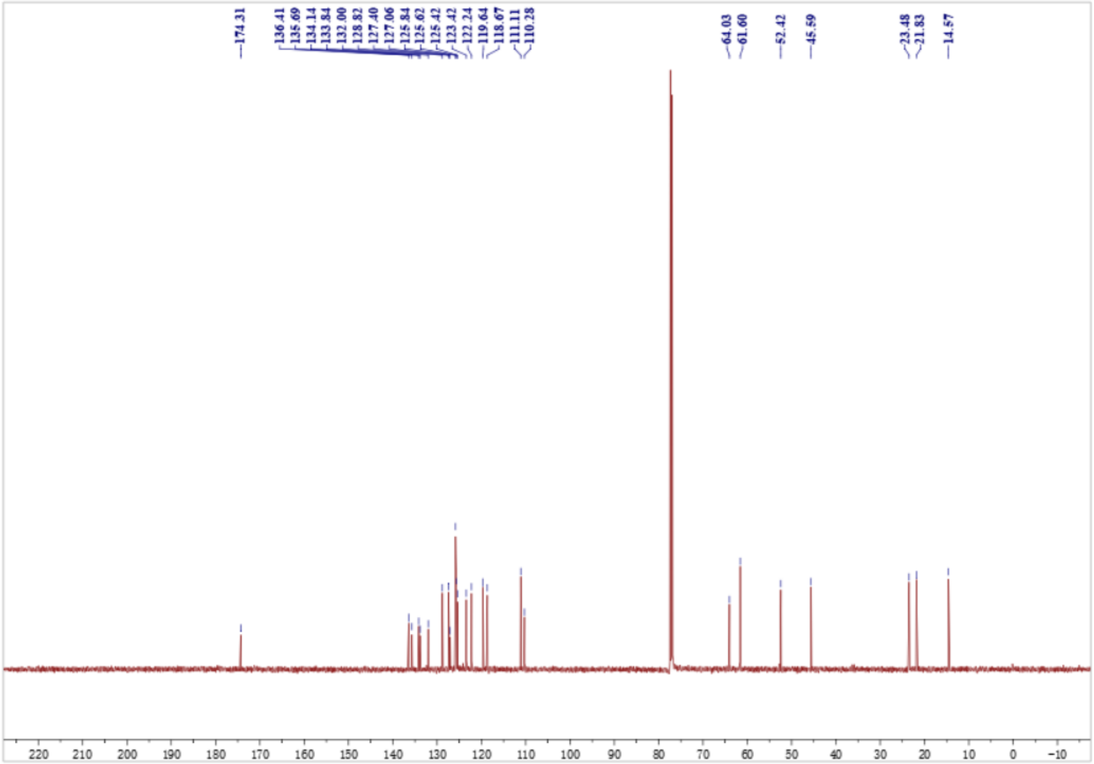

Ethyl(S)-1-methyl-2-(naphthalen-2-ylmethyl)-2,3,4,9-tetrahydro-1H-pyrido[3,4-b]indole-1-carboxylate (**32**):

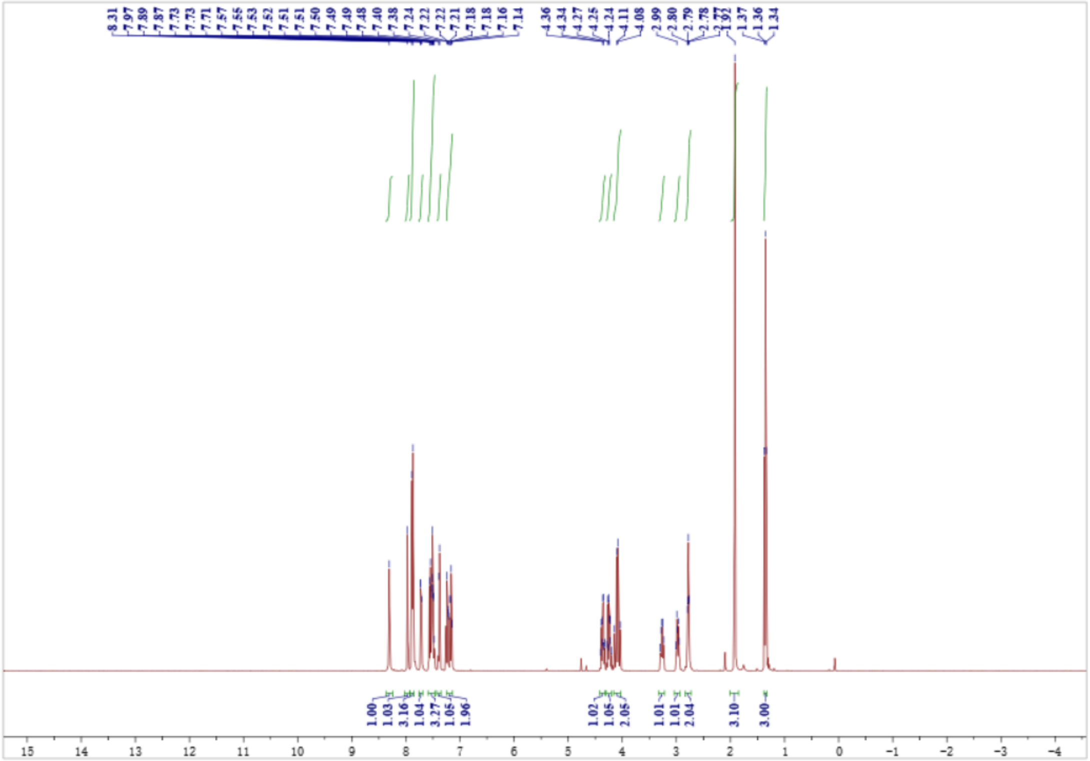

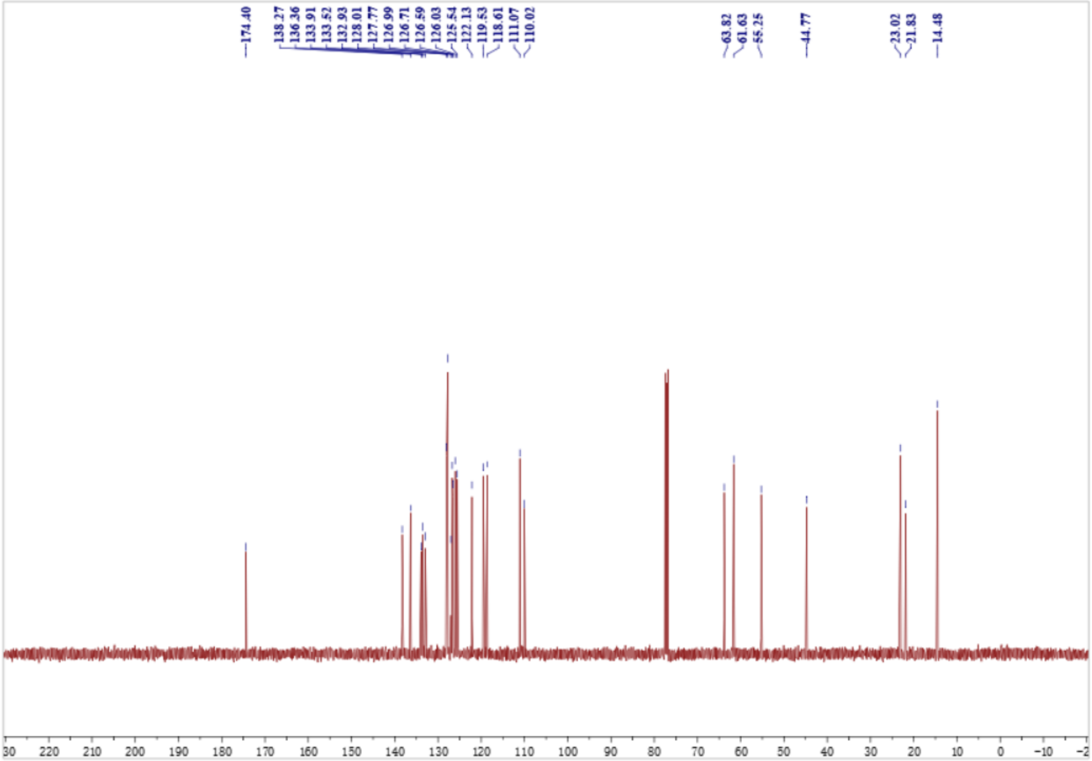

Ethyl(S)-6-methoxy-2-(2-(4-methoxyphenyl)-2-oxoethyl)-1-methyl-2,3,4,9-tetrahydro-1H-pyrido[3,4-b]indole-1-carboxylate (**33**):

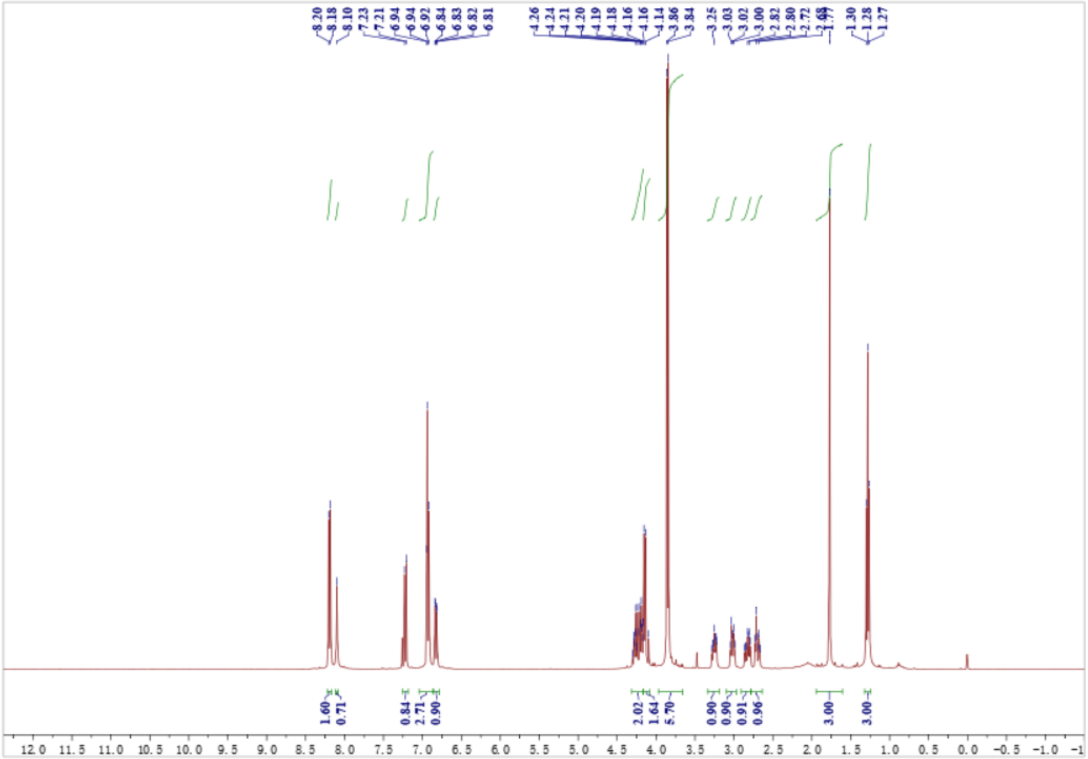

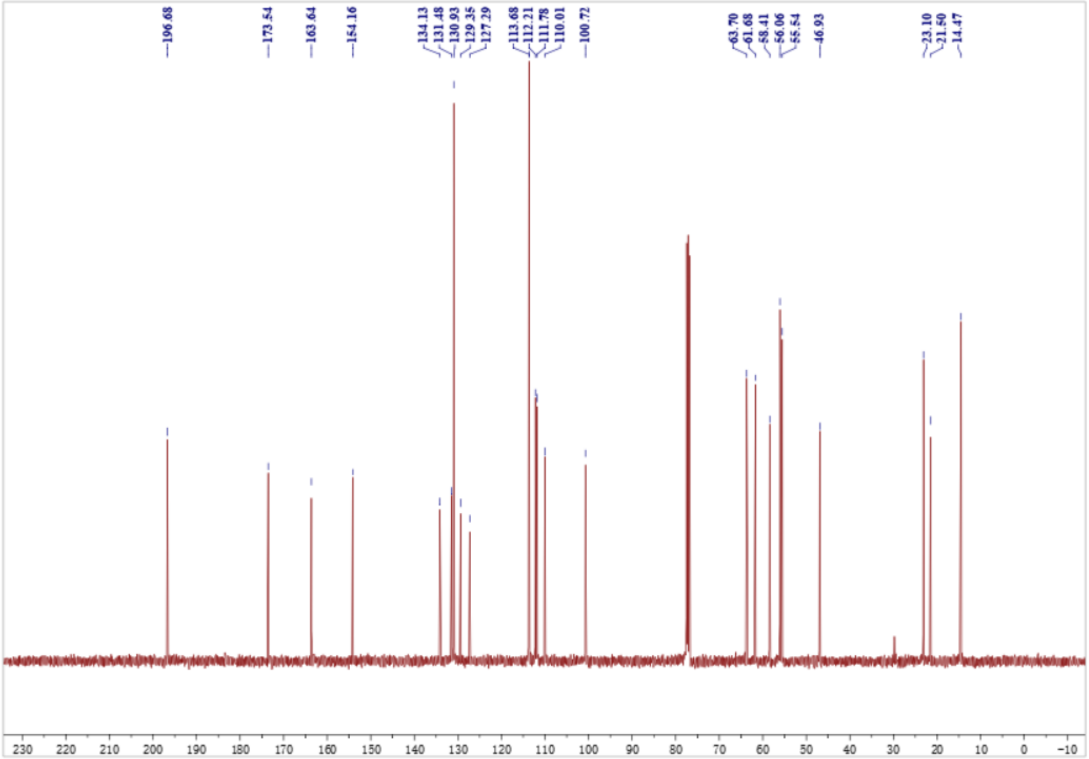

Ethyl(S)-6-methoxy-1-methyl-2-(naphthalen-2-ylmethyl)-2,3,4,9-tetrahydro-1H-pyrido[3,4-b]indole-1-carboxylate (**34**):

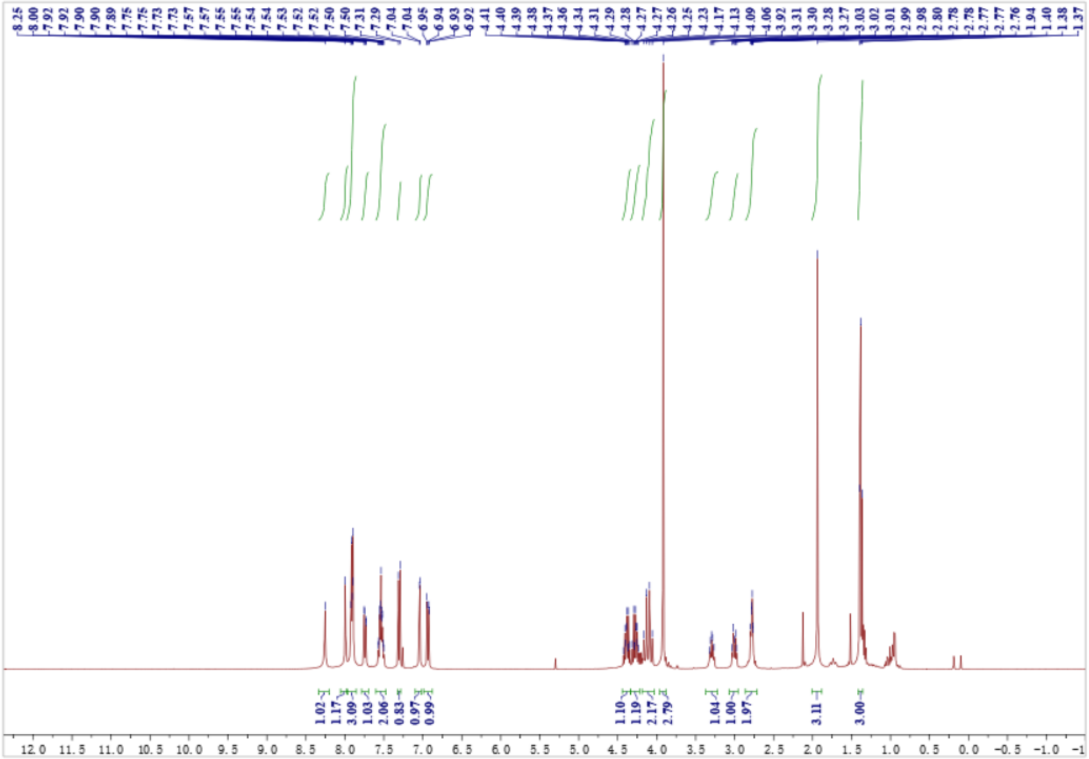

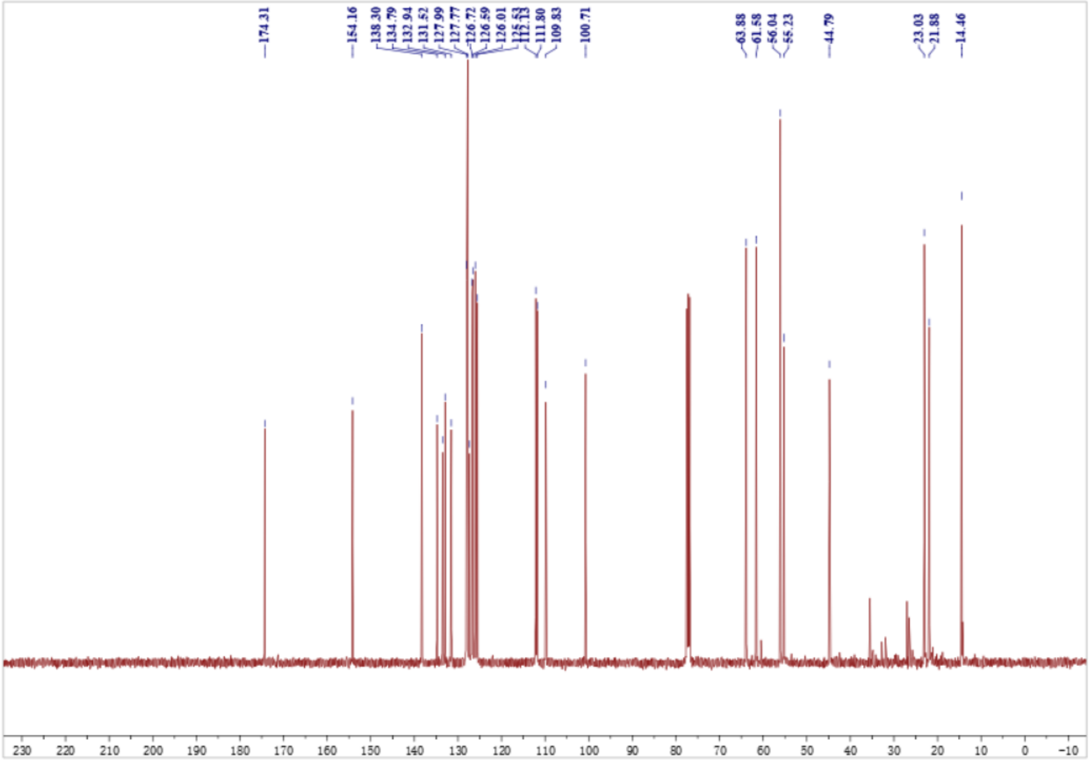

Supplement: Supplementary file 1 [file ijms-26-05396-s001.zip › supplementary material.pdf]
