# Supplementary figures and images for: Antitumor Activity of Tetrahydro-β-carboline Derivatives via Inhibition of Kinesin Spindle Protein: Validation by Molecular Docking, Molecular Dynamics, and In Vitro Assays
Source: Int J Mol Sci. 2025 Jun 4;26(11):5396. doi: 10.3390/ijms26115396 (PMC12154573; doi:10.3390/ijms26115396)

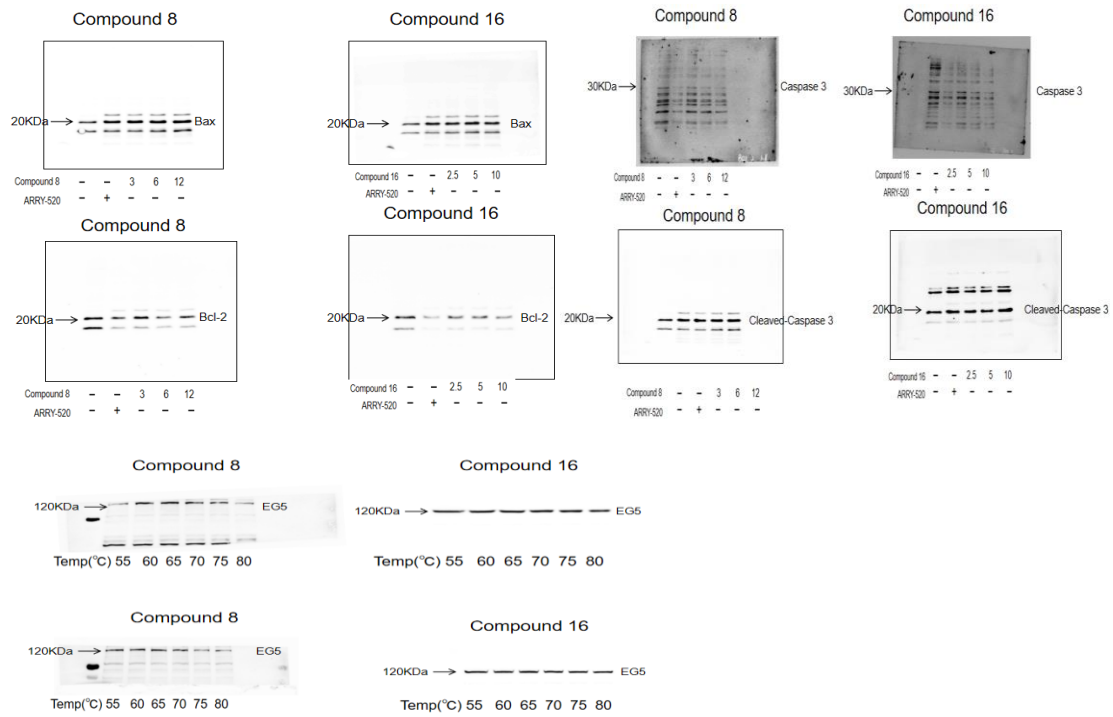

Immunoblot grayscale supplementary figure 1 :Immunoblot original map.

Supplement: Supplementary file 1 [file ijms-26-05396-s001.zip › immunoblott.pdf]
